# Outcome reporting in randomized trials of medicine–food homology interventions for cardiometabolic disease: a scoping review

**DOI:** 10.3389/fnut.2026.1937261

**Published:** 2026-10-12

**Authors:** Jianguo Xu, Qingyong Zheng, Tengfei Li, Ming Liu, Min Tian, Yonghong Zhu, Yingying Kang, Jinhui Tian, Ping Chen

**Affiliations:** 1 Evidence-Based Medicine Center, School of Basic Medical Sciences, Lanzhou University, Lanzhou, China; 2 Population Health Research Institute, Hamilton, ON, Canada; 3 Center for Biomedical Data Science, Duke-NUS Medical School, National University of Singapore, Singapore, Singapore; 4 School of Nursing, Chinese Academy of Medical Sciences & Peking Union Medical College, Beijing, China; 5 Lee Kong Chian School of Medicine, Nanyang Technological University, Singapore, Singapore; 6 Department of Pharmacy, Gansu Provincial Hospital, Lanzhou, China; 7 Gansu Pharmaceutical Group Science and Technology Research Institute, Lanzhou, China; 8 Key Laboratory of Evidence-Based Medicine of Gansu Province, Lanzhou University, Lanzhou, China; 9 Health Management Center, Gansu Provincial Hospital of Traditional Chinese Medicine, Lanzhou, China

**Keywords:** cardiometabolic disease, functional foods, medicine-food homology, outcome reporting, randomized controlled trials, scoping review

## Abstract

**Background:**

Substances classified as medicine-food homology (MFH) are tested as dietary interventions for cardiometabolic disease. The usefulness of this fast-growing evidence depends on whether trials choose, measure, and report outcomes in ways that can be compared and combined.

**Objective:**

To map outcomes reported in randomized controlled trials (RCTs) of MFH substances primarily addressing cardiometabolic health identified through four selected databases, describing their distribution across substances, clinical interpretation, and signals of reporting completeness.

**Methods:**

Following PRISMA for Scoping Reviews, we searched four databases and included RCTs of MFH substances on the review’s 110-item operational list with a cardiometabolic focus. Outcomes were charted verbatim, coded inductively into domains, and grouped as clinical events and mortality (T1), intermediate cardiometabolic measures (T2), or mechanistic biomarkers (T3), with other outcomes retained separately. Findings were summarized descriptively.

**Results:**

We included 416 studies reporting 2,231 outcome records (median 5 per study). Reporting concentrated on glycaemic (82.9% of studies), lipid (62.5%), anthropometric (53.4%), and blood-pressure (34.4%) domains, focusing heavily on five substances. The conventional lipid panel was commonly reported. Glycaemic measures covered different constructs and timescales; fasting glucose and HbA1c appeared in 50.1 and 31.3% of glycaemic studies, respectively. Of all outcome records, 0.7% were clinical events or mortality, 72.2% intermediate cardiometabolic measures, and 18.0% mechanistic biomarkers; 97.8% of studies reported no clinical event or mortality outcome. Among 328 studies naming a primary outcome, 165 (50.3%) named more than one.

**Conclusion:**

Outcomes in the identified MFH cardiometabolic RCTs are concentrated in a few intermediate domains, with variation in measurement and primary-outcome designation. Consistent definition, prospective registration, and clear primary-outcome reporting, adapted to study purpose and duration, would improve evidence usability.

## 1 Introduction

Cardiometabolic diseases, comprising cardiovascular disease and type 2 diabetes mellitus (T2DM) together with their principal risk states of obesity, hypertension, and dyslipidaemia, remain the leading contributors to global morbidity and mortality. The Global Burden of Disease (GBD) study estimated approximately 19 million cardiovascular deaths and more than 600 million prevalent cases worldwide, about a quarter of all deaths, with a large share attributable to modifiable metabolic and dietary risks (1, 2). Type 2 diabetes alone now affects more than half a billion adults worldwide (3). Because diet is among the most modifiable of these risks, food-based interventions occupy a central place in the prevention and long-term management of cardiometabolic disease, and interest in them spans clinical nutrition, pharmacology, and public health (2, 4).

Within this space, substances classified as medicine–food homology (MFH) have attracted growing scientific attention. MFH, a concept rooted in traditional Chinese medicine (TCM), denotes natural substances with a dual identity as both food and remedy. In China the concept is given an operational boundary by an official catalogue maintained by the National Health Commission, which lists substances formally recognized as both food and medicine and thereby distinguishes MFH from the broader and looser categories of functional foods or nutraceuticals (5–7). Many MFH substances, among them curcumin-bearing turmeric, cinnamon, ginseng, ginger, and saffron, are now the subject of randomized controlled trials (RCTs) in cardiometabolic populations, and meta-analyses and mechanistic reviews have catalogued their candidate effects across glycaemic, lipid, inflammatory, and vascular pathways (5, 6, 8–11). The number of such trials has risen steeply over the past decade.

The value of a rapidly expanding evidence base depends not only on how many trials are conducted but on whether they measure and report outcomes in ways that can be compared and combined. When trials of similar interventions select different outcomes, define the same outcome differently, or measure it at different timepoints, the resulting evidence becomes difficult to synthesize (12, 13), vulnerable to selective reporting (14, 15), and prone to research waste (12). These concerns motivated the core outcome set (COS) movement, which seeks an agreed minimum set of outcomes to be measured and reported in all trials of a given condition (16, 17). In the adjacent field of TCM for hyperlipidaemia, a systematic review documented substantial variability in reported outcomes, and a condition-specific COS was subsequently developed (18, 19). No comparable map exists for MFH substances considered as a class across the breadth of cardiometabolic conditions, even though this is precisely the level at which clinicians, guideline developers, and patients evaluate evidence regarding food-based cardiometabolic interventions.

How outcomes relate to patient-important endpoints matters as much as how consistently they are reported. Most trials of foods and nutrients use surrogate or intermediate markers rather than clinical events, and the interpretive weight that a surrogate can bear depends on whether it has been validated as a reliable predictor of patient-important outcomes (20, 21). The clinical interpretation of measures such as low-density lipoprotein cholesterol, glycated hemoglobin, or oxidative-stress markers depends on the outcome, population, and intervention under study. Distinguishing clinical endpoints, intermediate measures, and mechanistic markers is therefore essential to interpreting what a body of MFH trials actually demonstrates, yet syntheses of the field rarely make this distinction explicit, and the proliferation of plausible mechanisms in nutrition research makes it especially consequential here.

To address this gap, we conducted a scoping review to map the outcome reporting landscape in randomized trials of MFH interventions primarily addressing cardiometabolic health retrieved through four selected databases. We aimed to examine the consistency of outcome measurement and describe the clinical meaning of the reported outcomes. By providing an empirical baseline of current reporting practices, this study seeks to inform the design, appraisal, and possible future standardization of trials in this growing field (22).

## 2 Methods

We conducted this scoping review following the Joanna Briggs Institute methodology for scoping reviews (23) and the framework of Arksey and O’Malley (24) as advanced by Levac and colleagues (25), and we report it in accordance with the PRISMA extension for Scoping Reviews (PRISMA-ScR) (26). The review was not prospectively registered.

### 2.1 Eligibility criteria

Eligibility was framed using the PCC elements.

#### 2.1.1 Concept

We included RCTs of MFH substances, defined operationally by a 110-item list compiled from Chinese official and draft MFH catalogue materials (Supplementary material S1). Eligible interventions included the listed materials as whole foods, extracts, or derived single compounds. Where a plant part was specified, it had to correspond to the listed material. Extracts without a stated part were assessed using the reported source substance and preparation. All active ingredients of an intervention had to be listed substances. We excluded products containing any non-listed active ingredient, preparations of a non-listed species, and preparations explicitly using a plant part outside the listed material. Whole foods, extracts, and derived compounds were included to map outcome selection across MFH research; formulation types were described separately.

#### 2.1.2 Population and context

We included trials in participants of any age with, or at elevated risk of, a cardiometabolic condition: type 1 or type 2 diabetes, prediabetes, the metabolic syndrome, overweight or obesity, hypertension, dyslipidaemia, fatty liver disease, or established cardiovascular disease, and other populations studied for cardiometabolic risk. Healthy participants were eligible in studies of metabolic or postprandial responses. Eligible trials had a cardiometabolic primary-outcome focus or, when no primary outcome was specified, a cardiometabolic study objective.

#### 2.1.3 Types of studies

We included parallel-group, crossover, and factorial RCTs, encompassing pilot trials and acute single-dose postprandial designs. We excluded non-randomized and observational studies, animal and in-vitro studies, reviews, meta-analyses, protocols, conference abstracts without a full report, and pharmacokinetic-only studies.

### 2.2 Search strategy

We searched PubMed/MEDLINE, Embase, Web of Science Core Collection, and the Cochrane Central Register of Controlled Trials from inception, with no language or date restriction, supplemented by backward citation tracking. To ensure the timeliness of the evidence base and minimize the gap between search and final synthesis (27), the initial search (conducted in August 2025) was updated in June 2026 prior to manuscript submission. Each search combined a catalogue-derived lexicon of the 110 listed substances with cardiometabolic condition terms and a validated randomized-trial filter (28). This review maps the studies retrieved through the four selected databases; search sensitivity was not formally assessed. Full strategies for each database are provided in Supplementary material S1.

### 2.3 Selection of sources of evidence

All identified records were imported into EndNote reference management software and de-duplicated. Two reviewers independently screened the titles and abstracts of all unique records to identify potentially eligible studies. Subsequently, the full texts of these potentially eligible reports were retrieved and independently assessed against the pre-specified inclusion and exclusion criteria. Any conflicts were resolved through discussion with a third reviewer.

### 2.4 Data charting

Two reviewers independently charted data using a standardized form (29), recording bibliographic identifiers, registration number, the MFH substance with its botanical source, duration, population, design, randomized sample size, country, comparator, blinding, and reported outcomes transcribed verbatim together with their stated instrument or definition, timepoints, and reported hierarchy (primary, secondary, or unspecified). Any discrepancies were resolved by consensus. A report was a publication, and reports describing the same trial were linked using registration identifiers, participant characteristics, intervention and follow-up details. Linked reports contributed one study to the map; experiments reported together were retained in the same record. Repeated outcomes and overlapping panels were combined across linked reports, retaining additional components. The study characteristics and individual outcome records are provided in Supplementary Table S1.

An outcome record represented a reported measure, panel, or composite endpoint. Repeated timepoints for the same record were counted once. Components reported separately were retained as separate records. A study contributed once to each domain it reported; records spanning several domains contributed to each relevant domain for study-level coverage and to their first assigned domain for outcome-level group counts.

### 2.5 Classification of outcomes

Verbatim outcomes were coded inductively, with categories derived from the charted text rather than imposed in advance. Drawing on the distinction between clinical outcomes and surrogate endpoints (20, 21), each outcome was assigned to one or more domains. Domains were grouped as T1, clinical events and mortality; T2, intermediate cardiometabolic measures, including risk factors and physiological measures; and T3, mechanistic biomarkers. Symptoms, function, quality of life, safety, and process outcomes were retained in separate domains. The full coding dictionary is provided in Supplementary material S2.

### 2.6 Data synthesis and reporting

Consistent with scoping-review methodology, we did not perform meta-analysis or formal risk-of-bias appraisal. We summarized the evidence descriptively as counts and percentages, at the study level (the proportion of studies reporting a given outcome domain or measure) and at the outcome-record level (the distribution of all charted records). For T1–T3 study counts, a study had to contain at least one outcome record bearing the corresponding group label. To characterize reporting practice we additionally examined, from the reports themselves, two completeness signals, a registration number reported and the explicit designation of a primary outcome, and their distribution across publication eras, and we tabulated the within-domain measures used for the two most frequently reported domains. A study was counted as designating a primary outcome when at least one outcome record was labeled primary. For linked reports, publication era was assigned using the earliest included report. Registration timing and multiplicity adjustments were not systematically assessed. Domain coverage was stratified by acute, repeated, mixed, or unclear dosing patterns and by formulation, using the charted intervention descriptions. Glycaemic measures were interpreted according to their physiological construct and measurement timescale. Glucose or insulin area-under-the-curve summaries included AUC, incremental AUC (iAUC), and netAUC, with each study counted once in this category.

## 3 Results

### 3.1 Study selection and characteristics

Database searching identified 14,639 records. After title and abstract screening and subsequent full-text assessment of 1,097 reports, 677 reports were excluded and 420 reports representing 416 studies were included (Figure 1). A complete list of the included studies is provided in Supplementary material S3, with study characteristics and outcome records in Supplementary Table S1. Representative reports excluded at the full-text stage, along with their specific reasons for exclusion, are detailed in Supplementary material S4. The included studies were published between 1995 and 2026, with output accelerating over time: 198 studies (47.6%) appeared in 2020 or later and a further 110 (26.4%) in 2015 to 2019 (Table 1; Figure 2a).

**Figure 1:**
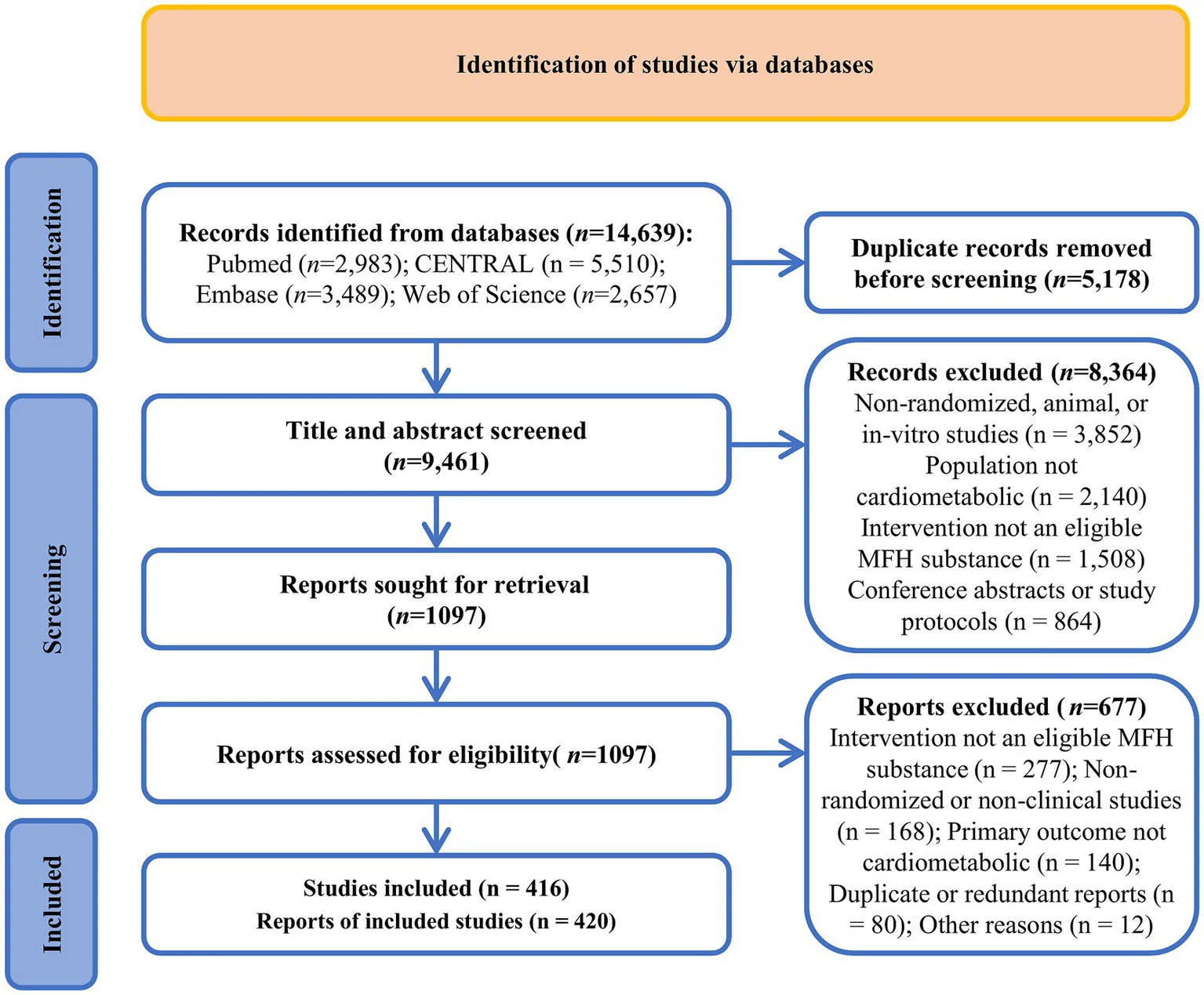
PRISMA-ScR flow diagram of study selection.

**Table 1:** Characteristics of the included studies.

Characteristic	*n* or value	% or range
Study design and conduct		
Parallel-group	306	73.6
Crossover	96	23.1
Factorial	10	2.4
Other/not specified	4	1.0
Randomized sample size, median (IQR)	56	33–80
Double-blind	244	58.7
<2015	108	26.0
2015–2019	110	26.4
2020+	198	47.6
Intervention exposure		
≤12 weeks (incl. Single acute dose)	328	78.8
of which acute-only studies	61	14.7
>12 weeks	74	17.8
Duration not clearly reported	14	3.4
Population/target condition		
Type 2 diabetes	108	26.0
Healthy	64	15.4
Overweight/obese	60	14.4
NAFLD	28	6.7
Metabolic syndrome	26	6.2
Other conditions	130	31.2
Intervention (MFH substance)		
Curcumin/turmeric	105	25.2
Cinnamon	54	13.0
Ginseng	45	10.8
Ginger	29	7.0
Saffron/crocin	27	6.5
Other substances	156	37.5
Outcome reporting		
Outcome records per study, median (range)	5	1–22
Studies reporting ≥ 6 outcome records	150	36.1
Primary outcome explicitly designated	328	78.8
≥ 2 outcomes labeled primary (of 328 naming any)	165	50.3
Registration number reported	243	58.4
Reports ≥ 1 intermediate (T2) outcome	402	96.6
Reports ≥ 1 mechanistic (T3) outcome	202	48.6
Reports ≥ 1 clinical event/mortality (T1) outcome	9	2.2

**Figure 2:**
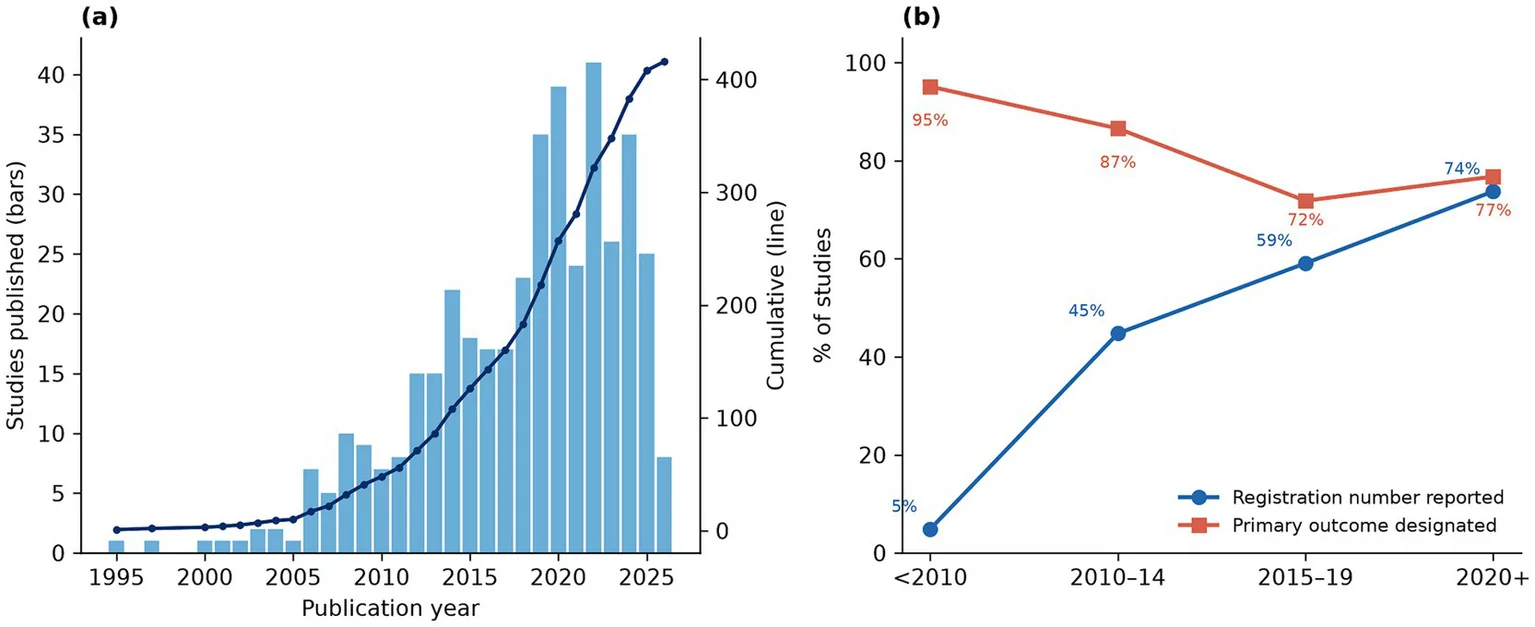
Temporal trends in publication volume and reporting practice. **(a)** Annual and cumulative number of included studies by the year of the earliest included report for each study. **(b)** Percentage of studies reporting a registration number and designating a primary outcome, grouped by publication era.

The evidence concentrated on a small number of substances. Curcumin-bearing turmeric (105 studies, 25%), cinnamon (54, 13%), ginseng (45, 11%), ginger (29, 7%), and saffron or its constituents crocin and crocetin (27, 6%) together accounted for 260 studies (63%). At the other extreme, of 38 distinct substances studied, 13 had been examined in only a single trial and a further 4 in only two, so almost half (17 of 38) of the substances reaching trial were supported by no more than two trials each. These substances were, moreover, investigated by largely separate regional research communities (Table 2): saffron and ginger almost entirely in the Middle East, ginseng in East Asia and North America, and mulberry chiefly in East Asia. T2DM was the most studied condition (108 studies, 26%), followed by healthy or mixed populations (64, 15%) and overweight or obesity (60, 14%); comparatively few trials enrolled patients with established cardiovascular disease (20, 5%). Most studies used a parallel-group design (306, 74%), were placebo-controlled (306, 74%), and were short: 328 studies (78.8%) ran for 12 weeks or less, including 61 (15%) that tested a single acute dose, while 74 (17.8%) ran longer than 12 weeks. The median randomized sample size was 56 participants (interquartile range 33 to 80). Reporting of methodological safeguards was incomplete: a double-blind design was stated by 244 studies (59%), but blinding was not clearly reported by 152 (37%); a trial registration number was reported by 243 (58%); and a primary outcome was explicitly designated by 328 (79%).

**Table 2:** Geographic distribution of studies for the most-studied substances.

Substance	Middle East	East Asia	Europe	N America	South Asia	SE Asia	Other	Total
Curcumin/turmeric	56	8	8	6	9	7	11	105
Cinnamon	16	2	6	10	8	2	10	54
Ginseng	2	19	4	16	0	0	4	45
Ginger	20	1	0	1	2	0	5	29
Saffron/crocin	23	1	3	0	0	0	0	27
Mulberry	1	9	6	2	2	2	1	23
Honey	4	1	5	4	2	2	3	21

### 3.2 Reported outcomes and measurement

The 416 studies reported 2,231 outcome records, a median of 5 per study (range 1 to 22). Outcome counts were often high: 150 studies (36%) reported six or more outcomes and 40 (10%) reported ten or more. Designation of a primary outcome was often non-exclusive: among the 328 studies that labeled at least one outcome as primary, 165 (50.3%) labeled two or more as primary.

Reporting was dominated by a small set of biochemical and anthropometric domains (Figure 3). At the study level, glycaemic outcomes were reported by 345 studies (82.9%), lipid outcomes by 260 (62.5%), anthropometric or adiposity outcomes by 222 (53.4%), and blood-pressure or haemodynamic outcomes by 143 (34.4%). Mechanistic domains were also common: inflammatory or adipokine markers were reported by 130 studies (31.3%), liver-related outcomes by 80 (19%), and oxidative-stress markers by 75 (18%). Cardiac outcomes were uncommon, with a cardiac injury or function biomarker reported by 12 trials (3%) and a clinical event or mortality outcome by 9 (2%). The domains nominated as primary mirrored this distribution: glycaemic outcomes were a declared primary in 201 studies and lipid outcomes in 101, and mechanistic domains were also designated as primary, with oxidative-stress markers named as a primary outcome in 31 studies and inflammatory markers in 45.

**Figure 3:**
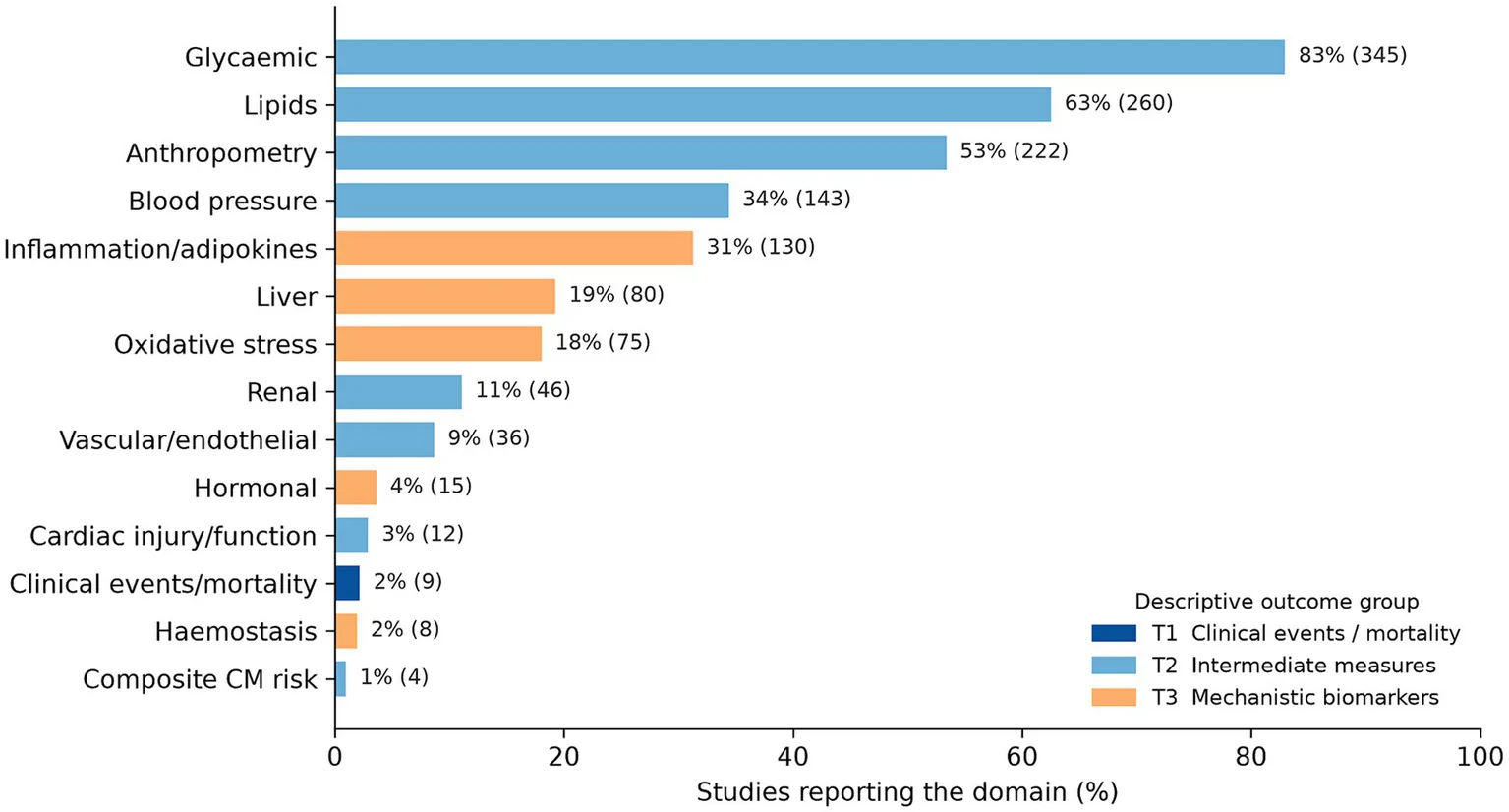
Distribution of reported outcome domains. Percentages represent the proportion of the 416 included studies reporting at least one outcome within each domain. Colors indicate the outcome group: T1, clinical events and mortality; T2, intermediate cardiometabolic measures; T3, mechanistic biomarkers.

Within the two most frequently reported domains, lipid reporting was relatively standardized: among the 260 studies reporting any lipid outcome, the four components of the conventional panel were reported by most, namely triglycerides (83.8%), high-density lipoprotein cholesterol (81.2%), low-density lipoprotein cholesterol (75.8%), and total cholesterol (75.4%), with additional measures such as apolipoproteins (3.8%) or lipoprotein(a) (1.9%) used only occasionally. Glycaemic reporting covered several constructs. Among the 345 studies reporting any glycaemic outcome, fasting glucose was reported in 173 (50.1%) and glycated hemoglobin (HbA1c) in 108 (31.3%), reflecting fasting and longer-term glycaemic exposure, respectively. Insulin (160, 46.4%) and C-peptide (14, 4.1%) provided measures of insulin response or secretion. Homeostatic model assessment indices (99, 28.7%) assessed insulin resistance or beta-cell function, and the quantitative insulin-sensitivity check index (17, 4.9%) assessed insulin sensitivity. Oral glucose tolerance or postprandial testing was reported in 74 (21.4%), glucose or insulin area-under-the-curve summaries in 48 (13.9%), and continuous glucose monitoring in 4 (1.2%). These assessment approaches and summary measures can overlap and address different physiological questions.

Outcome selection varied by duration. The 61 acute studies and 339 repeated-dosing studies both frequently reported glycaemic outcomes (88.5 and 82.6%), whereas lipid outcomes were reported in 16.4 and 72.3%, respectively. Among studies reporting glycaemic outcomes, postprandial or oral glucose tolerance measures appeared in 40 of 54 acute studies (74.1%) and 30 of 280 repeated-dosing studies (10.7%). Two studies combined acute and repeated dosing, and duration was unclear in 14. Domain coverage by duration and formulation is presented in Supplementary material S3.

Mapping outcome domains against the most-studied substances showed the concentration of reporting in the identified studies (Figure 4). Glycaemic and lipid outcomes were reported for nearly every well-studied substance, whereas other domains were substance-specific. Mulberry, for example, was studied almost exclusively for glycaemic outcomes (22 of 23 trials), and curcumin-bearing turmeric accounted for most reporting in the inflammatory and liver domains. Many substance-by-domain combinations were empty, identifying outcome domains not represented in the included literature that merit assessment in future focused searches.

**Figure 4:**
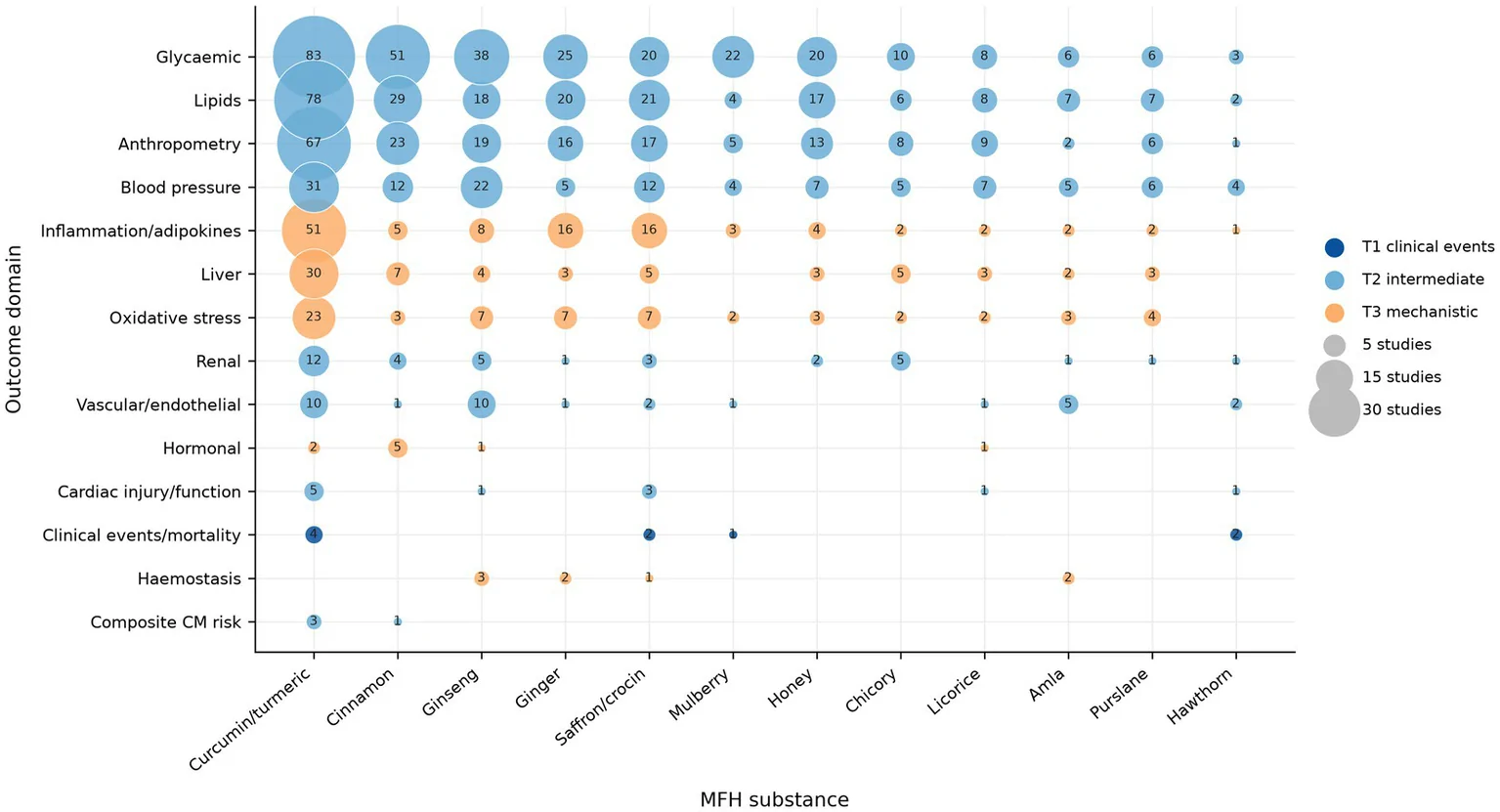
Evidence map of outcome domains for the most-studied substances. Bubble area is proportional to the number of studies reporting the corresponding domain. Colors indicate the outcome group (T1, T2, or T3).

### 3.3 Outcome groups and reporting practice

Classified by outcome group, the 2,231 records comprised 15 clinical event or mortality outcomes (T1, 0.7%), 1,610 intermediate cardiometabolic measures (T2, 72.2%), and 402 mechanistic biomarkers (T3, 18.0%); the remaining 204 (9.1%) concerned other outcomes or process measures. At the study level, 407 of 416 studies (97.8%) reported no clinical event or mortality outcome, and 202 (48.6%) reported at least one mechanistic biomarker; 9 studies (2.2%) reported a clinical event or mortality outcome.

Reporting-completeness signals diverged over time (Figure 2b). The proportion of trials reporting a registration number rose from 5% before 2010 to 45% in 2010 to 2014, 59% in 2015 to 2019, and 74% from 2020 onward. Over the same period the proportion of trials that explicitly designated a primary outcome declined, from 95% before 2010 to 87, 72, and 77% across the successive eras.

## 4 Discussion

This scoping review mapped the outcomes reported in 416 studies of MFH substances primarily addressing cardiometabolic health retrieved through four selected databases. Four features stand out. Reporting is concentrated in a small set of intermediate domains, principally glycaemic, lipid, anthropometric, and blood-pressure measures, while clinical event endpoints are almost entirely absent. Within those domains, lipid reporting commonly uses a conventional panel, while glycaemic measures reflect different constructs and study durations. Roughly one study in two reports at least one mechanistic biomarker, sometimes designated as a primary outcome. Finally, trial registration has climbed steeply over the period, yet explicit primary-outcome designation has not shown a corresponding increase.

The scarcity of clinical event endpoints needs to be read in context. Trials of foods and food-derived substances usually enrol healthy or early-risk participants, run for a few weeks to a few months, and are small (30); in this review almost four in five lasted no more than twelve weeks and the median sample was under sixty participants. Such studies have limited power to assess effects on clinical events. For something eaten as part of an ordinary diet, mounting a trial large and long enough to measure mortality or cardiovascular events is seldom proportionate and may not be feasible, though the large Mediterranean-diet prevention trials show it can occasionally be done (31). We therefore read the near-absence of hard endpoints as a structural feature of nutrition research, not as neglect. What matters more sits one level down, in which intermediate outcomes a trial chooses and how it measures them. Symptoms, daily functioning, quality of life, and adverse effects can also capture participant-relevant outcomes in shorter trials.

Consistency of measurement remains important to the usefulness of this evidence. Lipid reporting has largely converged on the conventional four-component panel, which facilitates comparison across trials. Glycaemic reporting spans fasting glucose, longer-term glycaemic exposure, postprandial responses, and insulin resistance or secretion. Their different roles explain part of the variation: acute studies emphasized postprandial responses, whereas repeated-dosing studies more often reported lipid and longer-term metabolic measures. The overall frequency of HbA1c should therefore be interpreted in relation to study duration and purpose. Standardization is most useful when trials address comparable populations and constructs, so that differences in definitions, measurement methods, and timepoints do not obstruct synthesis (14, 15, 17, 32).

More than a third of studies reported six or more outcome records, and among those that named a primary outcome, 50.3% named at least two. Co-primary designs and composite endpoints are legitimate when pre-specified, with decision rules and multiplicity control appropriate to the intended claim (33, 34). Post-hoc addition or promotion of outcomes raises a different concern. We did not extract multiplicity adjustments or routinely compare publications with protocols, so these practices cannot be distinguished from the number of primary outcomes alone. Future methodological reviews should examine these variables alongside registration and statistical analysis plans (35–37). Mechanistic markers add biological information, but their interpretation should remain tied to the study question and the clinical claim (20, 21).

Within the identified literature, the concentration of evidence on a few substances and the empty cells of the map help identify questions for focused reviews and outcome standardization. A small number of substances, led by curcumin-bearing turmeric, account for most of the evidence and most of the reporting in several domains, whereas almost half of the substances represented in the map are supported by no more than two studies each. Where substances have been studied, reporting often clusters narrowly, as in the near-exclusive glycaemic focus of mulberry trials, leaving adjacent domains unreported in the included studies. The map therefore distinguishes gaps in the identified literature, where no included study reported a domain for a substance, from areas of thin but present evidence, a distinction useful for prioritizing future focused searches (38).

Our findings extend prior work on outcome reporting in adjacent fields. A systematic review of TCM trials for hyperlipidaemia documented comparable variability in reported outcomes and motivated a condition-specific core outcome set (18, 19). The present review addresses MFH substances as a class across cardiometabolic conditions rather than a single condition, and it adds a distinction between clinical events, intermediate cardiometabolic measures, and mechanistic biomarkers. The findings support development of a minimum core outcome set for defined conditions and study purposes. Such a set should be developed through stakeholder consensus, including patients and clinicians (39).

Several implications follow for the conduct and appraisal of future trials. Selecting and clearly designating a small number of cardiometabolic outcomes relevant to the population studied (40, 41), defining and measuring them consistently (16), including a common core of measures for comparable glycaemic questions, registering them prospectively (42), and distinguishing clinical and intermediate outcomes from exploratory mechanistic markers (20) would each improve the comparability and interpretability of new evidence. For longer-term diabetes trials, HbA1c is a candidate core measure, alongside the conventional lipid panel when lipid effects are relevant. Acute studies need standardized challenges, sampling times, and response measures. Oxidative-stress and cytokine markers should generally serve as exploratory secondary outcomes in clinical efficacy trials, while retaining their role in studies designed to test mechanisms.

The food-based context also matters. The World Diet Initiative highlights the value of documenting heritage dietary diversity and studying its metabolic and host-microbiome interactions (43). The shift toward whole-food guidance similarly emphasizes the contribution of food matrices to cardiometabolic health (44). These perspectives support studying whole edible MFH materials in their dietary context and distinguishing their outcome profiles from those of extracts or isolated constituents.

This review has several strengths. It provides a map of outcome reporting in MFH cardiometabolic studies retrieved through four selected databases. We distinguished clinical events, intermediate measures, and mechanistic biomarkers. This approach identifies opportunities to improve comparability, particularly within glycaemic studies addressing similar constructs. By clarifying these issues, our findings offer an empirical baseline to improve the design, comparability, and overall quality of future trials. Several limitations temper the findings. First, the selected databases and search terms may have limited coverage of relevant studies, particularly those indexed only in Chinese regional databases (45, 46). Search sensitivity was not formally assessed, so empty cells in the map do not establish field-wide evidence gaps. Second, the Chinese catalogue-based boundary may affect which substances and research regions are represented and limits generalization to functional foods internationally. Excluding studies with non-cardiometabolic primary outcomes also limits the map to trials with a cardiometabolic focus. Third, domain and group assignments involve judgment; we provide the coding dictionary to make these decisions transparent. Finally, we characterized outcomes as reported in publications and did not routinely verify them against registered protocols (14, 35).

## 5 Conclusion

Among MFH cardiometabolic studies retrieved through the four selected databases, outcomes are concentrated in a few intermediate domains, with variation in measurement and primary-outcome designation. The scarcity of clinical event endpoints should be understood in relation to study purpose, population, and duration. Consistent definition, prospective registration, and clear primary-outcome reporting of a focused set of cardiometabolic outcomes would improve the usefulness of this evidence.

## Data Availability

The original contributions presented in the study are included in the article/Supplementary material, further inquiries can be directed to the corresponding author.

## Supplementary material

SUPPLEMENTARY TABLE S1 Study characteristics and outcome charting data.

## References

[1] Mensah GA Fuster V Murray CJL Roth GA. Global Burden of Cardiovascular Diseases and Risks Collaborators. Global burden of cardiovascular diseases and risks, 1990-2022. J Am Coll Cardiol. (1990) 82:2350–473. doi: 10.1016/j.jacc.2023.11.007, 38092509 PMC7615984

[2] Micha R Peñalvo JL Cudhea F Imamura F Rehm CD Mozaffarian D. Association between dietary factors and mortality from heart disease, stroke, and type 2 diabetes in the United States. JAMA. (2017) 317:912–24. doi: 10.1001/jama.2017.0947, 28267855 PMC5852674

[3] International Diabetes Federation. IDF Diabetes Atlas. 10th ed. Brussels: International Diabetes Federation (2021).

[4] Mozaffarian D. Dietary and policy priorities for cardiovascular disease, diabetes, and obesity: a comprehensive review. Circulation. (2016) 133:187–225. doi: 10.1161/CIRCULATIONAHA.115.018585, 26746178 PMC4814348

[5] Fei S Rao Y Lai J Zhang B Ma H Ma X . Medicine-food homology substances in cardiovascular disease prevention and management: from mechanisms to clinical evidence and future translation. Front Nutr. (2026) 13:1825606. doi: 10.3389/fnut.2026.1825606, 42428610 PMC13345857

[6] Liu J Lee PK Wu W Huang J Zhao D. Medicine food homology materials for promoting resilience against metabolic syndrome: recent technology advances and challenges. Trends Food Sci Technol. (2025) 164:105236. doi: 10.1016/j.tifs.2025.105236

[7] Hou Y Jiang JG. Origin and concept of medicine food homology and its application in modern functional foods. Food Funct. (2013) 4:1727–41. doi: 10.1039/c3fo60295h, 24100549

[8] Qin S Huang L Gong J Shen S Huang J Ren H . Efficacy and safety of turmeric and curcumin in lowering blood lipid levels in patients with cardiovascular risk factors: a meta-analysis of randomized controlled trials. Nutr J. (2017) 16:68. doi: 10.1186/s12937-017-0293-y, 29020971 PMC5637251

[9] Allen RW Schwartzman E Baker WL Coleman CI Phung OJ. Cinnamon use in type 2 diabetes: an updated systematic review and meta-analysis. Ann Fam Med. (2013) 11:452–9. doi: 10.1370/afm.1517, 24019277 PMC3767714

[10] Shishtar E Sievenpiper JL Djedovic V Cozma AI Ha V Jayalath VH . The effect of ginseng (the genus *Panax*) on glycemic control: a systematic review and meta-analysis of randomized controlled clinical trials. PLoS One. (2014) 9:e107391. doi: 10.1371/journal.pone.0107391, 25265315 PMC4180277

[11] Daily JW Yang M Kim DS Park S. Efficacy of ginger for treating type 2 diabetes: a systematic review and meta-analysis of randomized clinical trials. J Ethn Foods. (2015) 2:36–43. doi: 10.1016/j.jef.2015.02.007

[12] Chalmers I Glasziou P. Avoidable waste in the production and reporting of research evidence. Lancet. (2009) 374:86–9. doi: 10.1016/S0140-6736(09)60329-9, 19525005

[13] Glasziou P Altman DG Bossuyt P Boutron I Clarke M Julious S . Reducing waste from incomplete or unusable reports of biomedical research. Lancet. (2014) 383:267–76. doi: 10.1016/S0140-6736(13)62228-X, 24411647

[14] Chan AW Hróbjartsson A Haahr MT Gøtzsche PC Altman DG. Empirical evidence for selective reporting of outcomes in randomized trials: comparison of protocols to published articles. JAMA. (2004) 291:2457–65. doi: 10.1001/jama.291.20.2457, 15161896

[15] Dwan K Altman DG Arnaiz JA Bloom J Chan AW Cronin E . Systematic review of the empirical evidence of study publication bias and outcome reporting bias. PLoS One. (2008) 3:e3081. doi: 10.1371/journal.pone.0003081, 18769481 PMC2518111

[16] Williamson PR Altman DG Bagley H Barnes KL Blazeby JM Brookes ST . The COMET handbook: version 1.0. Trials. (2017) 18:280. doi: 10.1186/s13063-017-1978-4, 28681707 PMC5499094

[17] Kirkham JJ Dwan KM Altman DG Gamble C Dodd S Smyth R . The impact of outcome reporting bias in randomised controlled trials on a cohort of systematic reviews. BMJ. (2010) 340:c365. doi: 10.1136/bmj.c365, 20156912

[18] Li G Han R Cao W Wen Z Chen X. Outcome reporting variability in trials of Chinese medicine for hyperlipidemia: a systematic review for developing a core outcome set. Evid Based Complement Alternat Med. (2021) 2021:1–9. doi: 10.1155/2021/8822215, 34211576 PMC8211496

[19] Li G Han R Lin M Wen Z Chen X. Developing a core outcome set for clinical trials of Chinese medicine for hyperlipidemia. Front Pharmacol. (2022) 13:847101. doi: 10.3389/fphar.2022.847101, 35586053 PMC9108338

[20] Prentice RL. Surrogate endpoints in clinical trials: definition and operational criteria. Stat Med. (1989) 8:431–40. doi: 10.1002/sim.4780080407, 2727467

[21] Fleming TR DeMets DL. Surrogate end points in clinical trials: are we being misled? Ann Intern Med. (1996) 125:605–13. doi: 10.7326/0003-4819-125-7-199610010-00011, 8815760

[22] Zheng Q Zhou Z Li T Xu J Zhou Y Cui Y . Charting the evolution of personalised nutrition: a comprehensive bibliometric survey of global research landscapes and future trajectories. Nutr Res Rev. (2026) 39:e17. doi: 10.1017/S0954422426100389, 41883152

[23] Peters MDJ Marnie C Tricco AC Pollock D Munn Z Alexander L . Updated methodological guidance for the conduct of scoping reviews. JBI Evid Synth. (2020) 18:2119–26. doi: 10.11124/jbies-20-00167, 33038124

[24] Arksey H O'Malley L. Scoping studies: towards a methodological framework. Int J Soc Res Methodol. (2005) 8:19–32. doi: 10.1080/1364557032000119616

[25] Levac D Colquhoun H O'Brien KK. Scoping studies: advancing the methodology. Implement Sci. (2010) 5:69. doi: 10.1186/1748-5908-5-69, 20854677 PMC2954944

[26] Tricco AC Lillie E Zarin W O'Brien KK Colquhoun H Levac D . PRISMA extension for scoping reviews (PRISMA-ScR): checklist and explanation. Ann Intern Med. (2018) 169:467–73. doi: 10.7326/M18-0850, 30178033

[27] Zheng Q Xu J Gao Y Liu M Cheng L Xiong L . Past, present and future of living systematic review: a bibliometrics analysis. BMJ Glob Health. (2022) 7:e009378. doi: 10.1136/bmjgh-2022-009378, 36220305 PMC9558789

[28] Higgins JPT Thomas J Chandler J Cumpston M Li T Page MJ , editors. Cochrane Handbook for Systematic Reviews of Interventions. 2nd ed. Chichester: John Wiley & Sons (2019). doi: 10.1002/9781119536604

[29] Aromataris E Munn Z, editors. JBI Manual for Evidence Synthesis. Adelaide: JBI (2020).

[30] Trepanowski JF Ioannidis JPA. Perspective: limiting dependence on nonrandomized studies and improving randomized trials in human nutrition research: why and how. Adv Nutr. (2018) 9:367–77. doi: 10.1093/advances/nmy014, 30032218 PMC6054237

[31] Estruch R Ros E Salas-Salvadó J Covas MI Corella D Arós F . Primary prevention of cardiovascular disease with a Mediterranean diet supplemented with extra-virgin olive oil or nuts. N Engl J Med. (2018) 378:e34. doi: 10.1056/NEJMoa1800389, 29897866

[32] Boutron I Dutton S Ravaud P Altman DG. Reporting and interpretation of randomized controlled trials with statistically nonsignificant results for primary outcomes. JAMA. (2010) 303:2058–64. doi: 10.1001/jama.2010.651 20501928

[33] Vickerstaff V Omar RZ Ambler G. Methods to adjust for multiple comparisons in the analysis and sample size calculation of randomised controlled trials with multiple primary outcomes. BMC Med Res Methodol. (2019) 19:129. doi: 10.1186/s12874-019-0754-4, 31226934 PMC6588937

[34] US Food and Drug Administration. Multiple Endpoints in Clinical Trials: Guidance for Industry. Silver Spring, MD: FDA (2022).

[35] Mathieu S Boutron I Moher D Altman DG Ravaud P. Comparison of registered and published primary outcomes in randomized controlled trials. JAMA. (2009) 302:977–84. doi: 10.1001/jama.2009.1242, 19724045

[36] Goldacre B Drysdale H Dale A Milosevic I Slade E Hartley P . COMPare: a prospective cohort study correcting and monitoring 58 misreported trials in real time. Trials. (2019) 20:118. doi: 10.1186/s13063-019-3173-2, 30760329 PMC6375128

[37] Zarin DA Tse T Williams RJ Rajakannan T. Update on trial registration 11 years after the ICMJE policy was established. N Engl J Med. (2017) 376:383–91. doi: 10.1056/NEJMsr1601330, 28121511 PMC5813248

[38] Saran A White H. Evidence and gap maps: a comparison of different approaches. Campbell Syst Rev. (2018) 14:1–38. doi: 10.4073/cmdp.2018.2, 37131398 PMC8428058

[39] Gargon E Gurung B Medley N Altman DG Blazeby JM Clarke M . Choosing important health outcomes for comparative effectiveness research: a systematic review. PLoS One. (2014) 9:e99111. doi: 10.1371/journal.pone.0099111, 24932522 PMC4059640

[40] Schulz KF Altman DG Moher D, for the CONSORT Group. CONSORT 2010 statement: updated guidelines for reporting parallel group randomised trials. BMJ. (2010) 340:c332. doi: 10.1136/bmj.c332, 20332509 PMC2844940

[41] Chan AW Tetzlaff JM Altman DG Laupacis A Gøtzsche PC Krleža-Jerić K . SPIRIT 2013 statement: defining standard protocol items for clinical trials. Ann Intern Med. (2013) 158:200–7. doi: 10.7326/0003-4819-158-3-201302050-00583, 23295957 PMC5114123

[42] De Angelis C Drazen JM Frizelle FA Haug C Hoey J Horton R . Clinical trial registration: a statement from the International Committee of Medical Journal Editors. N Engl J Med. (2004) 351:1250–1. doi: 10.1056/NEJMe048225, 15356289

[43] de Mast Q Brown CV Mwakasungura F Nakanjako D Temba GS Kumar V . Studying vanishing dietary diversity before it is lost: the world diet initiative. Nat Med. (2026) 32:3120–3. doi: 10.1038/s41591-026-04520-5, 42458009

[44] Astrup A Abodi M Mazzocchi A Agostoni C Brenna JT Krauss RM . Foods to prevent Cardiometabolic disease: moving from saturated fat to whole foods. Curr Dev Nutr. (2026) 10:107717. doi: 10.1016/j.cdnut.2026.107717, 42305911 PMC13267541

[45] Xia J Wright J Adams CE. Five large Chinese biomedical bibliographic databases: accessibility and coverage. Health Inf Libr J. (2008) 25:55–61. doi: 10.1111/j.1471-1842.2007.00734.x, 18251914

[46] Morrison A Polisena J Husereau D Moulton K Clark M Fiander M . The effect of English-language restriction on systematic review-based meta-analyses: a systematic review of empirical studies. Int J Technol Assess Health Care. (2012) 28:138–44. doi: 10.1017/S0266462312000086, 22559755

