# Supplementary material for: Outcome reporting in randomized trials of medicine–food homology interventions for cardiometabolic disease: a scoping review
Source: Front Nutr. 2026 Oct 12;13:1937261. doi: 10.3389/fnut.2026.1937261 (PMC13696507; doi:10.3389/fnut.2026.1937261)
Supplement: Supplementary file 1 [file Supplementary_file_1.DOCX]

**Supplementary figure 1.** Physical map between the prion protein gene (*PRNP*) and *prion-like* protein gene (*PRND*) genes among several species, humans, cattle, sheep, goats, horses, dogs, rabbits, and bats. SP (coral boxes), signal peptide; OrR (yellow boxes), octapeptide repeat region; HD (red boxes), hydrophobic domain.
**Supplementary table 1.** Sample sizes and polymorphisms distributions by bat species.

| Polymorphisms | Species | Genotype frequencies, *n* | | |
| --- | --- | --- | --- | --- |
|  |  | M/M | M/m | m/m |
| c.214G>A | Greater horseshoe bat, *Rhinolophus ferrumequinum* | 58 |  |  |
|  | Common bent-wing bat, *Miniopterus schreibersi* | 13 |  |  |
|  | Jpanese pipistrelle, *Pipistrellus abramus* | 9 |  |  |
|  | Steppe Whiskered Bat, *Myotis aurascens* | 4 |  |  |
|  | Alashanian pipistrelle, *Hypsugo alaschanicus* | 3 |  |  |
|  | Eastern water bat, *Myotis petax* | 3 |  |  |
|  | Greater tube-nosed bat, *Murina leucogaster* | 2 |  |  |
|  | Common serotine bat, *Eptesicus serotinus* | 2 |  |  |
|  | Large-footed Myotis, *Myotis adversus* | 2 |  |  |
|  | Asian parti-colored bat, *Vespertilio sinensis* |  |  | 1 |
|  | Big-footed myotis, *Myotis macrodactylus* | 1 |  |  |
| c.216C>T | Greater horseshoe bat, *Rhinolophus ferrumequinum* | 50 | 8 |  |
|  | Common bent-wing bat, *Miniopterus schreibersi* | 10 | 3 |  |
|  | Jpanese pipistrelle, *Pipistrellus abramus* | 9 |  |  |
|  | Steppe Whiskered Bat, *Myotis aurascens* | 4 |  |  |
|  | Alashanian pipistrelle, *Hypsugo alaschanicus* | 3 |  |  |
|  | Eastern water bat, *Myotis petax* | 3 |  |  |
|  | Greater tube-nosed bat, *Murina leucogaster* | 2 |  |  |
|  | Common serotine bat, *Eptesicus serotinus* | 2 |  |  |
|  | Large-footed Myotis, *Myotis adversus* | 2 |  |  |
|  | Asian parti-colored bat, *Vespertilio sinensis* | 1 |  |  |
|  | Big-footed myotis, *Myotis macrodactylus* | 1 |  |  |
| c.391A>C | Greater horseshoe bat, *Rhinolophus ferrumequinum* | 55 | 3 |  |
|  | Common bent-wing bat, *Miniopterus schreibersi* | 13 |  |  |
|  | Jpanese pipistrelle, *Pipistrellus abramus* | 8 | 1 |  |
|  | Steppe Whiskered Bat, *Myotis aurascens* | 1 | 3 |  |
|  | Alashanian pipistrelle, *Hypsugo alaschanicus* | 2 | 1 |  |
|  | Eastern water bat, *Myotis petax* | 2 | 1 |  |
|  | Greater tube-nosed bat, *Murina leucogaster* | 2 |  |  |
|  | Common serotine bat, *Eptesicus serotinus* | 2 |  |  |
|  | Large-footed Myotis, *Myotis adversus* | 2 |  |  |
|  | Asian parti-colored bat, *Vespertilio sinensis* | 1 |  |  |
|  | Big-footed myotis, *Myotis macrodactylus* | 1 |  |  |
| c.392G>C | Greater horseshoe bat, *Rhinolophus ferrumequinum* | 48 | 9 | 1 |
|  | Common bent-wing bat, *Miniopterus schreibersi* | 11 | 2 |  |
|  | Jpanese pipistrelle, *Pipistrellus abramus* | 8 | 1 |  |
|  | Steppe Whiskered Bat, *Myotis aurascens* | 4 |  |  |
|  | Alashanian pipistrelle, *Hypsugo alaschanicus* | 3 |  |  |
|  | Eastern water bat, *Myotis petax* | 3 |  |  |
|  | Greater tube-nosed bat, *Murina leucogaster* | 2 |  |  |
|  | Common serotine bat, *Eptesicus serotinus* |  | 2 |  |
|  | Large-footed Myotis, *Myotis adversus* |  | 2 |  |
|  | Asian parti-colored bat, *Vespertilio sinensis* | 1 |  |  |
|  | Big-footed myotis, *Myotis macrodactylus* | 1 |  |  |
| c.456G>A | Greater horseshoe bat, *Rhinolophus ferrumequinum* | 56 | 2 |  |
|  | Common bent-wing bat, *Miniopterus schreibersi* | 12 | 1 |  |
|  | Jpanese pipistrelle, *Pipistrellus abramus* | 9 |  |  |
|  | Steppe Whiskered Bat, *Myotis aurascens* | 4 |  |  |
|  | Alashanian pipistrelle, *Hypsugo alaschanicus* | 3 |  |  |
|  | Eastern water bat, *Myotis petax* | 3 |  |  |
|  | Greater tube-nosed bat, *Murina leucogaster* | 2 |  |  |
|  | Common serotine bat, *Eptesicus serotinus* | 2 |  |  |
|  | Large-footed Myotis, *Myotis adversus* | 1 | 1 |  |
|  | Asian parti-colored bat, *Vespertilio sinensis* | 1 |  |  |
|  | Big-footed myotis, *Myotis macrodactylus* | 1 |  |  |
| c.465A>G | Greater horseshoe bat, *Rhinolophus ferrumequinum* | 58 |  |  |
|  | Common bent-wing bat, *Miniopterus schreibersi* | 13 |  |  |
|  | Jpanese pipistrelle, *Pipistrellus abramus* | 8 | 1 |  |
|  | Steppe Whiskered Bat, *Myotis aurascens* | 4 |  |  |
|  | Alashanian pipistrelle, *Hypsugo alaschanicus* | 3 |  |  |
|  | Eastern water bat, *Myotis petax* | 3 |  |  |
|  | Greater tube-nosed bat, *Murina leucogaster* | 2 |  |  |
|  | Common serotine bat, *Eptesicus serotinus* | 2 |  |  |
|  | Large-footed Myotis, *Myotis adversus* | 2 |  |  |
|  | Asian parti-colored bat, *Vespertilio sinensis* | 1 |  |  |
|  | Big-footed myotis, *Myotis macrodactylus* | 1 |  |  |
| c.478A>G | Greater horseshoe bat, *Rhinolophus ferrumequinum* | 58 |  |  |
|  | Common bent-wing bat, *Miniopterus schreibersi* | 13 |  |  |
|  | Jpanese pipistrelle, *Pipistrellus abramus* | 8 | 1 |  |
|  | Steppe Whiskered Bat, *Myotis aurascens* | 4 |  |  |
|  | Alashanian pipistrelle, *Hypsugo alaschanicus* | 3 |  |  |
|  | Eastern water bat, *Myotis petax* | 3 |  |  |
|  | Greater tube-nosed bat, *Murina leucogaster* | 2 |  |  |
|  | Common serotine bat, *Eptesicus serotinus* | 2 |  |  |
|  | Large-footed Myotis, *Myotis adversus* | 2 |  |  |
|  | Asian parti-colored bat, *Vespertilio sinensis* | 1 |  |  |
|  | Big-footed myotis, *Myotis macrodactylus* | 1 |  |  |
