# Supplementary material for: Outcome reporting in randomized trials of medicine–food homology interventions for cardiometabolic disease: a scoping review
Source: Front Nutr. 2026 Oct 12;13:1937261. doi: 10.3389/fnut.2026.1937261 (PMC13696507; doi:10.3389/fnut.2026.1937261)
Supplement: Supplementary file 2 [file Supplementary_file_2.DOCX]

**Supplementary Material**

Outcome Reporting in Randomized Trials of Medicine-Food Homology Interventions for Cardiometabolic Disease: A Scoping Review.

*Contents: S1 Search strategies and substance list; S2 Outcome-domain coding; S3 Included studies and stratified summaries; S4 Full-text exclusions.*

**S1. Database search strategies and MFH substance list**

**PubMed (MEDLINE)**

*Search set 1:*

"clove"[tiab] OR Syzygium[tiab] OR "star anise"[tiab] OR Illicium[tiab] OR "sword bean"[tiab] OR Canavalia[tiab] OR fennel[tiab] OR Foeniculum[tiab] OR Cirsium[tiab] OR "Chinese yam"[tiab] OR Dioscorea[tiab] OR hawthorn[tiab] OR Crataegus[tiab] OR purslane[tiab] OR Portulaca[tiab] OR Chaenomeles[tiab] OR "flowering quince"[tiab] OR "hemp seed"[tiab] OR "Cannabis sativa"[tiab] OR "bitter orange"[tiab] OR "Citrus aurantium"[tiab] OR Polygonatum[tiab] OR licorice[tiab] OR liquorice[tiab] OR Glycyrrhiza[tiab] OR "Angelica dahurica"[tiab] OR ginkgo[tiab] OR "hyacinth bean"[tiab] OR Lablab[tiab] OR Dolichos[tiab] OR longan[tiab] OR Dimocarpus[tiab] OR "cassia seed"[tiab] OR "Senna obtusifolia"[tiab] OR "lily bulb"[tiab] OR Lilium[tiab] OR nutmeg[tiab] OR Myristica[tiab] OR cinnamon[tiab] OR Cinnamomum[tiab] OR emblica[tiab] OR "Phyllanthus emblica"[tiab] OR "Indian gooseberry"[tiab] OR "finger citron"[tiab] OR "Citrus medica"[tiab] OR "apricot kernel"[tiab] OR "sea buckthorn"[tiab] OR Hippophae[tiab] OR oyster[tiab] OR Ostrea[tiab] OR "gorgon fruit"[tiab] OR Euryale[tiab] OR "Sichuan pepper"[tiab] OR Zanthoxylum[tiab] OR "adzuki bean"[tiab] OR "rice bean"[tiab] OR Vigna[tiab] OR "donkey-hide gelatin"[tiab] OR malt[tiab] OR Hordeum[tiab] OR kelp[tiab] OR Laminaria[tiab] OR jujube[tiab] OR Ziziphus[tiab] OR "monk fruit"[tiab] OR Siraitia[tiab] OR honeysuckle[tiab] OR Lonicera[tiab] OR "Chinese olive"[tiab] OR Canarium[tiab] OR Houttuynia[tiab] OR ginger[tiab] OR Zingiber[tiab] OR gingerol[tiab] OR Hovenia[tiab] OR goji[tiab] OR wolfberry[tiab] OR Lycium[tiab] OR gardenia[tiab] OR Gardenia[tiab] OR "Amomum villosum"[tiab] OR Scaphium[tiab] OR Sterculia[tiab] OR poria[tiab] OR Wolfiporia[tiab] OR citron[tiab] OR Mosla[tiab] OR Elsholtzia[tiab] OR "peach kernel"[tiab] OR mulberry[tiab] OR Morus[tiab] OR Platycodon[tiab] OR "Alpinia oxyphylla"[tiab] OR "lotus leaf"[tiab] OR "lotus seed"[tiab] OR Nelumbo[tiab] OR "radish seed"[tiab] OR Raphanus[tiab] OR "Alpinia officinarum"[tiab] OR Lophatherum[tiab] OR "fermented soybean"[tiab] OR douchi[tiab] OR chrysanthemum[tiab] OR Chrysanthemum[tiab] OR chicory[tiab] OR Cichorium[tiab] OR "mustard seed"[tiab] OR Sinapis[tiab] OR "Polygonatum sibiricum"[tiab] OR perilla[tiab] OR Perilla[tiab] OR kudzu[tiab] OR Pueraria[tiab] OR puerarin[tiab] OR "black sesame"[tiab] OR Sesamum[tiab] OR "black pepper"[tiab] OR "Piper nigrum"[tiab] OR piperine[tiab] OR Sophora[tiab] OR Styphnolobium[tiab] OR dandelion[tiab] OR Taraxacum[tiab] OR honey[tiab] OR Torreya[tiab] OR "spiny date seed"[tiab] OR Imperata[tiab] OR Phragmites[tiab] OR "tangerine peel"[tiab] OR "citrus peel"[tiab] OR "Citrus reticulata"[tiab] OR hesperidin[tiab] OR peppermint[tiab] OR Mentha[tiab] OR "coix seed"[tiab] OR "Job's tears"[tiab] OR Coix[tiab] OR "Allium macrostemon"[tiab] OR "Allium chinense"[tiab] OR raspberry[tiab] OR Rubus[tiab] OR Agastache[tiab] OR Pogostemon[tiab] OR "Angelica sinensis"[tiab] OR "dong quai"[tiab] OR Kaempferia[tiab] OR saffron[tiab] OR Crocus[tiab] OR crocin[tiab] OR tsaoko[tiab] OR "Amomum tsao-ko"[tiab] OR turmeric[tiab] OR Curcuma[tiab] OR curcumin[tiab] OR "long pepper"[tiab] OR "Piper longum"[tiab] OR Codonopsis[tiab] OR Cistanche[tiab] OR Dendrobium[tiab] OR "American ginseng"[tiab] OR "Panax quinquefolius"[tiab] OR astragalus[tiab] OR Astragalus[tiab] OR astragaloside[tiab] OR Ganoderma[tiab] OR reishi[tiab] OR lingzhi[tiab] OR "Cornus officinalis"[tiab] OR Gastrodia[tiab] OR gastrodin[tiab] OR Eucommia[tiab] OR ginseng[tiab] OR "Panax ginseng"[tiab] OR ginsenoside[tiab] OR "Lonicera macranthoides"[tiab] OR coriander[tiab] OR cilantro[tiab] OR Coriandrum[tiab] OR "Rosa rugosa"[tiab] OR "pine pollen"[tiab] OR "Pueraria thomsonii"[tiab] OR "Microcos paniculata"[tiab] OR selfheal[tiab] OR "self-heal"[tiab] OR Prunella[tiab]

*Search set 2:*

cardiovascular[tiab] OR cardiometabolic[tiab] OR cardiac[tiab] OR coronary[tiab] OR "heart disease"[tiab] OR hypertension[tiab] OR hypertensive[tiab] OR "blood pressure"[tiab] OR dyslipidemia[tiab] OR dyslipidaemia[tiab] OR hyperlipidemia[tiab] OR hyperlipidaemia[tiab] OR hypercholesterolemia[tiab] OR hypercholesterolaemia[tiab] OR cholesterol[tiab] OR lipid[tiab] OR lipids[tiab] OR triglyceride*[tiab] OR LDL[tiab] OR HDL[tiab] OR lipoprotein[tiab] OR diabet*[tiab] OR prediabet*[tiab] OR "impaired glucose"[tiab] OR "insulin resistance"[tiab] OR insulin[tiab] OR glycemic[tiab] OR glycaemic[tiab] OR glucose[tiab] OR HbA1c[tiab] OR hyperglycemia[tiab] OR hyperglycaemia[tiab] OR "metabolic syndrome"[tiab] OR obesity[tiab] OR obese[tiab] OR overweight[tiab] OR adiposity[tiab] OR atheroscleros*[tiab] OR "endothelial function"[tiab] OR "arterial stiffness"[tiab] OR "intima-media"[tiab] OR "cardiovascular diseases"[mh] OR "metabolic syndrome"[mh] OR "diabetes mellitus, type 2"[mh] OR "hypertension"[mh] OR "dyslipidemias"[mh] OR "obesity"[mh]

*Search set 3:*

"randomized controlled trial"[pt] OR "controlled clinical trial"[pt] OR randomized[tiab] OR randomised[tiab] OR placebo[tiab] OR "clinical trials as topic"[mh:noexp] OR randomly[tiab] OR trial[ti]

*Search set 4:*

#4	#1 AND #2 AND #3

#5	#4 NOT (animals[mh] NOT humans[mh])

**Embase (Ovid)**

*Search set 1:*

(clove or Syzygium or "star anise" or Illicium or "sword bean" or Canavalia or fennel or Foeniculum or Cirsium or "Chinese yam" or Dioscorea or hawthorn or Crataegus or purslane or Portulaca or Chaenomeles or "flowering quince" or "hemp seed" or "Cannabis sativa" or "bitter orange" or "Citrus aurantium" or Polygonatum or licorice or liquorice or Glycyrrhiza or "Angelica dahurica" or ginkgo or "hyacinth bean" or Lablab or Dolichos or longan or Dimocarpus or "cassia seed" or "Senna obtusifolia" or "lily bulb" or Lilium or nutmeg or Myristica or cinnamon or Cinnamomum or emblica or "Phyllanthus emblica" or "Indian gooseberry" or "finger citron" or "Citrus medica" or "apricot kernel" or "sea buckthorn" or Hippophae or oyster or Ostrea or "gorgon fruit" or Euryale or "Sichuan pepper" or Zanthoxylum or "adzuki bean" or "rice bean" or Vigna or "donkey-hide gelatin" or malt or Hordeum or kelp or Laminaria or jujube or Ziziphus or "monk fruit" or Siraitia or honeysuckle or Lonicera or "Chinese olive" or Canarium or Houttuynia or ginger or Zingiber or gingerol or Hovenia or goji or wolfberry or Lycium or gardenia or Gardenia or "Amomum villosum" or Scaphium or Sterculia or poria or Wolfiporia or citron or Mosla or Elsholtzia or "peach kernel" or mulberry or Morus or Platycodon or "Alpinia oxyphylla" or "lotus leaf" or "lotus seed" or Nelumbo or "radish seed" or Raphanus or "Alpinia officinarum" or Lophatherum or "fermented soybean" or douchi or chrysanthemum or Chrysanthemum or chicory or Cichorium or "mustard seed" or Sinapis or "Polygonatum sibiricum" or perilla or Perilla or kudzu or Pueraria or puerarin or "black sesame" or Sesamum or "black pepper" or "Piper nigrum" or piperine or Sophora or Styphnolobium or dandelion or Taraxacum or honey or Torreya or "spiny date seed" or Imperata or Phragmites or "tangerine peel" or "citrus peel" or "Citrus reticulata" or hesperidin or peppermint or Mentha or "coix seed" or "Job's tears" or Coix or "Allium macrostemon" or "Allium chinense" or raspberry or Rubus or Agastache or Pogostemon or "Angelica sinensis" or "dong quai" or Kaempferia or saffron or Crocus or crocin or tsaoko or "Amomum tsao-ko" or turmeric or Curcuma or curcumin or "long pepper" or "Piper longum" or Codonopsis or Cistanche or Dendrobium or "American ginseng" or "Panax quinquefolius" or astragalus or Astragalus or astragaloside or Ganoderma or reishi or lingzhi or "Cornus officinalis" or Gastrodia or gastrodin or Eucommia or ginseng or "Panax ginseng" or ginsenoside or "Lonicera macranthoides" or coriander or cilantro or Coriandrum or "Rosa rugosa" or "pine pollen" or "Pueraria thomsonii" or "Microcos paniculata" or selfheal or "self-heal" or Prunella).ti,ab,kw.

*Search set 2:*

(cardiovascular or cardiometabolic or cardiac or coronary or "heart disease" or hypertension or hypertensive or "blood pressure" or dyslipidemia or dyslipidaemia or hyperlipidemia or hyperlipidaemia or hypercholesterolemia or hypercholesterolaemia or cholesterol or lipid or lipids or triglyceride* or LDL or HDL or lipoprotein or diabet* or prediabet* or "impaired glucose" or "insulin resistance" or insulin or glycemic or glycaemic or glucose or HbA1c or hyperglycemia or hyperglycaemia or "metabolic syndrome" or obesity or obese or overweight or adiposity or atheroscleros* or "endothelial function" or "arterial stiffness" or "intima-media").ti,ab,kw.

*Search set 3:*

(randomized controlled trial/ or controlled clinical trial/ or randomi#ed.ti,ab. or randomly.ti,ab. or placebo.ti,ab. or trial.ti. or "double blind*".ti,ab. or crossover.ti,ab.)

*Search set 4:*

4	1 and 2 and 3

5	4 not ((exp animal/ or nonhuman/) not exp human/)

**Web of Science Core Collection**

*Search set 1:*

TS=("clove" OR Syzygium OR "star anise" OR Illicium OR "sword bean" OR Canavalia OR fennel OR Foeniculum OR Cirsium OR "Chinese yam" OR Dioscorea OR hawthorn OR Crataegus OR purslane OR Portulaca OR Chaenomeles OR "flowering quince" OR "hemp seed" OR "Cannabis sativa" OR "bitter orange" OR "Citrus aurantium" OR Polygonatum OR licorice OR liquorice OR Glycyrrhiza OR "Angelica dahurica" OR ginkgo OR "hyacinth bean" OR Lablab OR Dolichos OR longan OR Dimocarpus OR "cassia seed" OR "Senna obtusifolia" OR "lily bulb" OR Lilium OR nutmeg OR Myristica OR cinnamon OR Cinnamomum OR emblica OR "Phyllanthus emblica" OR "Indian gooseberry" OR "finger citron" OR "Citrus medica" OR "apricot kernel" OR "sea buckthorn" OR Hippophae OR oyster OR Ostrea OR "gorgon fruit" OR Euryale OR "Sichuan pepper" OR Zanthoxylum OR "adzuki bean" OR "rice bean" OR Vigna OR "donkey-hide gelatin" OR malt OR Hordeum OR kelp OR Laminaria OR jujube OR Ziziphus OR "monk fruit" OR Siraitia OR honeysuckle OR Lonicera OR "Chinese olive" OR Canarium OR Houttuynia OR ginger OR Zingiber OR gingerol OR Hovenia OR goji OR wolfberry OR Lycium OR gardenia OR Gardenia OR "Amomum villosum" OR Scaphium OR Sterculia OR poria OR Wolfiporia OR citron OR Mosla OR Elsholtzia OR "peach kernel" OR mulberry OR Morus OR Platycodon OR "Alpinia oxyphylla" OR "lotus leaf" OR "lotus seed" OR Nelumbo OR "radish seed" OR Raphanus OR "Alpinia officinarum" OR Lophatherum OR "fermented soybean" OR douchi OR chrysanthemum OR Chrysanthemum OR chicory OR Cichorium OR "mustard seed" OR Sinapis OR "Polygonatum sibiricum" OR perilla OR Perilla OR kudzu OR Pueraria OR puerarin OR "black sesame" OR Sesamum OR "black pepper" OR "Piper nigrum" OR piperine OR Sophora OR Styphnolobium OR dandelion OR Taraxacum OR honey OR Torreya OR "spiny date seed" OR Imperata OR Phragmites OR "tangerine peel" OR "citrus peel" OR "Citrus reticulata" OR hesperidin OR peppermint OR Mentha OR "coix seed" OR "Job's tears" OR Coix OR "Allium macrostemon" OR "Allium chinense" OR raspberry OR Rubus OR Agastache OR Pogostemon OR "Angelica sinensis" OR "dong quai" OR Kaempferia OR saffron OR Crocus OR crocin OR tsaoko OR "Amomum tsao-ko" OR turmeric OR Curcuma OR curcumin OR "long pepper" OR "Piper longum" OR Codonopsis OR Cistanche OR Dendrobium OR "American ginseng" OR "Panax quinquefolius" OR astragalus OR astragaloside OR Ganoderma OR reishi OR lingzhi OR "Cornus officinalis" OR Gastrodia OR gastrodin OR Eucommia OR ginseng OR "Panax ginseng" OR ginsenoside OR "Lonicera macranthoides" OR coriander OR cilantro OR Coriandrum OR "Rosa rugosa" OR "pine pollen" OR "Pueraria thomsonii" OR "Microcos paniculata" OR selfheal OR "self-heal" OR Prunella)

*Search set 2:*

TS=(cardiovascular OR cardiometabolic OR cardiac OR coronary OR "heart disease" OR hypertension OR hypertensive OR "blood pressure" OR dyslipidemia OR dyslipidaemia OR hyperlipidemia OR hyperlipidaemia OR hypercholesterolemia OR hypercholesterolaemia OR cholesterol OR lipid OR lipids OR triglyceride* OR LDL OR HDL OR lipoprotein OR diabet* OR prediabet* OR "insulin resistance" OR insulin OR glycemic OR glycaemic OR glucose OR HbA1c OR "metabolic syndrome" OR obesity OR obese OR overweight OR adiposity OR atheroscleros* OR "endothelial function" OR "arterial stiffness" OR "intima-media")

*Search set 3:*

TS=(randomized OR randomised OR "randomly allocated" OR placebo OR "controlled trial" OR "randomized controlled trial" OR "double blind*" OR "single blind*" OR crossover)

*Search set 4:*

#4	#1 AND #2 AND #3

*Search set 5:*

#5	#4 NOT TS=(rat OR rats OR mouse OR mice OR murine OR rabbit* OR porcine OR canine OR zebrafish OR "in vitro" OR "cell line")

**Cochrane CENTRAL**

*Search set 1:*

(clove OR Syzygium OR "star anise" OR Illicium OR "sword bean" OR Canavalia OR fennel OR Foeniculum OR Cirsium OR "Chinese yam" OR Dioscorea OR hawthorn OR Crataegus OR purslane OR Portulaca OR Chaenomeles OR "flowering quince" OR "hemp seed" OR "Cannabis sativa" OR "bitter orange" OR "Citrus aurantium" OR Polygonatum OR licorice OR liquorice OR Glycyrrhiza OR "Angelica dahurica" OR ginkgo OR "hyacinth bean" OR Lablab OR Dolichos OR longan OR Dimocarpus OR "cassia seed" OR "Senna obtusifolia" OR "lily bulb" OR Lilium OR nutmeg OR Myristica OR cinnamon OR Cinnamomum OR emblica OR "Phyllanthus emblica" OR "Indian gooseberry" OR "finger citron" OR "Citrus medica" OR "apricot kernel" OR "sea buckthorn" OR Hippophae OR oyster OR Ostrea OR "gorgon fruit" OR Euryale OR "Sichuan pepper" OR Zanthoxylum OR "adzuki bean" OR "rice bean" OR Vigna OR "donkey-hide gelatin" OR malt OR Hordeum OR kelp OR Laminaria OR jujube OR Ziziphus OR "monk fruit" OR Siraitia OR honeysuckle OR Lonicera OR "Chinese olive" OR Canarium OR Houttuynia OR ginger OR Zingiber OR gingerol OR Hovenia OR goji OR wolfberry OR Lycium OR gardenia OR Gardenia OR "Amomum villosum" OR Scaphium OR Sterculia OR poria OR Wolfiporia OR citron OR Mosla OR Elsholtzia OR "peach kernel" OR mulberry OR Morus OR Platycodon OR "Alpinia oxyphylla" OR "lotus leaf" OR "lotus seed" OR Nelumbo OR "radish seed" OR Raphanus OR "Alpinia officinarum" OR Lophatherum OR "fermented soybean" OR douchi OR chrysanthemum OR Chrysanthemum OR chicory OR Cichorium OR "mustard seed" OR Sinapis OR "Polygonatum sibiricum" OR perilla OR Perilla OR kudzu OR Pueraria OR puerarin OR "black sesame" OR Sesamum OR "black pepper" OR "Piper nigrum" OR piperine OR Sophora OR Styphnolobium OR dandelion OR Taraxacum OR honey OR Torreya OR "spiny date seed" OR Imperata OR Phragmites OR "tangerine peel" OR "citrus peel" OR "Citrus reticulata" OR hesperidin OR peppermint OR Mentha OR "coix seed" OR "Job's tears" OR Coix OR "Allium macrostemon" OR "Allium chinense" OR raspberry OR Rubus OR Agastache OR Pogostemon OR "Angelica sinensis" OR "dong quai" OR Kaempferia OR saffron OR Crocus OR crocin OR tsaoko OR "Amomum tsao-ko" OR turmeric OR Curcuma OR curcumin OR "long pepper" OR "Piper longum" OR Codonopsis OR Cistanche OR Dendrobium OR "American ginseng" OR "Panax quinquefolius" OR astragalus OR astragaloside OR Ganoderma OR reishi OR lingzhi OR "Cornus officinalis" OR Gastrodia OR gastrodin OR Eucommia OR ginseng OR "Panax ginseng" OR ginsenoside OR "Lonicera macranthoides" OR coriander OR cilantro OR Coriandrum OR "Rosa rugosa" OR "pine pollen" OR "Pueraria thomsonii" OR "Microcos paniculata" OR selfheal OR "self-heal" OR Prunella):ti,ab,kw

*Search set 2:*

(cardiovascular OR cardiometabolic OR cardiac OR coronary OR "heart disease" OR hypertension OR hypertensive OR "blood pressure" OR dyslipidemia OR hyperlipidemia OR hypercholesterolemia OR cholesterol OR lipid OR lipids OR triglyceride* OR LDL OR HDL OR lipoprotein OR diabet* OR prediabet* OR "insulin resistance" OR insulin OR glycemic OR glucose OR HbA1c OR "metabolic syndrome" OR obesity OR obese OR overweight OR adiposity OR atheroscleros* OR "endothelial function" OR "arterial stiffness" OR "intima-media"):ti,ab,kw

*Search set 3:*

#3	#1 AND #2

**MFH substance list**

The 110-item operational list was compiled from four Chinese MFH catalogue documents: items 1–87 from the 2002 list, 88–93 from the 2019 addition, 94–102 from the 2023 addition, and 103–110 from the 2014 consultation draft. The final eight items were proposed in that draft. Sources and the screening rule for plant parts are given below.

**Catalogue sources**

2002 list (items 1–87): Ministry of Health. List of substances used as both food and medicine, Annex 1 to Wei Fa Jian Fa [2002] No. 51. 28 February 2002. https://www.nhc.gov.cn/sps/c100088/200203/68aa338c814d4a0a9fe5fb6a46717b99.shtml

2019 addition (items 88–93): National Health Commission and State Administration for Market Regulation. Announcement No. 8 of 2019, adding six substances including Angelica sinensis. 25 November 2019. https://www.nhc.gov.cn/wjw/c100175/202001/60977058ce3b4449b1b1d46cff283e73.shtml

2023 addition (items 94–102): National Health Commission and State Administration for Market Regulation. Announcement No. 9 of 2023, adding nine substances including Codonopsis pilosula. 9 November 2023. https://www.nhc.gov.cn/sps/c100088/202311/5b062dd13fe646198b56c7d76a99aab4.shtml

2014 consultation draft (items 103–110): National Health and Family Planning Commission. Notice seeking comments on the catalogue of substances traditionally used as both food and Chinese medicinal materials, Guo Wei Ban Shi Pin Han [2014] No. 975. 28 October 2014. https://www.nhc.gov.cn/sps/c100088/201411/2d5b6eec573e4d51b8d6d1c33a75ff52.shtml

**Plant-part eligibility**

Screening used the reported substance, botanical source and preparation. When a plant part was named, it was compared with the material specified in the catalogue sources. A different part was not treated as equivalent: the screening records, for example, excluded ginkgo leaf extract and American ginseng stem-and-leaf saponins where the listed materials were ginkgo seed and American ginseng root. Whole foods, extracts and derived compounds were eligible within the stated intervention criteria. For extracts without a specified part, decisions were based on the available source-substance and preparation description; a complete part description was not required for every extract. The study descriptions are retained in Supplementary Table 1.

| **No.** | **Chinese name** | **Common name** | **Scientific name used in search** |
| --- | --- | --- | --- |
| 1 | 丁香 | clove | Syzygium aromaticum |
| 2 | 八角茴香 | star anise | Illicium verum |
| 3 | 刀豆 | sword bean | Canavalia gladiata |
| 4 | 小茴香 | fennel | Foeniculum vulgare |
| 5 | 小蓟 | field thistle | Cirsium setosum |
| 6 | 山药 | Chinese yam | Dioscorea opposita / D. polystachya |
| 7 | 山楂 | hawthorn | Crataegus pinnatifida |
| 8 | 马齿苋 | purslane | Portulaca oleracea |
| 9 | 乌梢蛇 | black-striped snake | Zaocys dhumnades |
| 10 | 乌梅 | smoked plum | Prunus mume |
| 11 | 木瓜 | flowering quince | Chaenomeles speciosa |
| 12 | 火麻仁 | hemp seed | Cannabis sativa |
| 13 | 代代花 | bitter orange flower | Citrus aurantium var. amara |
| 14 | 玉竹 | fragrant Solomon's seal | Polygonatum odoratum |
| 15 | 甘草 | licorice | Glycyrrhiza uralensis / G. glabra |
| 16 | 白芷 | Dahurian angelica | Angelica dahurica |
| 17 | 白果 | ginkgo seed | Ginkgo biloba |
| 18 | 白扁豆 | hyacinth bean | Lablab purpureus |
| 19 | 白扁豆花 | hyacinth bean flower | Lablab purpureus (flower) |
| 20 | 龙眼肉（桂圆） | longan | Dimocarpus longan |
| 21 | 决明子 | cassia seed | Senna obtusifolia (Cassia obtusifolia) |
| 22 | 百合 | lily bulb | Lilium brownii / L. lancifolium |
| 23 | 肉豆蔻 | nutmeg | Myristica fragrans |
| 24 | 肉桂 | cinnamon (cassia) | Cinnamomum cassia |
| 25 | 余甘子 | emblic / Indian gooseberry | Phyllanthus emblica |
| 26 | 佛手 | finger citron | Citrus medica var. sarcodactylis |
| 27 | 杏仁（苦杏仁） | bitter apricot kernel | Prunus armeniaca |
| 28 | 沙棘 | sea buckthorn | Hippophae rhamnoides |
| 29 | 牡蛎 | oyster | Ostrea / Crassostrea spp. |
| 30 | 芡实 | gorgon fruit | Euryale ferox |
| 31 | 花椒 | Sichuan pepper | Zanthoxylum bungeanum |
| 32 | 赤小豆 | rice bean / adzuki | Vigna umbellata |
| 33 | 阿胶 | donkey-hide gelatin | Equus asinus (gelatin) |
| 34 | 鸡内金 | chicken gizzard lining | Gallus gallus domesticus |
| 35 | 麦芽 | malt / barley sprout | Hordeum vulgare (germinated) |
| 36 | 昆布 | kelp / kombu | Laminaria japonica / Ecklonia kurome |
| 37 | 枣 | jujube / Chinese date | Ziziphus jujuba |
| 38 | 罗汉果 | monk fruit | Siraitia grosvenorii |
| 39 | 郁李仁 | bush cherry seed | Prunus japonica / P. humilis |
| 40 | 金银花 | honeysuckle flower | Lonicera japonica |
| 41 | 青果 | Chinese olive | Canarium album |
| 42 | 鱼腥草 | houttuynia / fish mint | Houttuynia cordata |
| 43 | 姜（生姜） | ginger | Zingiber officinale |
| 44 | 枳椇子 | Japanese raisin tree seed | Hovenia dulcis / H. acerba |
| 45 | 枸杞子 | goji berry / wolfberry | Lycium barbarum |
| 46 | 栀子 | gardenia fruit | Gardenia jasminoides |
| 47 | 砂仁 | villous amomum | Amomum villosum |
| 48 | 胖大海 | boat-fruited sterculia | Scaphium scaphigerum (Sterculia lychnophora) |
| 49 | 茯苓 | poria | Poria cocos (Wolfiporia extensa) |
| 50 | 香橼 | citron | Citrus medica |
| 51 | 香薷 | Chinese mosla | Mosla chinensis |
| 52 | 桃仁 | peach kernel | Prunus persica |
| 53 | 桑叶 | mulberry leaf | Morus alba (leaf) |
| 54 | 桑椹 | mulberry fruit | Morus alba (fruit) |
| 55 | 桔红（橘红） | red tangerine peel | Citrus reticulata (red peel) |
| 56 | 桔梗 | balloon flower root | Platycodon grandiflorus |
| 57 | 益智仁 | sharp-leaf galangal | Alpinia oxyphylla |
| 58 | 荷叶 | lotus leaf | Nelumbo nucifera (leaf) |
| 59 | 莱菔子 | radish seed | Raphanus sativus (seed) |
| 60 | 莲子 | lotus seed | Nelumbo nucifera (seed) |
| 61 | 高良姜 | lesser galangal | Alpinia officinarum |
| 62 | 淡竹叶 | lophatherum | Lophatherum gracile |
| 63 | 淡豆豉 | fermented soybean (douchi) | Glycine max (fermented) |
| 64 | 菊花 | chrysanthemum | Chrysanthemum morifolium |
| 65 | 菊苣 | chicory | Cichorium intybus |
| 66 | 黄芥子 | white/yellow mustard seed | Sinapis alba |
| 67 | 黄精 | Siberian Solomon's seal | Polygonatum sibiricum |
| 68 | 紫苏 | perilla | Perilla frutescens |
| 69 | 紫苏籽（子） | perilla seed | Perilla frutescens (seed) |
| 70 | 葛根 | kudzu root | Pueraria lobata / P. montana |
| 71 | 黑芝麻 | black sesame | Sesamum indicum |
| 72 | 黑胡椒 | black pepper | Piper nigrum |
| 73 | 槐米 | pagoda tree flower bud | Styphnolobium japonicum (Sophora japonica) |
| 74 | 槐花 | pagoda tree flower | Styphnolobium japonicum (Sophora japonica) |
| 75 | 蒲公英 | dandelion | Taraxacum mongolicum / officinale |
| 76 | 蜂蜜 | honey | Mel |
| 77 | 榧子 | torreya nut | Torreya grandis |
| 78 | 酸枣仁 | spiny jujube seed | Ziziphus jujuba var. spinosa |
| 79 | 鲜白茅根 | lalang grass rhizome | Imperata cylindrica |
| 80 | 鲜芦根 | reed rhizome | Phragmites australis / communis |
| 81 | 蝮蛇 | pit viper | Gloydius / Agkistrodon spp. |
| 82 | 橘皮（陈皮） | dried tangerine peel | Citrus reticulata |
| 83 | 薄荷 | peppermint / mint | Mentha canadensis / haplocalyx |
| 84 | 薏苡仁 | coix seed / Job's tears | Coix lacryma-jobi |
| 85 | 薤白 | Chinese onion / long-stamen onion | Allium macrostemon / A. chinense |
| 86 | 覆盆子 | Chinese raspberry | Rubus chingii |
| 87 | 藿香 | wrinkled giant hyssop | Agastache rugosa |
| 88 | 当归 | Chinese angelica / dong quai | Angelica sinensis |
| 89 | 山柰 | sand ginger | Kaempferia galanga |
| 90 | 西红花（藏红花） | saffron | Crocus sativus |
| 91 | 草果 | tsaoko / black cardamom | Lanxangia tsao-ko (Amomum tsao-ko) |
| 92 | 姜黄 | turmeric | Curcuma longa |
| 93 | 荜茇 | long pepper | Piper longum |
| 94 | 党参 | codonopsis | Codonopsis pilosula |
| 95 | 肉苁蓉（荒漠） | desert cistanche | Cistanche deserticola |
| 96 | 铁皮石斛 | dendrobium | Dendrobium officinale |
| 97 | 西洋参 | American ginseng | Panax quinquefolius |
| 98 | 黄芪 | astragalus / milkvetch | Astragalus membranaceus / mongholicus |
| 99 | 灵芝 | reishi | Ganoderma lucidum |
| 100 | 山茱萸 | Asiatic cornelian cherry | Cornus officinalis |
| 101 | 天麻 | gastrodia | Gastrodia elata |
| 102 | 杜仲叶 | eucommia leaf | Eucommia ulmoides (leaf) |
| 103 | 人参 | Asian ginseng | Panax ginseng |
| 104 | 山银花 | mountain honeysuckle | Lonicera macranthoides 等 |
| 105 | 芫荽 | coriander / cilantro | Coriandrum sativum |
| 106 | 玫瑰花 | rose | Rosa rugosa |
| 107 | 松花粉 | pine pollen | Pinus massoniana / tabuliformis |
| 108 | 粉葛 | thomson kudzu | Pueraria thomsonii |
| 109 | 布渣叶 | microcos leaf | Microcos paniculata |
| 110 | 夏枯草 | selfheal / prunella | Prunella vulgaris |

**S2. Outcome-domain coding dictionary**

Reported outcomes were charted verbatim and coded inductively into the domains below. T1 denotes clinical events and mortality; T2, intermediate cardiometabolic measures; and T3, mechanistic biomarkers. Other outcomes and process measures were retained separately.

| **Outcome domain** | **Representative measures** | **Outcome group** |
| --- | --- | --- |
| Clinical events / mortality | Death, myocardial infarction, stroke, hospitalisation, revascularisation | T1 Clinical events / mortality |
| Cardiac injury / function | Troponin, CK-MB, BNP/NT-proBNP, ejection fraction, longitudinal strain | T2 Intermediate cardiometabolic measures |
| Glycaemic / glucose homeostasis | Fasting glucose, HbA1c, insulin, HOMA-IR, OGTT/postprandial glucose, C-peptide | T2 Intermediate cardiometabolic measures |
| Lipids / lipoproteins | Total, LDL and HDL cholesterol, triglycerides, ApoB, Lp(a), atherogenic indices | T2 Intermediate cardiometabolic measures |
| Blood pressure / haemodynamic | Systolic and diastolic blood pressure, mean arterial pressure, heart rate | T2 Intermediate cardiometabolic measures |
| Vascular / endothelial function | Flow-mediated dilation, pulse-wave velocity, carotid intima-media thickness | T2 Intermediate cardiometabolic measures |
| Renal function | Creatinine, urea, eGFR, uric acid, albuminuria | T2 Intermediate cardiometabolic measures |
| Anthropometry / adiposity | Body weight, BMI, waist circumference, body fat and composition | T2 Intermediate cardiometabolic measures |
| Composite cardiometabolic risk | Framingham/ASCVD risk scores, metabolic-syndrome status | T2 Intermediate cardiometabolic measures |
| Inflammation / adipokines | C-reactive protein, interleukins, TNF, leptin, adiponectin, ICAM/VCAM | T3 Mechanistic biomarkers |
| Oxidative stress / antioxidant | Malondialdehyde, total antioxidant capacity, SOD, glutathione peroxidase, oxidised LDL | T3 Mechanistic biomarkers |
| Liver | ALT, AST, GGT, hepatic steatosis and fibrosis | T3 Mechanistic biomarkers |
| Haemostasis / thrombosis | Platelet aggregation, fibrinogen, PAI-1 | T3 Mechanistic biomarkers |
| Hormonal | Sex steroids, thyroid hormones, cortisol, IGF-1 | T3 Mechanistic biomarkers |
| Energy / appetite | Energy expenditure, thermogenesis, satiety, ghrelin, PYY | Other outcomes / process measures |
| Dietary intake / compliance | Energy and macronutrient intake, adherence | Other outcomes / process measures |
| Physical activity / fitness | VO2, exercise capacity, walk distance | Other outcomes / process measures |
| Safety / tolerability | Adverse events, routine haematology and biochemistry panels | Other outcomes / process measures |
| Mental health / quality of life | Cognition, mood, depression, anxiety, quality of life | Other outcomes / process measures |
| Other molecular / microbiome | Gene or microRNA expression, gut microbiota, pharmacokinetics | Other outcomes / process measures |
| Other / unclassified | Measures not assigned to another domain | Other outcomes / process measures |

*In Supplementary Table 1, brackets show the outcome group and domains. For multidomain records, each domain contributes to study-level coverage; the first assigned domain determines the outcome-level group. Study-level T1–T3 totals require at least one record carrying the corresponding group label. The glycaemic AUC category includes glucose or insulin AUC, iAUC, netAUC and spelled-out area-under-the-curve descriptions. Each study contributes once, even when it reports several such measures. Lipid AUC measures are not included in this category.*

**S3. Included studies and stratified outcome summaries**

*The 416 study numbers match Supplementary Table 1. Reports linked to the same study are listed under one number; the bibliography contains 420 reports.*

***Outcome-domain coverage by duration and formulation***

| **Study group** | **n** | **Glycaemic** | **Lipid** | **Anthropo-metry** | **Blood pressure** | **Inflam-mation** | **Oxidative stress** |
| --- | --- | --- | --- | --- | --- | --- | --- |
| Duration |  |  |  |  |  |  |  |
| Acute dosing | 61 | 54 (88.5) | 10 (16.4) | 9 (14.8) | 9 (14.8) | 2 (3.3) | 0 (0.0) |
| Repeated dosing | 339 | 280 (82.6) | 245 (72.3) | 207 (61.1) | 132 (38.9) | 125 (36.9) | 71 (20.9) |
| Mixed dosing | 2 | 2 (100.0) | 0 (0.0) | 1 (50.0) | 1 (50.0) | 0 (0.0) | 0 (0.0) |
| Unclear duration | 14 | 9 (64.3) | 5 (35.7) | 5 (35.7) | 1 (7.1) | 3 (21.4) | 4 (28.6) |
| Formulation |  |  |  |  |  |  |  |
| Foods / minimally processed | 96 | 85 (88.5) | 65 (67.7) | 52 (54.2) | 33 (34.4) | 22 (22.9) | 20 (20.8) |
| Extracts / fractions | 108 | 86 (79.6) | 53 (49.1) | 43 (39.8) | 38 (35.2) | 24 (22.2) | 16 (14.8) |
| Isolated / enriched constituents | 103 | 79 (76.7) | 76 (73.8) | 68 (66.0) | 34 (33.0) | 51 (49.5) | 22 (21.4) |
| Multiple types | 1 | 1 (100.0) | 1 (100.0) | 1 (100.0) | 0 (0.0) | 0 (0.0) | 0 (0.0) |
| Unclear formulation | 108 | 94 (87.0) | 65 (60.2) | 58 (53.7) | 38 (35.2) | 33 (30.6) | 17 (15.7) |

Values are n (% of studies in each row). Formulation groups are based on the reported substance and preparation.

1. Ab Wahab SZ, Nik Hussain NH, Zakaria R, Abdul Kadir A, Mohamed N, Tohit NM, et al. Long-term effects of honey on cardiovascular parameters and anthropometric measurements of postmenopausal women. Complementary Therapies in Medicine. 2018;41:154-160.

2. Abbood MS. Hypolipidaemic and anti-inflammatory effects of curcumin versus atorvastatin in type 2 diabetic patients. International Journal of Pharmaceutical Sciences Review and Research. 2018;49(1):1-7.

3. Abdulrhman MM, El-Hefnawy MH, Aly RH, Shatla RH, Mamdouh RM, Mahmoud DM, et al. Metabolic effects of honey in type 1 diabetes mellitus: A randomized crossover pilot study. Journal of Medicinal Food. 2013;16(1):66-72.

4. Abedimanesh N, Bathaie SZ, Abedimanesh S, Motlagh B, Separham A, Ostadrahimi A. Saffron and crocin improved appetite, dietary intakes and body composition in patients with coronary artery disease. Journal of Cardiovascular and Thoracic Research. 2017;9(4):200-208.

5. Abedimanesh S, Bathaie SZ, Ostadrahimi A, Jafarabadi MA, Sadeghi MT. The effect of crocetin supplementation on markers of atherogenic risk in patients with coronary artery disease: a pilot, randomized, double-blind, placebo-controlled clinical trial. Food & function. 2019;10(11):7461-7475.

6. Adab Z, Eghtesadi S, Vafa MR, Heydari I, Shojaii A, Haqqani H, et al. Effect of turmeric on glycemic status, lipid profile, hs-CRP, and total antioxidant capacity in hyperlipidemic type 2 diabetes mellitus patients. Phytotherapy Research. 2019;33(4):1173-1181.

7. Adibian M, Hodaei H, Nikpayam O, Sohrab G, Hekmatdoost A, Hedayati M. The effects of curcumin supplementation on high-sensitivity C-reactive protein, serum adiponectin, and lipid profile in patients with type 2 diabetes: A randomized, double-blind, placebo-controlled trial. Phytotherapy Research. 2019;33(5):1374-1383.

8. af Geijerstam P, Joelsson A, Radholm K, Nystrom FH. A low dose of daily licorice intake affects renin, aldosterone, and home blood pressure in a randomized crossover trial. American Journal of Clinical Nutrition. 2024;119(3):682-691.

9. Afiat M, Amini E, Ghazanfarpour M, Nouri B, Mousavi MS, Babakhanian M, et al. The Effect of Short-term Treatment with Fennel on Lipid Profile in Postmenopausal Women: A Randomized Controlled Trial. J Menopausal Med. 2018;24(1):29-33.

10. Afshar GV, Rasmi Y, Yagmaye P, Khadem-Ansari MH, Makhdomi K, Rasooli J. The effects of nano-curcumin supplementation on serum level of hs-CRP, adhesion molecules, and lipid profiles in hemodialysis patients, a randomized controlled clinical trial. Iranian Journal of Kidney Diseases. 2020;14(1):52-61.

11. Ahmad H, Kashif S, Afreen A, Safdar M, Ahmed Z. Comparative effect of Fenugreek and Cinnamon on management of newly diagnosed cases of Type-2 Diabetes Mellitus. FOOD SCIENCE AND TECHNOLOGY. 2022;42.

12. Ahn CM, Hong SJ, Choi SC, Park JH, Kim JS, Lim DS. Red ginseng extract improves coronary flow reserve and increases absolute numbers of various circulating angiogenic cells in patients with first ST-segment elevation acute myocardial infarction. Phytotherapy Research. 2011;25(2):239-249.

13. Akilen R, Tsiami A, Devendra D, Robinson N. Glycated haemoglobin and blood pressure-lowering effect of cinnamon in multi-ethnic Type 2 diabetic patients in the UK: A randomized, placebo-controlled, double-blind clinical trial. Diabetic Medicine. 2010;27(10):1159-1167.

14. Akram S, Zafar S, Khalid SN, Farooq U, Shah HBU, Ghazanfar M, et al. Determination of Chemical Composition and Therapeutic Potential of Chicory (Chichorium Intybus) Leaves Powder in Management of Hyperlipidemia in Human Subjects: A Randomized Control Trial. Journal of Population Therapeutics and Clinical Pharmacology. 2023;30(18):1113-1120.

15. Al-Tamimi AM, Petrisko M, Hong MY, Rezende L, Clayton ZS, Kern M. Honey does not adversely impact blood lipids of adult men and women: a randomized cross-over trial. Nutrition Research. 2020;74:87-95.

16. Alhashemi SH, Mohammadpour AH, Heidari R, Nikoo MH, Nemati MH, Vazin A. The effect of nanocurcumin on the incidence of atrial fibrillation, and markers of inflammation and oxidative stress level after coronary artery bypass graft surgery: A randomized, double-blind, placebo-controlled clinical study. Avicenna Journal of Phytomedicine. 2022;12(5):503-513.

17. Alimoradian A, Mohajerani E, Navabi A, Sade BS, Asafari M. Processed and Unprocessed Honey: A Comparative Study on the Effect of Honey on Blood Sugar Levels in Diabetics. Journal of Kerman University of Medical Sciences. 2025;32(1).

18. Alizadeh M, Namazi N, Mirtaheri E, Sargheini N, Kheirouri S. Changes of insulin resistance and adipokines following supplementation with Glycyrrhiza glabra L. Extract in combination with a low-calorie diet in overweight and obese subjects: A randomized double blind clinical trial. Advanced Pharmaceutical Bulletin. 2018;8(1):123-130.

19. Alizadeh-Navaei R, Roozbeh F, Saravi M, Pouramir M, Jalali F, Moghadamnia AA. Investigation of the effect of ginger on the lipid levels: A double blind controlled clinical trial. Saudi Medical Journal. 2008;29(9):1280-1284.

20. Almyali AAH, Al-Chlaihawi MK, Hameed M, Kadhim S, Al-Hadrawi HA. Effect of boiled and macerated cinnamon sticks and powder on diabetic and biochemical profile in patients with type 2 diabetes mellitus. Wiad Lek. 2026;79(1):105-113.

21. Altschuler JA, Casella SJ, MacKenzie TA, Curtis KM. The effect of cinnamon on A1C among adolescents with type 1 diabetes. Diabetes Care. 2007;30(4):813-816.

22. Alvarenga L, Cardozo LFMF, Ribeiro-Alves M, Damasceno NRT, Berretta AA, Lima JA, et al. Effects of turmeric extract supplementation on the lipid and lipoprotein subfraction profile in hemodialysis patients: A randomised, double-blind, crossover and controlled trial. Phytotherapy Research. 2023;37(8):3424-3437.

23. Alwi I, Santoso T, Suyono S, Sutrisna B, Suyatna FD, Kresno SB, et al. The effect of curcumin on lipid level in patients with acute coronary syndrome. Acta medica Indonesiana. 2008;40(4):201-210.

24. Amagase H, Nance DM. Lycium barbarum increases caloric expenditure and decreases waist circumference in healthy overweight men and women: Pilot study. Journal of the American College of Nutrition. 2011;30(5):304-309.

25. Amin F, Islam N, Anila N, Gilani AH. Clinical efficacy of the co-administration of Turmeric and Black seeds (Kalongi) in metabolic syndrome - A double blind randomized controlled trial - TAK-MetS trial. Complementary Therapies in Medicine. 2015;23(2):165-174.

26. Amirkhani Z, Azarbayjani MA, Peeri M, Homaei HM. Effect of combining resistance training and curcumin supplementation on lipid profile in obese women. Iran J Obstet Gynecol Infertil. 2017;20(9):24-32.

27. Amirkhanloo F, Esmaeilzadeh S, Mirabi P, Abedini A, Amiri M, Saghebi R, et al. Comparison of Foeniculum Vulgare versus metformin on insulin resistance and anthropometric indices of women with polycystic ovary, an open-label controlled trial study. Obesity Medicine. 2022;31.

28. Anderson RA, Zhan Z, Luo R, Guo X, Guo Q, Zhou J, et al. Cinnamon extract lowers glucose, insulin and cholesterol in people with elevated serum glucose. J Tradit Complement Med. 2016;6(4):332-336.

29. Aoe S, Ohtoshi H, Nakamura F. Effects of Daily Kombu (Laminaria japonica) Intake on Body Composition, Blood Pressure, and Fecal Microbiota in Healthy Adult Japanese: A Randomized, Double-Blind Study. Food Sci Nutr. 2025;13(5):e70298.

30. Aoe S, Yamanaka C, Ohtoshi H, Nakamura F, Fujiwara S. Effects of daily kelp (Laminaria japonica) intake on body composition, serum lipid levels, and thyroid hormone levels in healthy japanese adults: A randomized, double-blind study. Marine Drugs. 2021;19(7).

31. Arablou T, Aryaeian N, Valizadeh M, Sharifi F, Hosseini A, Djalali M. The effect of ginger consumption on glycemic status, lipid profile and some inflammatory markers in patients with type 2 diabetes mellitus. International Journal of Food Sciences and Nutrition. 2014;65(4):515-520.

32. Arzati MM, Honarvar NM, Saedisomeolia A, Anvari S, Effatpanah M, Arzati RM, et al. The effects of ginger on fasting blood sugar, hemoglobin A1c, and lipid profiles in patients with type 2 diabetes. International Journal of Endocrinology and Metabolism. 2017;15(4).

33. Asai A, Nakagawa K, Higuchi O, Kimura T, Kojima Y, Kariya J, et al. Effect of mulberry leaf extract with enriched 1-deoxynojirimycin content on postprandial glycemic control in subjects with impaired glucose metabolism. Journal of Diabetes Investigation. 2011;2(4):318-323.

34. Asghari KM, Saleh P, Salekzamani Y, Dolatkhah N, Aghamohammadzadeh N, Hashemian M. The effect of curcumin and high-content eicosapentaenoic acid supplementations in type 2 diabetes mellitus patients: a double-blinded randomized clinical trial. Nutrition and Diabetes. 2024;14(1).

35. Asher GN, Viera AJ, Weaver MA, Dominik R, Caughey M, Hinderliter AL. Effect of hawthorn standardized extract on flow mediated dilation in prehypertensive and mildly hypertensive adults: A randomized, controlled cross-over trial. BMC Complementary and Alternative Medicine. 2012;12.

36. Askari F, Rashidkhani B, Hekmatdoost A. Cinnamon may have therapeutic benefits on lipid profile, liver enzymes, insulin resistance, and high-sensitivity C-reactive protein in nonalcoholic fatty liver disease patients. Nutrition Research. 2014;34(2):143-148.

37. Aslanabadi N, Entezari-Maleki T, Rezaee H, Jafarzadeh HR, Vahedpour R. Curcumin for the prevention of myocardial injury following elective percutaneous coronary intervention; a pilot randomized clinical trial. European Journal of Pharmacology. 2019;858.

38. Atakan A, Kabaran S, Eren FH. The effects of dietary intervention with turmeric on lipid profile and anthropometric indices in overweight/ obese women with hyperlipidemia: a randomized controlled clinical trial. Progress in Nutrition. 2022;24(2).

39. Atashak S, Peeri M, Azarbayjani MA, Stannard SR, Haghighi MM. Obesity-related cardiovascular risk factors after long- term resistance training and ginger supplementation. J Sports Sci Med. 2011;10(4):685-91.

40. Attari VE, Mahluji S, Jafarabadi MA, Ostadrahimi A. Effects of supplementation with ginger (Zingiber officinale Roscoe) on Serum glucose, lipid profile and oxidative stress in obese women: A randomized, placebo-controlled clinical trial. Pharmaceutical Sciences. 2015;21(4):184-191.

41. Azimi P, Ghiasvand R, Feizi A, Hosseinzadeh J, Bahreynian M, Hariri M, et al. Effect of cinnamon, cardamom, saffron and ginger consumption on blood pressure and a marker of endothelial function in patients with type 2 diabetes mellitus: A randomized controlled clinical trial. Blood Pressure. 2016;25(3):133-140.

42. Babaahmadi-Rezaei H, Kheirollah A, Hesam S, Ayashi S, Aberumand M, Adel MH, et al. Decreased lipoprotein (A) and serum high-sensitivity c-reactive protein levels in male patients with atherosclerosis after supplementation with ginger: A randomized controlled trial. ARYA Atherosclerosis. 2020;16(4):1-8.

43. Babaei H, Forouzandeh F, Maghsoumi-Norouzabad L, Yousefimanesh HA, Ravanbakhsh M, Zare Javid A. Effects of chicory leaf extract on serum oxidative stress markers, lipid profile and periodontal status in patients with chronic periodontitis. J Am Coll Nutr. 2018;37(6):479-486.

44. Babamiri S, Mojani Qomi MS, Shiehmorteza M. The Efficacy of italic Ganoderma lucidum in Overweight Individuals: A Randomized Placebo-controlled trial. Mediterranean Journal of Nutrition and Metabolism. 2022;15(2):263-271.

45. Bahar A, Ebrahim H, Nafchi AM, Khosravi A, Delvarianzadeh M. Effect of bulk bread formulated with Portulaca oleracea on liver enzymes, and oxidative stress in patients with type 2 diabetes and hyperlipidemia: a parallel randomized controlled trial. Koomesh. 2022;24(1):139-146.

46. Bahrami M, Ataie-Jafari A, Hosseini S, Foruzanfar MH, Rahmani M, Pajouhi M. Effects of natural honey consumption in diabetic patients: an 8-week randomized clinical trial. International Journal of Food Sciences and Nutrition. 2009;60(7):618-626.

47. Bakhshi M, Rafraf M, Haghravan S, Asghari Jafarabadi M, Jafari A. The effect of ginger supplementation and high-intensity interval training (HIIT) on anthropometric indices and serum level of irisin in obese men [in Persian]. Iran J Endocrinol Metab. 2019;21(2):83-91.

48. Bang H, Kwak JH, Ahn HY, Shin DY, Lee JH. Korean red ginseng improves glucose control in subjects with impaired fasting glucose, impaired glucose tolerance, or newly diagnosed type 2 diabetes mellitus. Journal of Medicinal Food. 2014;17(1):128-134.

49. Barber-Chamoux N, Milenkovic D, Verny MA, Habauzit V, Pereira B, Lambert C, et al. Substantial Variability Across Individuals in the Vascular and Nutrigenomic Response to an Acute Intake of Curcumin: A Randomized Controlled Trial. Molecular nutrition & food research. 2018;62(5).

50. Bateni Z, Behrouz V, Rahim HR, Hedayati M, Afsharian S, Sohrab G. Effects of nano-curcumin supplementation on oxidative stress, systemic inflammation, adiponectin, and NF-κB in patients with metabolic syndrome: A randomized, double-blind clinical trial. Journal of Herbal Medicine. 2022;31.

51. Bateni Z, Rahimi HR, Hedayati M, Afsharian S, Goudarzi R, Sohrab G. The effects of nano-curcumin supplementation on glycemic control, blood pressure, lipid profile, and insulin resistance in patients with the metabolic syndrome: A randomized, double-blind clinical trial. Phytotherapy Research. 2021;35(7):3945-3953.

52. Baum L, Cheung SKK, Mok VCT, Lam LCW, Leung VPY, Hui E, et al. Curcumin effects on blood lipid profile in a 6-month human study. Pharmacological Research. 2007;56(6):509-514.

53. Behrouz V, Sohrab G, Hedayati M, Sedaghat M. Inflammatory markers response to crocin supplementation in patients with type 2 diabetes mellitus: A randomized controlled trial. Phytotherapy Research. 2021;35(7):4022-4031.

54. Bell ZW, Canale RE, Bloomer RJ. A dual investigation of the effect of dietary supplementation with licorice flavonoid oil on anthropometric and biochemical markers of health and adiposity. Lipids in Health and Disease. 2011;10.

55. Bernardo MA, Silva ML, Santos E, Moncada MM, Brito J, Proenca L, et al. Effect of Cinnamon Tea on Postprandial Glucose Concentration. Journal of Diabetes Research. 2015;2015.

56. Bessell E, Fuller NR, Markovic TP, Lau NS, Burk J, Hendy C, et al. Effects of α-Cyclodextrin on Cholesterol Control and Hydrolyzed Ginseng Extract on Glycemic Control in People With Prediabetes: A Randomized Clinical Trial. JAMA Netw Open. 2020;3(11):e2023491.

57. Boers HM, van Dijk TH, Duchateau GS, Mela DJ, Hiemstra H, Hoogenraad AR, et al. Effect of mulberry fruit extract on glucose fluxes after a wheat porridge meal: a dual isotope study in healthy human subjects. European Journal of Clinical Nutrition. 2023;77(7):741-747.

58. Bordia A, Verma SK, Srivastava KC. Effect of ginger (Zingiber officinale Rosc.) and fenugreek (Trigonella foenumgraecum L.) on blood lipids, blood sugar and platelet aggregation in patients with coronary artery disease. Prostaglandins Leukotrienes and Essential Fatty Acids. 1997;56(5):379-384.

59. Braga Tibaes JR, Martins LB, Rodrigues AMDS, Amaral MHA, Teixeira AL, Ferreira AVM. Ginger supplementation does not increase energy expenditure in female adults. Nutrition. 2022;103-104.

60. Bupparenoo P, Pakchotanon R, Narongroeknawin P, Asavatanabodee P, Chaiamnuay S. Effect of Curcumin on Serum Urate in Asymptomatic Hyperuricemia: A Randomized Placebo-Controlled Trial. Journal of dietary supplements. 2021;18(3):248-260.

61. Caia H, Liu F, Zuo P, Huang G, Song Z, Wang T, et al. Practical application of antidiabetic efficacy of lycium barbarum polysaccharide in patients with type 2 diabetes. Medicinal Chemistry. 2015;11(4):383-390.

62. Campbell MS, Ouyang A, I.M K, Charnigo RJ, Westgate PM, Fleenor BS. Influence of enhanced bioavailable curcumin on obesity-associated cardiovascular disease risk factors and arterial function: A double-blinded, randomized, controlled trial. Nutrition. 2019;62:135-139.

63. Carvalho GCN, Lira-Neto JCG, Araujo MFM, Freitas RWJF, Zanetti ML, Damasceno MMC. Effectiveness of ginger in reducing metabolic levels in people with diabetes: a randomized clinical trial. Revista latino-americana de enfermagem. 2020;28:e3369.

64. Cha TW, Kim M, Chae JS, Lee JH. Blood pressure-lowering effect of Korean red ginseng associated with decreased circulating Lp-PLA 2 activity and lysophosphatidylcholines and increased dihydrobiopterin level in prehypertensive subjects. Hypertension Research. 2016;39(6):449-456.

65. Chandra K, Jain V, Jabin A, Dwivedi S, Joshi S, Ahmad S, et al. Effect of Cichorium intybus seeds supplementation on the markers of glycemic control, oxidative stress, inflammation, and lipid profile in type 2 diabetes mellitus: A randomized, double-blind placebo study. Phytotherapy Research. 2020;34(7):1609-1618.

66. Chauhan P, Prasad GBKS. Enhanced therapeutic efficacy of nanocurcumin over free curcumin in the management of diabetes mellitus. PharmaNutrition. 2025;33.

67. Cheng Y, Zhang H, Zhu Y, Xue Z, Yan M, Wang H, et al. Effects of fructose from apple and honey on serum uric acid in young Chinese: Randomized crossover trials. Asia Pacific Journal of Clinical Nutrition. 2022;31(1):87-96.

68. Cho YH, Ahn SC, Lee SY, Jeong DW, Choi EJ, Kim YJ, et al. Effect of Korean red ginseng on insulin sensitivity in non-diabetic healthy overweight and obese adults. Asia Pacific Journal of Clinical Nutrition. 2013;22(3):365-371.

69. Chu TT, Benzie IF, Lam CW, Fok BS, Lee KK, Tomlinson B. Study of potential cardioprotective effects of Ganoderma lucidum (Lingzhi): results of a controlled human intervention trial. The British journal of nutrition. 2012;107(7):1017-1027.

70. Chuengsamarn S, Rattanamongkolgul S, Luechapudiporn R, Phisalaphong C, Jirawatnotai S. Curcumin extract for prevention of type 2 diabetes. Diabetes Care. 2012;35(11):2121-2127.

71. Chuengsamarn S, Rattanamongkolgul S, Phonrat B, Tungtrongchitr R, Jirawatnotai S. Reduction of atherogenic risk in patients with type 2 diabetes by curcuminoid extract: A randomized controlled trial. Journal of Nutritional Biochemistry. 2014;25(2):144-150.

72. Chung HI, Kim J, Kim JY, Kwon O. Acute intake of mulberry leaf aqueous extract affects postprandial glucose response after maltose loading: Randomized double-blind placebo-controlled pilot study. JOURNAL OF FUNCTIONAL FOODS. 2013;5(3):1502-1506.

73. Chung IM, Lim JW, Pyun WB, Kim H. Korean Red Ginseng Improves Vascular Stiffness in Patients with Coronary Artery Disease. Journal of Ginseng Research. 2010;34(3):212-218.

74. Crawford P. Effectiveness of cinnamon for lowering hemoglobin A1C in patients with type 2 diabetes: A randomized, controlled trial. Journal of the American Board of Family Medicine. 2009;22(5):507-512.

75. Damavandi RD, Shidfar F, Najafi M, Janani L, Masoodi M, Heshmati J, et al. Effect of portulaca oleracea (purslane) extract on inflammatory factors in nonalcoholic fatty liver disease: A randomized, double-blind clinical trial. JOURNAL OF FUNCTIONAL FOODS. 2023;102.

76. Darmian MA, Hoseini R, Amiri E, Golshani S. Downregulated hs-CRP and MAD, upregulated GSH and TAC, and improved metabolic status following combined exercise and turmeric supplementation: a clinical trial in middle-aged women with hyperlipidemic type 2 diabetes. Journal of Diabetes and Metabolic Disorders. 2022;21(1):275-283.

77. Dascalu A, Sievenpiper JL, Jenkins AL, Stavro MP, Leiter LA, Arnason JT, et al. Five batches representative of Ontario-grown American ginseng root produce comparable reductions of postprandial glycemia in healthy individuals. Canadian Journal of Physiology and Pharmacology. 2007;85(9):856-864.

78. Dastani M, Bigdelu L, Hoseinzadeh M, Rahimi HR, Karimani A, Mohammadpour AH, et al. The effects of curcumin on the prevention of atrial and ventricular arrhythmias and heart failure in patients with unstable angina: A randomized clinical trial. Avicenna Journal of Phytomedicine. 2019;9(1):1-9.

79. Dastani M, Rahimi HR, Askari VR, Jaafari MR, Jarahi L, Yadollahi A, et al. Three months of combination therapy with nano-curcumin reduces the inflammation and lipoprotein (a) in type 2 diabetic patients with mild to moderate coronary artery disease: Evidence of a randomized, double-blinded, placebo-controlled clinical trial. BioFactors. 2023;49(1):108-118.

80. Dastgheib M, Barati-Boldaji R, Bahrampour N, Taheri R, Borghei M, Amooee S, et al. A comparison of the effects of cinnamon, ginger, and metformin consumption on metabolic health, anthropometric indices, and sexual hormone levels in women with poly cystic ovary syndrome: A randomized double-blinded placebo-controlled clinical trial. Front Nutr. 2022;9:1071515.

81. Dastkhosh A, Behrouz V, Sohrab G, Nikpayam O, Sedaghat M, Ebrahimof S. What are the effects of crocin supplementation on lipid profile and oxidative stress in patients with type 2 diabetes? A randomized clinical trial. JOURNAL OF FUNCTIONAL FOODS. 2024;121.

82. Davari M, Hashemi R, Mirmiran P, Hedayati M, Sahranavard S, Bahreini S, et al. Effects of cinnamon supplementation on expression of systemic inflammation factors, NF-kB and Sirtuin-1 (SIRT1) in type 2 diabetes: A randomized, double blind, and controlled clinical trial. Nutrition Journal. 2020;19(1).

83. de Sousa Guardiano Reis PC, Alves AGP, Guillo LA, de Sousa MA, Trindade NR, Silva MS. Curcumin supplementation reduces blood glucose and serum lipids of Brazilian women with high waist circumference: a randomized clinical trial. Archives of endocrinology and metabolism. 2022;66(6):800-807.

84. Delgadillo-Centeno JS, Grover-Paez F, Hernandez-Gonzalez SO, Ramos-Zavala MG, Cardona-Muller D, Lopez-Castro A, et al. Cinnamomum cassia on Arterial Stiffness and Endothelial Dysfunction in Type 2 Diabetes Mellitus: Outcomes of a Randomized, Double-Blind, Placebo-Controlled Clinical Trial. Journal of Medicinal Food. 2023;26(6):428-434.

85. Despland C, Walther B, Kast C, Campos V, Rey V, Stefanoni N, et al. A randomized-controlled clinical trial of high fructose diets from either Robinia honey or free fructose and glucose in healthy normal weight males. Clinical Nutrition ESPEN. 2017;19:16-22.

86. Devang N, Adhikari P, Nandini M, Satyamoorthy K, Rai PS. Effect of licorice on patients with HSD11B1 gene polymorphisms- a pilot study. Journal of Ayurveda and Integrative Medicine. 2021;12(1):131-135.

87. Di Pierro F, Bressan A, Ranaldi D, Rapacioli G, Giacomelli L, Bertuccioli A. Potential role of bioavailable curcumin in weight loss and omental adipose tissue decrease: Preliminary data of a randomized, controlled trial in overweight people with metabolic syndrome. Preliminary study. European Review for Medical and Pharmacological Sciences. 2015;19(21):4195-4202.

88. Dickman JR, Koenig RT, Ji LL. American ginseng supplementation induces an oxidative stress in postmenopausal women. Journal of the American College of Nutrition. 2009;28(2):219-228.

89. Ding F, Wang Q, Xie C, Wang M, Zhang L, Gao M, et al. The impact of mulberry leaf extract at three different levels on reducing the glycemic index of white bread. PLoS ONE. 2023;18(8).

90. Djaja N. The Effect of Adding Job's Tears to Yogurt on Plasma Glycated Albumin, Weight, and Lipid Profile in Patients with Type 2 Diabetes Mellitus: A Randomized Controlled Trial. Journal of Nutrition and Metabolism. 2022;2022.

91. Ebrahimi F, Sahebkar A, Aryaeian N, Pahlavani N, Fallah S, Moradi N, et al. Effects Of Saffron Supplementation On Inflammation And Metabolic Responses In Type 2 Diabetic Patients: A Randomized, Double-Blind, Placebo-Controlled Trial. Diabetes, Metabolic Syndrome and Obesity. 2019;12:2107-2115.

92. El Gayar MH, Aboromia MMM, Ibrahim NA, Abdel Hafiz MH. Effects of ginger powder supplementation on glycemic status and lipid profile in newly diagnosed obese patients with type 2 diabetes mellitus. Obes Med. 2019;14:100094.

93. El-Rakabawy OM, Elkholy AA, Mahfouz AA, Abdelsalam MM, El Wakeel LM. Curcumin supplementation improves the clinical outcomes of patients with diabetes and atherosclerotic cardiovascular risk. Scientific reports. 2025;15(1):28358.

94. Esmaillzadeh A, Zakizadeh E, Faghihimani E, Gohari M, Jazayeri S. The effect of purslane seeds on glycemic status and lipid profiles of persons with type 2 diabetes: A randomized controlled cross-over clinical trial. Journal of Research in Medical Sciences. 2015;20(1):47-53.

95. Fadai F, Mousavi B, Ashtari Z, Ali Beigi N, Farhang S, Hashempour S, et al. Saffron aqueous extract prevents metabolic syndrome in patients with schizophrenia on olanzapine treatment: A randomized triple blind placebo controlled study. Pharmacopsychiatry. 2014;47(4-5):156-161.

96. Fadheel QJ. The potential effects of cinnamon, cumin, and Rosuvastatin on lipid profile among Iraqi patients with dyslipidemia. Wiadomosci lekarskie. 2025;78(10):2008-2018.

97. Fagundes GBP, Rodrigues AMDS, Martins LB, Monteze NM, Correia MITD, Teixeira AL, et al. Acute effects of dry extract of ginger on energy expenditure in eutrophic women: A randomized clinical trial. Clinical Nutrition ESPEN. 2021;41:168-174.

98. Faraji S, Hosseini Azar MRM, Alizadeh M. Brewed chicory leaf consumption has unexpected side effects along beneficial effects on liver enzymes in non-alcoholic fatty liver disease patients. Journal of Herbal Medicine. 2022;34.

99. Farakla I, Koui E, Arditi J, Papageorgiou I, Bartzeliotou A, Papadopoulos GE, et al. Effect of honey on glucose and insulin concentrations in obese girls. European Journal of Clinical Investigation. 2019;49(2).

100. Farhadnejad H, Hedayati M, Sahranavard S, Jahromi MK, Asghari G, Teymoori F, et al. Effect of dried Ziziphus Jujube Consumption on Plasma Insulin, Blood Pressure, Oxidative Stress, and Advanced Glycation End Products in Diabetic Patients with Excess Weight: A Randomized Controlled Trial. International Journal of Preventive Medicine. 2025;16.

101. Farhangi MA, Javid AZ, Dehghan P. The effect of enriched chicory inulin on liver enzymes, calcium homeostasis and hematological parameters in patients with type 2 diabetes mellitus: A randomized placebo-controlled trial. Primary Care Diabetes. 2016;10(4):265-271.

102. Farzad L, Dolati S, Far AH. The Effect of Aerobic Training and Supplementation of Curcumin on the Glycemic Markers and Level of Myeloperoxidase Enzyme of Overweight Women. Romanian Journal of Diabetes, Nutrition and Metabolic Diseases. 2020;27(3):281-288.

103. Fatima N, Pingali U, Muralidhar N. Study of pharmacodynamic interaction of Phyllanthus emblica extract with clopidogrel and ecosprin in patients with type II diabetes mellitus. Phytomedicine. 2014;21(5):579-585.

104. Fatima N, Pingali U, Pilli R. Evaluation of Phyllanthus emblica extract on cold pressor induced cardiovascular changes in healthy human subjects. Pharmacognosy Research. 2014;6(1):29-35.

105. Ferguson JJA, Wolska A, Remaley AT, Stojanovski E, MacDonald-Wicks L, Garg ML. Bread enriched with phytosterols with or without curcumin modulates lipoprotein profiles in hypercholesterolaemic individuals. A randomised controlled trial. Food & function. 2019;10(5):2515-2527.

106. Fogelman Y, Gaitini D, Carmeli E. Antiatherosclerotic effects of licorice extract supplementation on hypercholesterolemic patients: Decreased CIMT, reduced plasma lipid levels, and decreased blood pressure. Food and Nutrition Research. 2016;60.

107. Funamoto M, Shimizu K, Sunagawa Y, Katanasaka Y, Miyazaki Y, Kakeya H, et al. Effects of Highly Absorbable Curcumin in Patients with Impaired Glucose Tolerance and Non-Insulin-Dependent Diabetes Mellitus. Journal of Diabetes Research. 2019;2019.

108. Funamoto M, Sunagawa Y, Katanasaka Y, Kato T, Funada J, Ajiro Y, et al. Effects of high-absorption curcumin for the prevention of hypertensive heart disease: a double-blind, placebo-controlled, randomized clinical study. Eur Heart J Open. 2022;2(5):oeac057.

109. Funamoto M, Sunagawa Y, Katanasaka Y, Miyazaki Y, Imaizumi A, Kakeya H, et al. Highly absorptive curcumin reduces serum atherosclerotic low-density lipoprotein levels in patients with mild COPD. International Journal of COPD. 2016;11(1):2029-2034.

110. Ganjali S, Sahebkar A, Mahdipour E, Jamialahmadi K, Torabi S, Akhlaghi S, et al. Investigation of the effects of curcumin on serum cytokines in obese individuals: A randomized controlled trial. The Scientific World Journal. 2014;2014.

111. Ghaffari A, Rafraf M, Navekar R, Sepehri B, Asghari-Jafarabadi M, Ghavami SM. Effects of turmeric on homocysteine and Fetuin-A in patients with nonalcoholic fatty liver disease: A randomized Double-Blind placebo-controlled study. Iranian Red Crescent Medical Journal. 2017;19(4).

112. Ghaffari A, Rafraf M, Navekar R, Sepehri B, Asghari-Jafarabadi M, Ghavami SM. Turmeric and chicory seed have beneficial effects on obesity markers and lipid profile in non-alcoholic fatty liver disease (NAFLD). International Journal for Vitamin and Nutrition Research. 2019;89(5-6):293-302.

113. Ghaffari S, Navabzadeh M, Ziaee M, Ghobadi A, Ghods R, Hashem-Dabaghian F. A Randomized, Triple-Blind, Placebo-Controlled, Add-On Clinical Trial to Evaluate the Efficacy of Emblica officinalis in Uncontrolled Hypertension. Evidence-based Complementary and Alternative Medicine. 2020;2020.

114. Gharakhanlou BJ, Bonab SB, Amaghani A, Shiri-Shahsavar MR. Curcumin supplementation combined with high intensity interval training modulates serum irisin and lipid profile in obese women: "A randomized double-blind clinical trial". Contemporary Clinical Trials Communications. 2025;44.

115. Ghasemiadl M, Ghasemi S, Soleimani A, Esfahani MM, Azadbakht J, Gilasi HR, et al. The Effects of Curcumin Administration on Carotid Intima-Media Thickness and Pulse Wave Velocity in Diabetic Hemodialysis Patients: A Randomized, Double-Blinded, Placebo-Controlled Trial. International Journal of Preventive Medicine. 2024;15(1):3.

116. Ghazaee H, Sahebkar A, Mazloum Khorasani Z, Emadzadeh M. The effect of curcumin and piperine co-supplementation on glycemic indices in prediabetes: A triple-blind randomized controlled trial. Avicenna Journal of Phytomedicine. 2026;16(3):144-155.

117. Gheflati A, Adelnia E, Nadjarzadeh A. The clinical effects of purslane (Portulaca oleracea) seeds on metabolic profiles in patients with nonalcoholic fatty liver disease: A randomized controlled clinical trial. Phytotherapy Research. 2019;33(5):1501-1509.

118. Gheldof N, Francey C, Rytz A, Egli L, Delodder F, Bovetto L, et al. Effect of Different Nutritional Supplements on Glucose Response of Complete Meals in Two Crossover Studies. Nutrients. 2022;14(13).

119. Ghoreishi AS, Chatrnour G, Mahmoodi M. The effect of sesame seeds on fast blood sugar, haemoglobin A1C, liver enzymes and lipid profile in patients with type 2 diabetes: a randomised clinical trial. Family Medicine and Primary Care Review. 2022;24(3):207-211.

120. Ghoreishi PS, Shams M, Nimrouzi M, Zarshenas MM, Lankarani KB, Fallahzadeh Abarghooei E, et al. The Effects of Ginger (Zingiber Officinale Roscoe) on Non-Alcoholic Fatty Liver Disease in Patients with Type 2 Diabetes Mellitus: A Randomized Double-Blinded Placebo-Controlled Clinical Trial. Journal of dietary supplements. 2024;21(3):294-312.

121. Giannoulaki P, Kotzakioulafi E, Nakas A, Kontoninas Z, Karlafti E, Evripidou P, et al. Effect of Crocus sativus Extract Supplementation in the Metabolic Control of People with Diabetes Mellitus Type 1: A Double-Blind Randomized Placebo-Controlled Trial. Nutrients. 2024;16(13).

122. Gomonova VP, Baranovsk AYu, Sakeian IS. Honey preparation in complex therapy of metabolically associated fatty liver disease. [Russian]. Experimental and Clinical Gastroenterology. 2024;2024(10):195-205.

123. Gourdomichali T, Papakonstantinou E. Short-term effects of six Greek honey varieties on glycemic response: a randomized clinical trial in healthy subjects. European Journal of Clinical Nutrition. 2018;72(12):1709-1716.

124. Gout B, Bourges C, Paineau-Dubreuil S. Satiereal, a Crocus sativus L extract, reduces snacking and increases satiety in a randomized placebo-controlled study of mildly overweight, healthy women. Nutrition Research. 2010;30(5):305-313.

125. Gupta Jain S, Puri S, Misra A, Gulati S, Mani K. Effect of oral cinnamon intervention on metabolic profile and body composition of Asian Indians with metabolic syndrome: A randomized double -blind control trial. Lipids in Health and Disease. 2017;16(1).

126. Hajiaghamohammadi AA, Ziaee A, Samimi R. The efficacy of licorice root extract in decreasing transaminase activities in non-alcoholic fatty liver disease: A randomized controlled clinical trial. Phytotherapy Research. 2012;26(9):1381-1384.

127. Hajimonfarednejad M, Nimrouzi M, Heydari M, Zarshenas MM, Raee MJ, Jahromi BN. Insulin resistance improvement by cinnamon powder in polycystic ovary syndrome: A randomized double-blind placebo controlled clinical trial. Phytotherapy Research. 2018;32(2):276-283.

128. Hajimoosayi F, Jahanian Sadatmahalleh S, Kazemnejad A, Pirjani R. Effect of ginger on the blood glucose level of women with gestational diabetes mellitus (GDM) with impaired glucose tolerance test (GTT): a randomized double-blind placebo-controlled trial. BMC Complement Med Ther. 2020;20(1):116.

129. Hajleh MNA, Al-Dujaili EAS. Effects of turmeric concentrate on cardiovascular risk factors and exercise-induced oxidative stress in healthy volunteers; An exploratory study. Advanced Pharmaceutical Bulletin. 2023;13(3):601-610.

130. Hasanzade F, Toliat M, Emami SA, Emamimoghaadam Z. The Effect of Cinnamon on Glucose of Type II Diabetes Patients. J Tradit Complement Med. 2013;3(3):171-4.

131. Hashimoto M, Tanabe Y, Hossain S, Matsuzaki K, Ohno M, Kato S, et al. Intake of Alpha-Linolenic Acid-Rich Perilla frutescens Leaf Powder Decreases Home Blood Pressure and Serum Oxidized Low-Density Lipoprotein in Japanese Adults. Molecules. 2020;25(9).

132. Hassan OMS, Di Folco U, Nardone MR, Tubili F, Tubili C. Fiber enrichment of pasta: metabolic effects and diet adherence in obese subjects. Mediterranean Journal of Nutrition and Metabolism. 2020;13(1):53-62.

133. Hassanzadeh Delui M, Fatehi H, Manavifar M, Amini M, Ghayour-Mobarhan M, Zahedi M, et al. The effects of Panax ginseng on lipid profile, pro-oxidant: Antioxidant status and high-sensitivity C reactive protein levels in hyperlipidemic patients in Iran. International Journal of Preventive Medicine. 2013;4(9):1045-1051.

134. He Y, Chen X, Li Y, Liang Y, Hong T, Yang J, et al. Curcumin supplementation alleviates hepatic fat content associated with modulation of gut microbiota-dependent bile acid metabolism in patients with nonalcoholic simple fatty liver disease: a randomized controlled trial. American Journal of Clinical Nutrition. 2024;120(1):66-79.

135. Helli B, Gerami H, Kavianpour M, Heybar H, Hosseini SK, Haghighian HK. Curcumin Nanomicelle Improves Lipid Profile, Stress Oxidative Factors and Inflammatory Markers in Patients Undergoing Coronary Elective Angioplasty; A Randomized Clinical Trial. Endocr Metab Immune Disord Drug Targets. 2021;21(11):2090-2098.

136. Hellmann PH, Bagger JI, Carlander KR, Forman J, Chabanova E, Svenningsen JS, et al. The effect of curcumin on hepatic fat content in individuals with obesity. Diabetes, Obesity and Metabolism. 2022;24(11):2192-2202.

137. Hellmann PH, Bagger JI, Carlander KR, Hansen KB, Forman JL, Storling J, et al. No effect of the turmeric root phenol curcumin on prednisolone-induced glucometabolic perturbations in men with overweight or obesity. Endocrine Connections. 2023;12(4).

138. Hendre AS, Sontakke AV, Patil SR, Phatak RS. Effect of cinnamon supplementation on fasting blood glucose and insulin resistance in patients with type 2 diabetes. Pravara Medical Review. 2019;11(2):4-8.

139. Heshmati J, Moini A, Sepidarkish M, Morvaridzadeh M, Salehi M, Palmowski A, et al. Effects of curcumin supplementation on blood glucose, insulin resistance and androgens in patients with polycystic ovary syndrome: A randomized double-blind placebo-controlled clinical trial. Phytomedicine. 2021;80.

140. Hlebowicz J, Darwiche G, Bjorgell O, Almer LO. Effect of cinnamon on postprandial blood glucose, gastric emptying, and satiety in healthy subjects. American Journal of Clinical Nutrition. 2007;85(6):1552-1556.

141. Hlebowicz J, Hlebowicz A, Lindstedt S, Bjorgell O, Hoglund P, Hoist JJ, et al. Effects of 1 and 3 g cinnamon on gastric emptying, satiety, and postprandial blood glucose, insulin, glucose-dependent insulinotropic polypeptide, glucagon-like peptide 1, and ghrelin concentrations in healthy subjects. American Journal of Clinical Nutrition. 2009;89(3):815-821.

142. Hodaei H, Adibian M, Nikpayam O, Hedayati M, Sohrab G. The effect of curcumin supplementation on anthropometric indices, insulin resistance and oxidative stress in patients with type 2 diabetes: A randomized, double-blind clinical trial. Diabetology and Metabolic Syndrome. 2019;11(1).

143. Holubarsch CJF, Colucci WS, Meinertz T, Gaus W, Tendera M. The efficacy and safety of Crataegus extract WS 1442 in patients with heart failure: The SPICE trial. European Journal of Heart Failure. 2008;10(12):1255-1263.

144. Hong M, Lee YH, Kim S, Suk KT, Bang CS, Yoon JH, et al. Anti-inflammatory and antifatigue effect of Korean red ginseng in patients with nonalcoholic fatty liver diseaseq. Journal of Ginseng Research. 2016;40(3):203-210.

145. Hooshmand-Moghadam B, Eskandari M, Shabkhiz F, Mojtahedi S, Mahmoudi N. Saffron (Crocus sativus L.) in combination with resistance training reduced blood pressure in the elderly hypertensive men: A randomized controlled trial. British Journal of Clinical Pharmacology. 2021;87(8):3255-3267.

146. Hooshmandi H, Ghadiri-Anari A, Ranjbar AM, Fallahzadeh H, Hosseinzadeh M, Nadjarzadeh A. Effects of licorice extract in combination with a low-calorie diet on obesity indices, glycemic indices, and lipid profiles in overweight/obese women with polycystic ovary syndrome (PCOS): a randomized, double-blind, placebo-controlled trial. Journal of Ovarian Research. 2024;17(1).

147. Hosseini H, Bagherniya M, Sahebkar A, Iraj B, Majeed M, Askari G. The effect of curcumin-piperine supplementation on lipid profile, glycemic index, inflammation, and blood pressure in patients with type 2 diabetes mellitus and hypertriglyceridemia. Phytotherapy Research. 2024;38(11):5150-5161.

148. Hosseini Marnani E, et al. Effect of fennel supplementation along with high-protein, low-carbohydrate weight-loss diet on insulin resistance and percentage of fat and muscle mass in overweight/obese women with polycystic ovary syndrome. J Funct Foods. 2020;67:103848.

149. Hosseini SA, Ehsanpour A, Asgari M, Malihi R. Evaluation of standardized ginseng extract Panax (G115) effect on fasting blood glucose levels, glycated hemoglobin and lipid profile in patients with diabetes type 2. Jundishapur J Chronic Dis Care. 2014;3(2):e93793.

150. Hwang SH, Li HM, Lim SS, Wang Z, Hong JS, Huang B. Evaluation of a Standardized Extract from Morus alba against α-Glucosidase Inhibitory Effect and Postprandial Antihyperglycemic in Patients with Impaired Glucose Tolerance: A Randomized Double-Blind Clinical Trial. Evid Based Complement Alternat Med. 2016;2016:8983232.

151. Imani H, Tabibi H, Najafi I, Atabak S, Hedayati M, Rahmani L. Effects of ginger on serum glucose, advanced glycation end products, and inflammation in peritoneal dialysis patients. Nutrition. 2015;31(5):703-707.

152. Irannejad Niri Z, Shidfar F, Jabbari M, Zarrati M, Hosseini A, Malek M, et al. The effect of dried Ziziphus vulgaris on glycemic control, lipid profile, Apo-proteins and hs-CRP in patients with type 2 diabetes mellitus: A randomized controlled clinical trial. Journal of food biochemistry. 2021;45(3):e13193.

153. Jaafarinia A, Kafami B, Sahebnasagh A, Saghafi F. Evaluation of therapeutic effects of crocin in attenuating the progression of diabetic nephropathy: a preliminary randomized triple-blind placebo-controlled trial. BMC Complementary Medicine and Therapies. 2022;22(1).

154. Jafar N, Qalbi FN, Thaha RM, Hadju V, Hidayanti H, Salam A, et al. Effect of cinnamomum burmannii stew on glucose fasting blood levels in adult prediabetes in Makassar. Open Access Macedonian Journal of Medical Sciences. 2020;8(T2):71-74.

155. Jamilian M, Foroozanfard F, Kavossian E, Aghadavod E, Shafabakhsh R, Hoseini A, et al. Effects of curcumin on body weight, glycemic control and serum lipids in women with polycystic ovary syndrome: A randomized, double-blind, placebo-controlled trial. Clinical Nutrition ESPEN. 2020;36:128-133.

156. jarhahzadeh M, Alavinejad P, Farsi F, Husain D, Rezazadeh A. The effect of turmeric on lipid profile, malondialdehyde, liver echogenicity and enzymes among patients with nonalcoholic fatty liver disease: a randomized double blind clinical trial. Diabetology and Metabolic Syndrome. 2021;13(1).

157. Javandoost A, Afshari A, Saberi-Karimian M, Sahebkar A, Safarian H, Moammeri M, et al. The effects of curcumin and a modified curcumin formulation on serum Cholesteryl Ester Transfer Protein concentrations in patients with metabolic syndrome: A randomized, placebo-controlled clinical trial. Avicenna Journal of Phytomedicine. 2018;8(4):330-337.

158. Jayadi Y, Thaha AR, Hadju V, Bukhari A, Dewi NU, Bohari . The potential of Indonesian honey to change the lipid profiles of individuals with central obesity. Pakistan Journal of Nutrition. 2019;18(6):508-513.

159. Jazayeri-Tehrani SA, Rezayat SM, Mansouri S, Qorbani M, Alavian SM, Daneshi-Maskooni M, et al. Nano-curcumin improves glucose indices, lipids, inflammation, and Nesfatin in overweight and obese patients with non-alcoholic fatty liver disease (NAFLD): A double-blind randomized placebo-controlled clinical trial. Nutrition and Metabolism. 2019;16(1).

160. Jeong Y, Lee SH, Shim SL, Jang KH, Kim JH. Efficacy and safety of red ginseng extract powder (KGC05pg) in achieving glycemic control in prediabetic Korean adults A 12-week, single-center, randomized, double-blind, parallel-group, placebo-controlled study. Medicine. 2024;103(52):e41130.

161. Jia W, Li Y, Wan J, Cui X, Lu J, Liu J, et al. Effects of Xuezhitong in Patients with Hypertriglyceridemia: a Multicentre, Randomized, Double-Blind, Double Simulation, Positive Drug and Placebo Parallel Control Study. Cardiovascular Drugs and Therapy. 2020;34(4):525-534.

162. Jovanovski E, Bateman EA, Bhardwaj J, Fairgrieve C, Mucalo I, Jenkins AL, et al. Effect of Rg3-enriched Korean red ginseng (Panax ginseng) on arterial stiffness and blood pressure in healthy individuals: A randomized controlled trial. Journal of the American Society of Hypertension. 2014;8(8):537-541.

163. Jovanovski E, Jenkins A, Dias AG, Peeva V, Sievenpiper J, Arnason JT, et al. Effects of korean red ginseng (Panax ginseng C.A. Mayer) and its isolated ginsenosides and polysaccharides on arterial stiffness in healthy individuals. American Journal of Hypertension. 2010;23(5):469-472.

164. Jovanovski E, Peeva V, Sievenpiper JL, Jenkins AL, Desouza L, Rahelic D, et al. Modulation of endothelial function by korean red ginseng (Panax ginseng C.A. meyer) and its components in healthy individuals: A randomized controlled trial. Cardiovascular Therapeutics. 2014;32(4):163-169.

165. Jovanovski E, Smircic-Duvnjak L, Komishon A, Au-Yeung FR, Sievenpiper JL, Zurbau A, et al. Effect of coadministration of enriched Korean Red Ginseng (Panax ginseng) and American ginseng (Panax quinquefolius L) on cardiometabolic outcomes in type-2 diabetes: A randomized controlled trial. Journal of Ginseng Research. 2021;45(5):546-554.

166. Jozefczuk J, Malikowska K, Glapa A, Stawinska-Witoszynska B, Nowak JK, Bajerska J, et al. Mulberry leaf extract decreases digestion and absorption of starch in healthy subjects-A randomized, placebo-controlled, crossover study. Advances in Medical Sciences. 2017;62(2):302-306.

167. Juesas A, Saez-Berlanga A, Babiloni-Lopez C, Martin EG, Garrigues-Pelufo L, Ferri-Caruana A, et al. Effects of Accentuated Eccentric and Maximal Strength High-Resistance Training Programs with or Without a Curcumin-Based Formulation Supplement on Body Composition, Blood Pressure, and Metabolic Parameters in Older Adults. Diseases. 2025;13(2).

168. Kadhim HM. Antiinflammatory and antihyperlipidemic effect of adjuvant cinnamon in type 2 diabetic patients. Int J Pharm Sci Rev Res. 2016;41(1):88-93.

169. Kang J, Lee N, Ahn Y, Lee H. Study on improving blood flow with Korean red ginseng substances using digital infrared thermal imaging and Doppler sonography: randomized, double blind, placebo-controlled clinical trial with parallel design. Journal of Traditional Chinese Medicine. 2013;33(1):39-45.

170. Kang YM, Lee BJ, Kim JI, Nam BH, Cha JY, Kim YM, et al. Fermented Sea Tangle Attenuates Oxidative Stress in Individuals with a High Level of γ-Glutamyltransferase: a Randomized, Double-Blind, and Placebo-controlled Clinical Study. FOOD SCIENCE AND BIOTECHNOLOGY. 2014;23(3):937-941.

171. Kapoor MP, Suzuki K, Derek T, Ozeki M, Okubo T. Clinical evaluation of Emblica Officinalis Gatertn (Amla) in healthy human subjects: Health benefits and safety results from a randomized, double-blind, crossover placebo-controlled study. Contemporary Clinical Trials Communications. 2020;17.

172. Karandish M, Mozaffari-khosravi H, Mohammadi SM, Cheraghian B, Azhdari M. The effect of curcumin and zinc co-supplementation on glycemic parameters in overweight or obese prediabetic subjects: A phase 2 randomized, placebo-controlled trial with a multi-arm, parallel-group design. Phytotherapy Research. 2021;35(8):4377-4387.

173. Karimi N, Roshan VD, Bayatiyani ZF. Individually and combined water-based exercise with ginger supplement, on systemic inflammation and metabolic syndrome indices, among the obese women with breast neoplasms. International Journal of Cancer Management. 2015;8(6).

174. Kavita MB, Mallika K, Poornima B. A clinical study on effect of Amalaki (Indian Gooseberry) as food supplement in Dyslipidemia. International Journal of Research in Ayurveda and Pharmacy. 2016;7(4):59-64.

175. Kermani T, Kazemi T, Molki S, Ilkhani K, Sharifzadeh G, Rajabi O. The efficacy of crocin of saffron (Crocus sativus L.) on the components of metabolic syndrome: A randomized controlled clinical trial. Journal of Research in Pharmacy Practice. 2017;6(4):228-232.

176. Kermani T, Zebarjadi M, Mehrad-Majd H, Mirhafez SR, Shemshian M, Ghasemi F, et al. Anti-inflammatory effect of crocus sativus on serum cytokine levels in subjects with metabolic syndrome: A randomized, double-blind, placebo-controlled trial. Current Clinical Pharmacology. 2017;12(2):122-126.

177. Khan AA, Begum W. Efficacy of Darchini in the management of polycystic ovarian syndrome: A randomized clinical study. Journal of Herbal Medicine. 2019;15.

178. Khan S, Arif M, Laraib H, Naqvi SN, Shah OA, Farooq U, et al. The effect of turmeric and black pepper powder incorporated in breakfast on postprandial glycemia, appetite, palatability, and gastrointestinal well-being in normal-weight adults. Food Sci Nutr. 2024;12(4):2846-2854.

179. Khandouzi N, Shidfar F, Rajab A, Rahideh T, Hosseini P, Taheri MM. The effects of ginger on fasting blood sugar, hemoglobin A1c, apolipoprotein B, apolipoprotein A-I and malondialdehyde in type 2 diabetic patients. Iranian Journal of Pharmaceutical Research. 2015;14(1):131-140.

180. Khattab MNM, El-Hadidy HA, Abdelhady RH, Abo-El-Ros MAM. Response of metabolic syndrome components to honey intake as an add-on therapy to free walking exercise in children: a randomized controlled trial. Family Medicine and Primary Care Review. 2026;28(1):41-47.

181. Kim HR, Paulrayer A, Kwon YG, Ryu DG, Baek DG, Geum JH, et al. Acute effects of Amomum villosum Lour. fruit extract on postprandial glycemia and insulin secretion: A single-blind, placebo-controlled, crossover study in healthy subjects. Saudi J Biol Sci. 2020;27(11):2968-2971.

182. Kim JY, Ok HM, Kim J, Park SW, Kwon SW, Kwon O. Mulberry leaf extract improves postprandial glucose response in prediabetic subjects: A randomized, double-blind placebo-controlled trial. Journal of Medicinal Food. 2015;18(3):306-313.

183. Kim YJ, Kwon EY, Kim JW, Lee Y, Ryu R, Yun J, et al. Intervention study on the efficacy and safety of Platycodon grandiflorus ethanol extract in overweight or moderately obese adults: A single-center, randomized, double-blind, placebo-controlled trial. Nutrients. 2019;11(10).

184. Kitano-Okada T, Nagata R, Han KH, Mikami N, Satoh K, Nishihira J, et al. Safety and efficacy of adzuki bean extract in subjects with moderate to high LDL-C: a randomized trial. Bioscience, biotechnology, and biochemistry. 2019;83(5):933-941.

185. Klupp NL, Kiat H, Bensoussan A, Steiner GZ, Chang DH. A double-blind, randomised, placebo-controlled trial of Ganoderma lucidum for the treatment of cardiovascular risk factors of metabolic syndrome. Scientific reports. 2016;6:29540.

186. Kocher A, Bohnert L, Schiborr C, Frank J. Highly bioavailable micellar curcuminoids accumulate in blood, are safe and do not reduce blood lipids and inflammation markers in moderately hyperlipidemic individuals. Mol Nutr Food Res. 2016;60(7):1555-63.

187. Kotanidou EP, Tsinopoulou VR, Giza S, Ntouma S, Angeli C, Chatziandreou M, et al. The Effect of Saffron Kozanis (Crocus sativus L.) Supplementation on Weight Management, Glycemic Markers and Lipid Profile in Adolescents with Obesity: A Double-Blinded Randomized Placebo-Controlled Trial. Children. 2023;10(11).

188. Kuwahara M, Kim HK, Ozaki M, Nanba T, Chijiki H, Fukazawa M, et al. Consumption of biscuits with a beverage of mulberry or barley leaves in the afternoon prevents dinner-induced high, but not low, increases in blood glucose among young adults. Nutrients. 2020;12(6).

189. Kwok MK, Leung GM, Xu L, Tse HF, Lam TH, Schooling CM. Effect of puerarin supplementation on cardiovascular disease risk factors: A randomized, double-blind, placebo-controlled, 2-way crossover trial. Biomedicine and Pharmacotherapy. 2022;153.

190. Kwon DH, Bose S, Song MY, Lee MJ, Lim CY, Kwon BS, et al. Efficacy of Korean Red Ginseng by Single Nucleotide Polymorphism in Obese Women: Randomized, Double-blind, Placebo-controlled Trial. J Ginseng Res. 2012;36(2):176-89.

191. Kwon JE, Lee YG, Kim I, Bae J, Kang SC, Baek HI. Efficacy and Safety of Steamed Ginger Extract for Body Weight and Body Fat Reduction in Overweight Adults: A Randomized, Double-Blind, Placebo-Controlled Clinical Trial. Nutrients. 2026;18(2).

192. Kwon YJ, Lee GM, Liu KH, Jung DH. Effect of Korean red ginseng on plasma ceramide levels in postmenopausal women with hypercholesterolemia: A pilot randomized controlled trial. Metabolites. 2021;11(7).

193. Lamichhane G, Godsey TJ, Liu J, Franks R, Zhang G, Emerson SR, et al. Twelve-Week Curcumin Supplementation Improves Glucose Homeostasis and Gut Health in Prediabetic Older Adults: A Pilot, Double-Blind, Placebo-Controlled Trial. Nutrients. 2025;17(13).

194. Larmo PS, Kangas AJ, Soininen P, Lehtonen HM, Suomela JP, Yang B, et al. Effects of sea buckthorn and bilberry on serum metabolites differ according to baseline metabolic profiles in overweight women: A randomized crossover trial. American Journal of Clinical Nutrition. 2013;98(4):941-951.

195. Larmo PS, Yang B, Hurme SAM, Alin JA, Kallio HP, Salminen EK, et al. Effect of a low dose of sea buckthorn berries on circulating concentrations of cholesterol, triacylglycerols, and flavonols in healthy adults. European Journal of Nutrition. 2009;48(5):277-282.

196. Larson-Meyer DE, Willis KS, Willis LM, Austin KJ, Hart AM, Breton AB, et al. Effect of honey versus sucrose on appetite, appetite-regulating hormones, and postmeal thermogenesis. Journal of the American College of Nutrition. 2010;29(5):482-493.

197. Lee JH, Kim HR, Antonisamy P, Kim YS, Ryu DG, Lee G, et al. Efficacy and safety of Amomum villosum extracts in obese adults: A randomized, double-blind, placebo-controlled trial. JOURNAL OF KING SAUD UNIVERSITY SCIENCE. 2023;35(3).

198. Lee JY, Liao WL, Liu YH, Kuo CL, Lung FW, Hsieh CL. Oral administration of processed Cassia obtusifolia L. seed powder May reduce body weight and cholesterol in overweight patients with schizophrenia: A 36-week randomized, double-blind, controlled trial of high and low doses. Journal of Ethnopharmacology. 2022;292.

199. Lee KJ, Lee SY, Ji GE. Diabetes-ameliorating effects of fermented red ginseng and causal effects on hormonal interactions: Testing the hypothesis by multiple group path analysis. Journal of Medicinal Food. 2013;16(5):383-395.

200. Li NY, Yu H, Li XL, Wang QY, Zhang XW, Ma RX, et al. Astragalus Membranaceus Improving Asymptomatic Left Ventricular Diastolic Dysfunction in Postmenopausal Hypertensive Women with Metabolic Syndrome: A Prospective, Open-Labeled, Randomized Controlled Trial. Chinese Medical Journal. 2018;131(5):516-526.

201. Lightowler H, Thondre S, Holz A, Theis S. Replacement of glycaemic carbohydrates by inulin-type fructans from chicory (oligofructose, inulin) reduces the postprandial blood glucose and insulin response to foods: report of two double-blind, randomized, controlled trials. European Journal of Nutrition. 2018;57(3):1259-1268.

202. Lin S, Hao G, Lai D, Tian Y, Long M, Lai F, et al. Effect of Oyster Meat Preload on Postmeal Glycemic Control in Healthy Young Adults. Journal of the American College of Nutrition. 2020;39(6):511-517.

203. Linderborg KM, Lehtonen HM, Jarvinen R, Viitanen M, Kallio H. The fibres and polyphenols in sea buckthorn (Hippophae rhamnoides) extraction residues delay postprandial lipemia. Int J Food Sci Nutr. 2012;63(4):483-490.

204. Lira Neto JCG, Araujo MFM, Araujo AVEC, Figueira JNR, Maranhao TA, Damasceno MMC. Effectiveness of cinnamon in the reduction of lipid levels in people with diabetes: a randomized clinical trial. Revista gaucha de enfermagem. 2023;44:e20230051.

205. Lira Neto JCG, Araujo MFM, Penha JCD, Bezerra MAR, Fernandes BKC, Maranhao TA, et al. Efficacy of cinnamon in reducing anthropometric measurements in individuals with type 2 diabetes: a clinical trial. Revista brasileira de enfermagem. 2025;78(3):e20240380.

206. Lira Neto JCG, Damasceno MMC, Ciol MA, de Freitas RWJF, de Araujo MFM, Teixeira CRS, et al. Efficacy of Cinnamon as an Adjuvant in Reducing the Glycemic Biomarkers of Type 2 Diabetes Mellitus: A Three-Month, Randomized, Triple-Blind, Placebo-Controlled Clinical Trial. Journal of the American Nutrition Association. 2022;41(3):266-274.

207. Liu X, Li M, Song W, Zhang Y, Bao W, Liu C, et al. Short-Term, Cycle-Synchronized Adjunctive Therapy With Crocus Total Glucosides Tablets for Cardiac Protection Against Cancer Therapy-Related Cardiac Dysfunction in Breast Cancer Patients: A Randomized, Double-Blind, Placebo-Controlled Trial. MedComm (2020). 2026;7(6):e70780.

208. Liu Y, Wang Q, Li S, Yue Y, Ma Y, Ren G. Convenient food made of extruded adzuki bean attenuates inflammation and improves glycemic control in patients with type 2 diabetes: A randomized controlled trial. Therapeutics and Clinical Risk Management. 2018;14:871-884.

209. Lown M, Fuller R, Lightowler H, Fraser A, Gallagher A, Stuart B, et al. Mulberry-extract improves glucose tolerance and decreases insulin concentrations in normoglycaemic adults: Results of a randomised double-blind placebo-controlled study. PLoS ONE. 2017;12(2).

210. Lu T, Sheng H, Wu J, Cheng Y, Zhu J, Chen Y. Cinnamon extract improves fasting blood glucose and glycosylated hemoglobin level in Chinese patients with type 2 diabetes. Nutrition Research. 2012;32(6):408-412.

211. Ma SW, Benzie IFF, Chu TTW, Fok BSP, Tomlinson B, Critchley LAH. Effect of Panax ginseng supplementation on biomarkers of glucose tolerance, antioxidant status and oxidative stress in type 2 diabetic subjects: Results of a placebo-controlled human intervention trial. Diabetes, Obesity and Metabolism. 2008;10(11):1125-1127.

212. Mafi A, Iraj B, Aarabi MH. The beneficial effects of crocin supplementation on metabolic and genetic parameters in type 2 diabetic patients under metformin treatment: A randomized double-blind controlled clinical trial. JOURNAL OF FUNCTIONAL FOODS. 2024;122.

213. Magistrelli A, Chezem JC. Effect of Ground Cinnamon on Postprandial Blood Glucose Concentration in Normal-Weight and Obese Adults. Journal of the Academy of Nutrition and Dietetics. 2012;112(11):1806-1809.

214. Mahluji S, Attari VE, Mobasseri M, Payahoo L, Ostadrahimi A, Golzari SE. Effects of ginger (Zingiber officinale) on plasma glucose level, HbA1c and insulin sensitivity in type 2 diabetic patients. International Journal of Food Sciences and Nutrition. 2013;64(6):682-686.

215. Maithili Karpaga Selvi N, Sridhar MG, Swaminathan RP, Sripradha R. Efficacy of Turmeric as Adjuvant Therapy in Type 2 Diabetic Patients. Indian Journal of Clinical Biochemistry. 2015;30(2):180-186.

216. Majeed M, Mundkur L, Paulose S, Nagabhushanam K. Novel Emblica officinalis extract containing β-glucogallin vs. metformin: a randomized, open-label, comparative efficacy study in newly diagnosed type 2 diabetes mellitus patients with dyslipidemia. Food Funct. 2022;13(18):9523-9531.

217. Majeed M, Nagabhushanam K, Devarajan TV, Saklecha S, Reddy SVK, Mundkur L. A minor metabolite from Curcuma longa effective against metabolic syndrome: results from a randomized, double-blind, placebo-controlled clinical study. Food & function. 2023;14(10):4722-4733.

218. Majid M, Younis MA, Naveed AK, Shah MU, Azeem Z, Tirmizi SH. Effects of natural honey on blood glucose and lipid profile in young healthy Pakistani males. Journal of Ayub Medical College, Abbottabad : JAMC. 2013;25(3-4):44-47.

219. Mandal A, Sharma SK, Yadav SRM, Mirza AA, Thakur MS, Jachak S, et al. Efficacy of Young Cinnamomum zeylanicum Blume Bark on Hyperglycemia and PTPase Activity in Type 2 Diabetes. Cureus. 2023;15(2):e35023.

220. Mang B, Wolters M, Schmitt B, Kelb K, Lichtinghagen R, Stichtenoth DO, et al. Effects of a cinnamon extract on plasma glucose, HbA, and serum lipids in diabetes mellitus type 2. Eur J Clin Invest. 2006;36(5):340-4.

221. Mansour MS, Ni YM, Roberts AL, Kelleman M, Roychoudhury A, St-Onge MP. Ginger consumption enhances the thermic effect of food and promotes feelings of satiety without affecting metabolic and hormonal parameters in overweight men: A pilot study. Metabolism: Clinical and Experimental. 2012;61(10):1347-1352.

222. Martinez-Rodriguez NL, Zurita-Cruz JN, Parra-Ortega I, Vilchis-Gil J, Villasis-Keever MA, Dominguez-Salgado JM. Cinnamon Supplementation Improves Uric Acid Levels in Adolescents with Obesity: Secondary Metabolic Outcomes from a Randomized Controlled Trial. Obesities. 2026;6(2).

223. Maruyama C, Araki R, Kawamura M, Kondo N, Kigawa M, Kawai Y, et al. Azuki bean juice lowers serum triglyceride concentrations in healthy young women. J Clin Biochem Nutr. 2008;43(1):19-25.

224. Mela DJ, Alssema M, Hiemstra H, Hoogenraad AR, Kadam T. Effect of Low-Dose Mulberry Fruit Extract on Postprandial Glucose and Insulin Responses: A Randomized Pilot Trial in Individuals with Type 2 Diabetes. Nutrients. 2024;16(14).

225. Mela DJ, Boers HM, Kadam T, Hiemstra H, Kalathil R, Seijen Ten Hoorn JWM. Effect of mulberry fruit extract on post-prandial glycaemic and insulinemic responses to different rice types: a randomised trial in healthy adults. British Journal of Nutrition. 2023;130(12):2088-2094.

226. Mela DJ, Cao XZ, Govindaiah S, Hiemstra H, Kalathil R, Lin L, et al. Dose-response efficacy of mulberry fruit extract for reducing post-prandial blood glucose and insulin responses: randomised trial evidence in healthy adults. British Journal of Nutrition. 2023;129(5):771-778.

227. Milajerdi A, Jazayeri S, Hashemzadeh N, Shirzadi E, Derakhshan Z, Djazayeri A, et al. The effect of saffron (Crocus sativus L.) hydroalcoholic extract on metabolic control in type 2 diabetes mellitus: A triple-blinded randomized clinical trial. Journal of Research in Medical Sciences. 2018;23(2).

228. Milkarizi N, Barghchi H, Belyani S, Bahari H, Rajabzade F, Ostad AN, et al. Effects of Portulaca oleracea (purslane) on liver function tests, metabolic profile, oxidative stress and inflammatory biomarkers in patients with non-alcoholic fatty liver disease: a randomized, double-blind clinical trial. Front Nutr. 2024;11:1371137.

229. Mirfeizi M, Mehdizadeh Tourzani Z, Mirfeizi SZ, Asghari Jafarabadi M, Rezvani HR, Afzali M. Controlling type 2 diabetes mellitus with herbal medicines: A triple-blind randomized clinical trial of efficacy and safety. Journal of Diabetes. 2016;8(5):647-656.

230. Mirhafez SR, Farimani AR, Dehhabe M, Bidkhori M, Hariri M, Motlagh Ghouchani BFN, et al. Effect of phytosomal curcumin on circulating levels of adiponectin and leptin in patients with non-alcoholic fatty liver disease: A randomized, double-blind, placebo-controlled clinical trial. Journal of Gastrointestinal and Liver Diseases. 2019;28(2):183-189.

231. Mirmiran P, Bahadoran Z, Golzarand M, Rajab A, Azizi F. Ardeh (Sesamum indicum) could improve serum triglycerides and atherogenic lipid parameters in type 2 diabetic patients: a randomized clinical trial. Arch Iran Med. 2013;16(11):652-656.

232. Mirmiranpour H, Huseini HF, Derakhshanian H, Khodaii Z, Tavakoli-Far B. Effects of probiotic, cinnamon, and synbiotic supplementation on glycemic control and antioxidant status in people with type 2 diabetes; a randomized, double-blind, placebo-controlled study. Journal of Diabetes and Metabolic Disorders. 2020;19(1):53-60.

233. Mirnasrollahi Parsa RS, Aryaeian N, Mokhtare M, Kavianipour F, Janani L, Agah S, et al. The Effects of Saffron (Crocus sativus L) Supplementation on Cardiovascular Risk Factors, Liver Steatosis, and Fibrosis in Patients With Non-alcoholic Fatty Liver Disease: A Randomised, Double-Blind, Placebo-Controlled Study. Journal of Herbal Medicine. 2024;45.

234. Mirzabeigi P, Mohammadpour AH, Salarifar M, Gholami K, Mojtahedzadeh M, Javadi MR. The effect of curcumin on some of traditional and non-traditional cardiovascular risk factors: A pilot randomized, double-blind, placebo-controlled trial. Iranian Journal of Pharmaceutical Research. 2015;14(2):479-486.

235. Mitchell CM, Davy BM, Ponder MA, McMillan RP, Hughes MD, Hulver MW, et al. Prebiotic Inulin Supplementation and Peripheral Insulin Sensitivity in adults at Elevated Risk for Type 2 Diabetes: A Pilot Randomized Controlled Trial. Nutrients. 2021;13(9).

236. Mobasseri M, Ostadrahimi A, Tajaddini A, Asghari S, Barati M, Akbarzadeh M, et al. Effects of saffron supplementation on glycemia and inflammation in patients with type 2 diabetes mellitus: A randomized double-blind, placebo-controlled clinical trial study. Diabetes and Metabolic Syndrome: Clinical Research and Reviews. 2020;14(4):527-534.

237. Moeini Badi F, Bathaie SZ, Borazjani F, Hosseini SA, Sheikhi MA, Shariful Islam SM, et al. The effect of crocetin (a saffron carotenoid) supplementation on antioxidant and inflammatory indexes and serum leptin concentration in patients with coronary artery disease. Food & function. 2025;16(11):4604-4614.

238. Mohamed M, Zagury RL, Bhaskaran K, Neutel J, Mohd Yusof BN, Mooney L, et al. A Randomized, Placebo-Controlled Crossover Study to Evaluate Postprandial Glucometabolic Effects of Mulberry Leaf Extract, Vitamin D, Chromium, and Fiber in People with Type 2 Diabetes. Diabetes Therapy. 2023;14(4):749-766.

239. Mohammadi A, Sahebkar A, Iranshahi M, Amini M, Khojasteh R, Ghayour-Mobarhan M, et al. Effects of supplementation with curcuminoids on dyslipidemia in obese patients: A randomized crossover trial. Phytotherapy Research. 2013;27(3):374-379.

240. Mohammadi F, Mohsenpour MA, Sohrabi Z, Niakousari M, Jeddi M, Hassanzadeh J, et al. The effects of powdered drinks enriched with curcumin and probiotics on lipid profile and atherogenic indices in patients with metabolic syndrome: A randomized, double-blinded, placebo-controlled clinical trial. Food Sci Nutr. 2024;12(2):1257-1267.

241. Mohammadrezaei A, Kavakeb A, Abbasalizad-Farhangi M, Mesgari-Abbasi M. Effects of hemp seed alone and combined with aerobic exercise on metabolic parameters, oxidative stress, and neurotrophic factors in young sedentary men. Journal of food biochemistry. 2022;46(12):e14417.

242. Mojtahedi S, Hooshmand-Moghadam B, Rosenkranz S, Shourideh Z, Amirshaghaghi F, Shabkhiz F. Improvement of inflammatory status following saffron (Crocus sativus L.) and resistance training in elderly hypertensive men: A randomized controlled trial. Experimental Gerontology. 2022;162.

243. Moncada MM, Bernardo MA, Silva ML, Jorge A, Pereira P, Brito J, et al. Effect of cinnamon powder addition to a Portuguese custard tart (Pastel de Nata) on healthy adults' postprandial glycemia. World Heart Journal. 2017;9(2):135-144.

244. Moohebati M, Yazdanoust S, Sahebkar A, Mazidi M, Sharghi-Shahri Z, Ferns G, et al. Investigation of the effect of short-term supplementation with curcuminoids on circulating small dense low-density lipoprotein concentrations in obese dyslipidemic subjects: A randomized double-blind placebo-controlled cross-over trial. ARYA Atherosclerosis. 2014;10(5).

245. Moradi B, Rahmati-Ahmadabad S, Farzanegi P, Helalizadeh M, Azarbayjani MA. Effects of non-linear resistance training and curcumin supplementation on the liver biochemical markers levels and structure in older women with non-alcoholic fatty liver disease. Journal of Bodywork and Movement Therapies. 2020;24(3):154-160.

246. Moraveji M, Sahebalzamani M, Safaee M. Effects of cinnamon on glucose control in glucose intolerant patients. LIFE SCIENCE JOURNAL-ACTA ZHENGZHOU UNIVERSITY OVERSEAS EDITION. 2013;10(1):438-440.

247. Moreira FD, Reis CEG, Gallassi AD, Moreira DC, Welker AF. Suppression of the postprandial hyperglycemia in patients with type 2 diabetes by a raw medicinal herb powder is weakened when consumed in ordinary hard gelatin capsules: A randomized crossover clinical trial. PLoS ONE. 2024;19(10).

248. Mortensen MW, Spagner C, Cuparencu C, Astrup A, Raben A, Dragsted LO. Sea buckthorn decreases and delays insulin response and improves glycaemic profile following a sucrose-containing berry meal: a randomised, controlled, crossover study of Danish sea buckthorn and strawberries in overweight and obese male subjects. European Journal of Nutrition. 2018;57(8):2827-2837.

249. Mozaffari-Khosravi H, Talaei B, Jalali BA, Najarzadeh A, Mozayan MR. The effect of ginger powder supplementation on insulin resistance and glycemic indices in patients with type 2 diabetes: A randomized, double-blind, placebo-controlled trial. Complementary Therapies in Medicine. 2014;22(1):9-16.

250. Mucalo I, Jovanovski E, Rahelic D, Bozikov V, Romic Z, Vuksan V. Effect of American ginseng (Panax quinquefolius L.) on arterial stiffness in subjects with type-2 diabetes and concomitant hypertension. Journal of Ethnopharmacology. 2013;150(1):148-153.

251. Muchimapura S, Thukhammee W, Phuthong S, Potue P, Khamseekaew J, Tong-Un T, et al. Mung Bean Functional Protein Enhances Endothelial Function via Antioxidant Activity and Inflammation Modulation in Middle-Aged Adults: A Randomized Double-Blind Trial. Foods. 2024;13(21).

252. Munshi R, Karande-Patil S, Kumbhar D, Deshmukh A, Hingorani L. A randomized, controlled, comparative, proof-of-concept study to evaluate the efficacy and safety of Nisha-Amalaki capsules in prediabetic patients for preventing progression to diabetes. Journal of Ayurveda and Integrative Medicine. 2023;14(6).

253. Munstedt K, Hoffmann S, Hauenschild A, Bulte M, Von Georgi R, Hackethal A. Effect of honey on serum cholesterol and lipid values. Journal of Medicinal Food. 2009;12(3):624-628.

254. Munstedt K, Sheybani B, Hauenschild A, Bruggmann D, Bretzel RG, Winter D. Effects of basswood honey, honey-comparable glucose-fructose solution, and oral glucose tolerance test solution on serum insulin, glucose, and C-peptide concentrations in healthy subjects. Journal of Medicinal Food. 2008;11(3):424-428.

255. Mushtaq R, Mushtaq R, Khan ZT. Effects of Natural Honey on Lipid Profile and Body Weight in Normal Weight and Obese Adults: A Randomized Clinical Trial. PAKISTAN JOURNAL OF ZOOLOGY. 2011;43(1):161-169.

256. Musso G, Pinach S, Mariano F, Saba F, De Michieli F, Framarin L, et al. Effect of phospholipid curcumin Meriva® on liver histology and kidney disease in nonalcoholic steatohepatitis a randomized, double-blind, placebo-controlled trial. Hepatology. 2025;81(2).

257. Muthukuda D, de Silva CK, Ajanthan S, Wijesinghe N, Dahanayaka A, Pathmeswaran A. Effects of Cinnamomum zeylanicum (Ceylon cinnamon) extract on lipid profile, glucose levels and its safety in adults: A randomized, double-blind, controlled trial. PLoS ONE. 2025;20(1).

258. Nada EM, El-Gharbawy NM, Abbas H, Werida RH. Effect of adding fenofibrate versus curcumin to glimepiride in patients with type 2 diabetes: a randomized controlled trial. BMC Pharmacology and Toxicology. 2025;26(1).

259. Neta JFF, Veras VS, Sousa DF, Cunha MDCDSO, Queiroz MVO, Neto JCGL, et al. Effectiveness of the piperine-supplemented Curcuma longa L. in metabolic control of patients with type 2 diabetes: a randomised double-blind placebo-controlled clinical trial. International Journal of Food Sciences and Nutrition. 2021;72(7):968-977.

260. Niederseer D, Ledl-Kurkowski E, Kvita K, Funk P, Niebauer J. Safety and Effects of Crataegus Extract Ws 1442 and Nordic Walking on Lipid Profile and Endothelial Function: A Randomized, Partially Blinded Pilot Study in Overweight Volunteers. Acta clinica Croatica. 2019;58(4):604-614.

261. Nieman DC, Cialdella-Kam L, Knab AM, Shanely RA. Influence of red pepper spice and turmeric on inflammation and oxidative stress biomarkers in overweight females: a metabolomics approach. Plant Foods Hum Nutr. 2012;67(4):415-421.

262. Nilsson A. Preload time-dependent effects of Panax ginseng on postprandial glucose tolerance. A randomized controlled study in healthy middle-aged participants. Front Nutr. 2026;13:1759162.

263. Nishimura M, Ohkawara T, Kanayama T, Kitagawa K, Nishimura H, Nishihira J. Effects of the extract from roasted chicory (Cichorium intybus L.) root containing inulin-type fructans on blood glucose, lipid metabolism, and fecal properties. J Tradit Complement Med. 2015;5(3):161-7.

264. Nishiumi S, Izumi Y, Kobayashi T, Yoshida M. A Pilot Study: Effects of Kombu Intake on Lifestyle-related Diseases -Possibility that Kombu Intake is Effective in Individuals with Abnormally High Serum Triglyceride Levels. FOOD SCIENCE AND TECHNOLOGY RESEARCH. 2019;25(6):827-834.

265. Nouri-Vaskeh M, Hashemi P, Hataminia N, Yazdani Y, Nasirian M, Alizadeh L. The impact of piperine on the metabolic conditions of patients with NAFLD and early cirrhosis: a randomized double-blind controlled trial. Scientific reports. 2024;14(1):1053.

266. Oh MR, Park SH, Kim SY, Back HI, Kim MG, Jeon JY, et al. Postprandial glucose-lowering effects of fermented red ginseng in subjects with impaired fasting glucose or type 2 diabetes: A randomized, double-blind, placebo-controlled clinical trial. BMC Complementary and Alternative Medicine. 2014;14.

267. Oliver JM, Stoner L, Rowlands DS, Caldwell AR, Sanders E, Kreutzer A, et al. Novel Form of Curcumin Improves Endothelial Function in Young, Healthy Individuals: A Double-Blind Placebo Controlled Study. J Nutr Metab. 2016;2016:1089653.

268. Omary L, Canfora EE, Puhlmann ML, Gavriilidou A, Rijnaarts I, Holst JJ, et al. Intrinsic chicory root fibers modulate colonic microbial butyrate-producing pathways and improve insulin sensitivity in individuals with obesity. Cell Reports Medicine. 2025;6(7).

269. Panahi Y, Khalili N, Hosseini MS, Abbasinazari M, Sahebkar A. Lipid-modifying effects of adjunctive therapy with curcuminoids-piperine combination in patients with metabolic syndrome: Results of a randomized controlled trial. Complementary Therapies in Medicine. 2014;22(5):851-857.

270. Panahi Y, Khalili N, Sahebi E, Namazi S, Simental-Mendia LE, Majeed M, et al. Effects of Curcuminoids Plus Piperine on Glycemic, Hepatic and Inflammatory Biomarkers in Patients with Type 2 Diabetes Mellitus: A Randomized Double-Blind Placebo-Controlled Trial. Drug Research. 2018;68(7):403-409.

271. Panahi Y, Kianpour P, Mohtashami R, Jafari R, Simental-Mendia LE, Sahebkar A. Curcumin Lowers Serum Lipids and Uric Acid in Subjects with Nonalcoholic Fatty Liver Disease: A Randomized Controlled Trial. Journal of Cardiovascular Pharmacology. 2016;68(3):223-229.

272. Panahi Y, Valizadegan G, Ahamdi N, Ganjali S, Majeed M, Sahebkar A. Curcuminoids plus piperine improve nonalcoholic fatty liver disease: A clinical trial. Journal of Cellular Biochemistry. 2019;120(9):15989-15996.

273. Papoli M, Pishdad S, Nadjarzadeh A, Hosseinzadeh M. Effects of consuming purslane seed powder on indicators of metabolic syndrome in women: A randomized clinical trial. Progress in Nutrition. 2019;21:329-335.

274. Parastouei K, Nashtar SB, Al-Attar Z, Shekarchizadeh-Esfahani P, Askari G. The effects of jujube (Ziziphus jujube) on metabolic and mental health outcomes in patients with metabolic syndrome: A randomized controlled trial. Complementary Therapies in Medicine. 2024;82.

275. Park BJ, Lee YJ, Lee HR, Jung DH, Na HY, Kim HB, et al. Effects of Korean Red Ginseng on Cardiovascular Risks in Subjects with Metabolic Syndrome: a Double-blind Randomized Controlled Study. Korean J Fam Med. 2012;33(4):190-6.

276. Park SH, Jung SJ, Choi EK, Ha KC, Baek HI, Park YK, et al. The effects of steamed ginger ethanolic extract on weight and body fat loss: a randomized, double-blind, placebo-controlled clinical trial. Food Sci Biotechnol. 2020;29(2):265-273.

277. Park SH, Oh MR, Choi EK, Kim MG, Ha KC, Lee SK, et al. An 8-wk, randomized, double-blind, placebo-controlled clinical trial for the antidiabetic effects of hydrolyzed ginseng extract. Journal of Ginseng Research. 2014;38(4):239-243.

278. Parklak W, Chottidao M, Munkong N, Komindr S, Monkhai S, Wanikorn B, et al. Nutraceutical Properties of Thai Mulberry (Morus alba L.) and Their Effects on Metabolic and Cardiovascular Risk Factors in Individuals with Obesity: A Randomized, Single-Blind Crossover Trial. Nutrients. 2024;16(24).

279. Parsi A, Torkashvand M, Hajiani E, Rahimlou M, Sadeghi N. The effects of crocus sativus extract on serum lipid profile and liver enzymes in patients with non-alcoholic fatty liver disease: A randomized placebo-controlled study. Obesity Medicine. 2020;17.

280. Patel G, Dudhatra N, Chhaya G. Evaluation of the safety and efficacy of CurcuVail in patients with non-alcoholic fatty liver disease - A prospective randomized, double blind, placebo controlled, parallel group study. International Journal of Green Pharmacy. 2023;17(3):218-225.

281. Pavalakumar D, Jayasinghe M, Edirisinghe M, Wijesekara I, Senadheera S. Cinnamomum zeylanicum and Curcuma longa incorporated dairy yoghurts with hindered glycaemic properties for healthy people. Journal of Future Foods. 2021;1(1):104-112.

282. Pazzi F, Adsuar JC, Domínguez-Muñoz FJ, García-Gordillo M, Gusi N, Collado-Mateo D. Effects of Ganoderma lucidum and Ceratonia siliqua on blood glucose, lipid profile, and body composition in women with fibromyalgia. Nutr Hosp. 2021;38(1):139-145.

283. Peivandi S, Heydari-Latibari S, Ghasemzadeh F, Zamaniyan M, Bahar A, Majidi H, et al. Metabolic and endocrine changes induced by cinnamon in women with polycystic ovarian syndrome: A pilot study. Avicenna Journal of Phytomedicine. 2024;14(2):242-251.

284. Pishdad S, Nadjarzadeh A, Abargouei AS, Nazari EK, Papoli M. Effect of cumin and cinnamon on lipid profile in middle-aged women with dyslipidemia: A double blind, randomized controlled clinical trial. Progress in Nutrition. 2018;20:232-237.

285. Pol K, Ve Graaf C, Meyer D, Mars M. The efficacy of daily snack replacement with oligofructose-enriched granola bars in overweight and obese adults: A 12-week randomised controlled trial. British Journal of Nutrition. 2018;119(9):1076-1086.

286. Porasgari Z, Sakri H, Arshadi S. The effect of eight weeks of Pilates with curcumin supplementation on liver enzymes and lipid profile in overweight and obese women. Obesity Medicine. 2022;36.

287. Preza-Rodriguez L, Camacho-Luis A, Simental-Mendia LE, Martinez-Aguilar G, Gamboa-Gomez CI, Guerrero-Romero F. Turmeric Plus Black Pepper for the Improvement and Remission of Metabolic Syndrome: A Randomized, Double-Blind, Placebo-Controlled Clinical Trial. Journal of Herbal Medicine. 2024;44.

288. Puhlmann ML, Jokela R, Van Dongen KCW, Bui TPN, Hangelbroek RWJV, Smidt H, et al. Dried chicory root improves bowel function, benefits intestinal microbial trophic chains and increases faecal and circulating short chain fatty acids in subjects at risk for type 2 diabetes. Gut Microbiome. 2022;3.

289. Raatz S, Beals K, Johnson LA, Picklo M. Glycemic effect of nutritive sweeteners: Honey, sugar and high fructose corn syrup. FASEB Journal. Conference: Experimental Biology. 2015;29(1 Meeting Abstracts).

290. Rachid AP, Moncada M, de Mesquita MF, Brito J, Silva ML, Bernardo MA. Effect of Aqueous Cinnamon Extract on the Postprandial Glycemia Levels in Patients with Type 2 Diabetes Mellitus: A Randomized Controlled Trial. Nutrients. 2022;14(8).

291. Raeisi-Dehkordi H, Amiri M, Zimorovat A, Moghtaderi F, Zarei S, Forbes SC, et al. Canola oil compared with sesame and sesame-canola oil on glycaemic control and liver function in patients with type 2 diabetes: a three-way randomized triple-blind cross-over trial. Diabetes Metab Res Rev. 2021;37(5):e3399.

292. Rafie R, Hosseini SA, Hajiani E, Malehi AS, Mard SA. Effect of ginger powder supplementation in patients with non-alcoholic fatty liver disease: A randomized clinical trial. Clinical and Experimental Gastroenterology. 2020;13:35-45.

293. Rahimi G, Shams S, Aslani MR. Effects of crocin supplementation on inflammatory markers, lipid profiles, insulin and cardioprotective indices in women with PCOS: A randomized, double-blind, placebo-controlled trial. Phytotherapy Research. 2022;36(6):2605-2615.

294. Rahimi HR, Mohammadpour AH, Dastani M, Jaafari MR, Abnous K, Mobarhan MG, et al. The effect of nano-curcumin on HbA1c, fasting blood glucose, and lipid profile in diabetic subjects: A randomized clinical trial. Avicenna Journal of Phytomedicine. 2016;6(5):567-577.

295. Rahimlou M, Yari Z, Hekmatdoost A, Alavian SM, Keshavarz SA. Ginger supplementation in nonalcoholic fatty liver disease: A randomized, double-blind, placebo-controlled pilot study. Hepatitis Monthly. 2016;16(1).

296. Rajabi A, Akbar Nezhad Gharehlo A, Madadizadeh E, Basereh A, Khoramipoor K, Pirani H, et al. The effect of 12 weeks of aerobic exercise training with or without saffron supplementation on diabetes-specific markers and inflammation in women with type 2 diabetes: A randomized double-blind placebo-controlled trial. European journal of sport science. 2024;24(7):899-906.

297. Ranasinghe P, Galappatthy P, Constantine GR, Jayawardena R, Weeratunga HD, Premakumara S, et al. Efficacy and Safety of Cinnamomum zeylanicum (Ceylon cinnamon) for diabetes mellitus: a randomized, double blind, placebo-controlled clinical trial. Diabetes and Metabolic Syndrome: Clinical Research and Reviews. 2025;19(12).

298. Rasad H, Entezari MH, Ghadiri E, Mahaki B, Pahlavani N. The effect of honey consumption compared with sucrose on lipid profile in young healthy subjects (randomized clinical trial). Clinical Nutrition ESPEN. 2018;26:8-12.

299. Rathore R, Yadav A, Khatkar A, Suhag D, Vrushabaiah GK, Govindaraj S. Effect of Amla Extract on Body Mass Index, Waist Circumference, Total Body Fat, Visceral Fat, Skeletal Muscle Mass, Body Age, and Related Parameters: A Randomized, Open-Label Clinical Study in Obese Participants. Complementary Medicine Research. 2025;32(4):326-340.

300. Reay JL, Kennedy DO, Scholey AB. The glycaemic effects of single doses of Panax ginseng in young healthy volunteers. British Journal of Nutrition. 2006;96(4):639-642.

301. Reay JL, Scholey AB, Milne A, Fenwick J, Kennedy DO. Panax ginseng has no effect on indices of glucose regulation following acute or chronic ingestion in healthy volunteers. British Journal of Nutrition. 2009;101(11):1673-1678.

302. Reeds DN, Patterson BW, Okunade A, Holloszy JO, Polonsky KS, Klein S. Ginseng and ginsenoside Re do not improve β-cell function or insulin sensitivity in overweight and obese subjects with impaired glucose tolerance or diabetes. Diabetes Care. 2011;34(5):1071-6.

303. Ren Z, Gong H, Zhao A, Zhang J, Yang C, Wang P, et al. Effect of Sea Buckthorn on Plasma Glucose in Individuals with Impaired Glucose Regulation: A Two-Stage Randomized Crossover Intervention Study. Foods. 2021;10(4).

304. Rezaei M, Soltani M, Alipoor E, Rezayat SM, Vasheghani-Farahani A, Yaseri M, et al. Effect of nano-curcumin supplementation on angina status, and traditional and novel cardiovascular risk factors in overweight or obese patients with coronary slow flow phenomenon: a randomized double-blind placebo-controlled clinical trial. BMC Nutrition. 2024;10:73.

Additional report: Soltani M, Hosseinzadeh-Attar MJ, Rezaei M, Alipoor E, Vasheghani-Farahani A, Yaseri M, et al. Effect of nano-curcumin supplementation on cardiometabolic risk factors, physical and psychological quality of life, and depression in patients with coronary slow flow phenomenon: a randomized double-blind clinical trial. Trials. 2024;25:515.

305. Rezaiee R, Ghazizadeh H, Darroudi S, Zirak RG, Rohban M, Eidi M, et al. The impacts of Crocin on serum cytokines concentrations in participants with metabolic syndrome: a double-blind randomized clinical trial study. Scientific reports. 2025;15(1):29975.

306. Rhee MY, Kim YS, Bae JH, Nah DY, Kim YK, Lee MM, et al. Effect of Korean red ginseng on arterial stiffness in subjects with hypertension. Journal of Alternative and Complementary Medicine. 2011;17(1):45-49.

307. Riche DM, Riche KD, East HE, Barrett EK, May WL. Impact of mulberry leaf extract on type 2 diabetes (Mul-DM): A randomized, placebo-controlled pilot study. Complementary Therapies in Medicine. 2017;32:105-108.

308. Romeo GR, Lee J, Mulla CM, Noh Y, Holden C, Lee BC. Influence of cinnamon on glycemic control in individuals with prediabetes: A randomized controlled trial. Journal of the Endocrine Society. 2020;4(11).

309. Rostamizadeh P, Asl SMKH, Far ZG, Ahmadijoo P, Mahmudiono T, Bokov DO, et al. Effects of licorice root supplementation on liver enzymes, hepatic steatosis, metabolic and oxidative stress parameters in women with nonalcoholic fatty liver disease: A randomized double-blind clinical trial. Phytotherapy Research. 2022;36(10):3949-3956.

310. Rostamkhani H, Veisi P, Niknafs B, Jafarabadi MA, Ghoreishi Z. The effect of zingiber officinale on prooxidant-antioxidant balance and glycemic control in diabetic patients with ESRD undergoing hemodialysis: a double-blind randomized control trial. BMC Complementary Medicine and Therapies. 2023;23(1).

311. Roussel AM, Hininger I, Benaraba R, Ziegenfuss TN, Anderson RA. Antioxidant effects of a cinnamon extract in people with impaired fasting glucose that are overweight or obese. Journal of the American College of Nutrition. 2009;28(1):16-21.

312. Saadati S, Hatami B, Yari Z, Shahrbaf MA, Eghtesad S, Mansour A, et al. The effects of curcumin supplementation on liver enzymes, lipid profile, glucose homeostasis, and hepatic steatosis and fibrosis in patients with non-alcoholic fatty liver disease. European Journal of Clinical Nutrition. 2019;73(3):441-449.

313. Sabzghabaee AM, Kelishadi R, Jelokhanian H, Asgary S, Ghannadi A, Badri S. Clinical effects of Portulaca oleracea seeds on dyslipidemia in obese adolescents: a triple-blinded randomized controlled trial. Medicinski arhiv. 2014;68(3):195-199.

314. Sabzghabaee AM, Khayam I, Kelishadi R, Ghannadi A, Soltani R, Badri S, et al. Effect of Zizyphus jujuba fruits on dyslipidemia in obese adolescents: a triple-masked randomized controlled clinical trial. Medical archives. 2013;67(3):156-159.

315. Sadeghi F, Akhlaghi M, Salehi S. Adverse effects of honey on low-density lipoprotein cholesterol and adiponectin concentrations in patients with type 2 diabetes: a randomized controlled cross-over trial. Journal of Diabetes and Metabolic Disorders. 2020;19(1):373-380.

316. Sadeghzadeh Z, Ostadrahimi A, Ranjbar M, Farshbaf-Khalili A. The Efficacy of Nigella sativa L. and Curcumin Nanomicelle Alone or Together on Lipid Profile, Glycemic Control Indices, and Serum 17-Β Estradiol in Postmenopausal Women. J Caring Sci. 2023;12(3):163-173.

317. Safari Z, Bagherniya M, Khoram Z, Ebrahimi Varzaneh A, Heidari Z, Sahebkar A, et al. The effect of curcumin on anthropometric indices, blood pressure, lipid profiles, fasting blood glucose, liver enzymes, fibrosis, and steatosis in non-alcoholic fatty livers. Front Nutr. 2023;10:1163950.

318. Saghafi N, Ghazanfarpour M, Khadivzadeh T, Babakhanian M, Afiat M. The Effect of Foeniculum Vulgare (Fennel) on Body Composition in Postmenopausal Women with Excess Weight: A Double-blind Randomized Placebo-controlled Trial. J Menopausal Med. 2017;23(3):166-171.

319. Sahib AS. Anti-diabetic and antioxidant effect of cinnamon in poorly controlled type-2 diabetic Iraqi patients: A randomized, placebo-controlled clinical trial. J Intercult Ethnopharmacol. 2016;5(2):108-13.

320. Salazar MH, Flores A, Ramírez E, Díaz JL, Rodríguez B, Castro H. Effect of avocado honey on anthropometric and biochemical parameters in healthy subjects: a pilot randomised controlled trial. CYTA-JOURNAL OF FOOD. 2022;20(1):78-85.

321. Samsamikor M, Mackay DS, Mollard RC, Alashi AM, Aluko RE. Hemp seed protein and its hydrolysate compared with casein protein consumption in adults with hypertension: a double-blind crossover study. American Journal of Clinical Nutrition. 2024;120(1):56-65.

322. Sangouni AA, Taghdir M, Mirahmadi J, Sepandi M, Parastouei K. Effects of curcumin and/or coenzyme Q10 supplementation on metabolic control in subjects with metabolic syndrome: a randomized clinical trial. Nutrition Journal. 2022;21(1).

323. Saraf-Bank S, Ahmadi A, Paknahad Z, Maracy M, Nourian M. Effects of curcumin supplementation on markers of inflammation and oxidative stress among healthy overweight and obese girl adolescents: A randomized placebo-controlled clinical trial. Phytotherapy Research. 2019;33(8):2015-2022.

324. Sengsuk C, Sanguanwong S, Tangvarasittichai O, Tangvarasittichai S. Effect of cinnamon supplementation on glucose, lipids levels, glomerular filtration rate, and blood pressure of subjects with type 2 diabetes mellitus. Diabetology International. 2016;7(2):124-132.

325. Seo SK, Hong Y, Yun BH, Chon SJ, Jung YS, Park JH, et al. Antioxidative effects of Korean red ginseng in postmenopausal women: A double-blind randomized controlled trial. Journal of Ethnopharmacology. 2014;154(3):753-757.

326. Seong E, Bose S, Han SY, Song EJ, Lee M, Nam YD, et al. Positive influence of gut microbiota on the effects of Korean red ginseng in metabolic syndrome: a randomized, double-blind, placebo-controlled clinical trial. EPMA Journal. 2021;12(2):177-197.

327. Sepahi S, Golfakhrabadi M, Bonakdaran S, Lotfi H, Mohajeri SA. Effect of crocin on diabetic patients: A placebo-controlled, triple-blinded clinical trial. Clinical Nutrition ESPEN. 2022;50:255-263.

328. Shafabakhsh R, Asemi Z, Reiner Z, Soleimani A, Aghadavod E, Bahmani F. The effects of nano-curcumin on metabolic status in patients with diabetes on hemodialysis, a randomized, double blind, placebo-controlled trial. Iranian Journal of Kidney Diseases. 2020;14(4):290-299.

329. Shafabakhsh R, Mobini M, Raygan F, Aghadavod E, Ostadmohammadi V, Amirani E, et al. Curcumin administration and the effects on psychological status and markers of inflammation and oxidative damage in patients with type 2 diabetes and coronary heart disease. Clinical Nutrition ESPEN. 2020;40:77-82.

330. Shahri SHH, Soltani G, Teshnizi MA, Moeinipour A, Tayyebi M, Bayaz RJD, et al. Evaluation of the effect of crocin on prevention of atrial fibrillation after coronary artery bypass grafting or heart valve replacement: A double-blind, randomized, placebo-controlled trial. Avicenna Journal of Phytomedicine. 2022;12(6):589-601.

331. Sharifi S, Bagherniya M, Khoram Z, Ebrahimi Varzaneh A, Atkin SL, Jamialahmadi T, et al. Efficacy of curcumin plus piperine co-supplementation in moderate-to-high hepatic steatosis: A double-blind, randomized, placebo-controlled clinical trial. Phytotherapy Research. 2023;37(6):2217-2229.

332. Sharma PK, Siva Rami Reddy E. Efficacy of glycyrrhiza glabra mother tincture in hyperlipidemia patients: A double blind randomized controlled trial. Journal of Global Trends in Pharmaceutical Sciences. 2021;12(1):9019-9025.

333. Shidfar F, Rajab A, Rahideh T, Khandouzi N, Hosseini S, Shidfar S. The effect of ginger (Zingiber officinale) on glycemic markers in patients with type 2 diabetes. Journal of Complementary and Integrative Medicine. 2015;12(2):165-170.

334. Shirzad F, Morovatdar N, Rezaee R, Tsarouhas K, Moghadam AA. Cinnamon effects on blood pressure and metabolic profile: A double-blind, randomized, placebo-controlled trial in patients with stage 1 hypertension. Avicenna Journal of Phytomedicine. 2021;11(1):91-100.

335. Shishtar E, Jovanovski E, Jenkins A, Vuksan V. Effects of Korean White Ginseng (Panax Ginseng C.A. Meyer) on Vascular and Glycemic Health in Type 2 Diabetes: Results of a Randomized, Double Blind, Placebo-controlled, Multiple-crossover, Acute Dose Escalation Trial. Clin Nutr Res. 2014;3(2):89-97.

336. Sievenpiper JL, Arnason JT, Leiter LA, Vuksan V. Decreasing, null and increasing effects of eight popular types of ginseng on acute postprandial glycemic indices in healthy humans: The role of ginsenosides. Journal of the American College of Nutrition. 2004;23(3):248-258.

337. Sievenpiper JL, Arnason JT, Leiter LA, Vuksan V. Null and Opposing Effects of Asian Ginseng (Panax ginseng C.A. Meyer) on Acute Glycemia: Results of Two Acute Dose Escalation Studies. Journal of the American College of Nutrition. 2003;22(6):524-532.

338. Sievenpiper JL, Arnason JT, Leiter LA, Vuksan V. Variable effects of American ginseng: A batch of American ginseng (Panax quinquefolius L.) with a depressed ginsenoside profile does not affect postprandial glycemia. European Journal of Clinical Nutrition. 2003;57(2):243-248.

339. Sievenpiper JL, Sung MK, Di Buono M, Seung-Lee K, Ki YN, Arnason JT, et al. Korean red ginseng rootlets decrease acute postprandial glycemia: Results from sequential preparation- and dose-finding studies. Journal of the American College of Nutrition. 2006;25(2):100-107.

340. Silalahi T, Alwi I, Suyatna F, Sartika KD. Curcumin's Effect on Inflammatory Response following Percutaneous Coronary Intervention in Adult Patients with Stable Coronary Heart Disease. International Journal of Angiology. 2021;30(2):132-138.

341. Silva TAL, Medeiros DC, Medeiros GCBS, Medeiros RCSC, De Souza Araujo J, Medeiros JA, et al. Influence of curcumin supplementation on metabolic and lipid parameters of people living with HIV/AIDS: A randomized controlled trial. BMC Complementary and Alternative Medicine. 2019;19(1).

342. Sinclair J, Sant B, Du X, Shadwell G, Dillon S, Butters B, et al. Effects of peppermint (Mentha x piperita L.) oil on cardiometabolic outcomes in patients with pre- and stage 1 hypertension: A placebo randomized controlled trial. PLoS ONE. 2026;21(4).

343. Soares APD, de Faria NC, Graciano GF, da Silva JA, Goncalves VSD, Valenzuela VDT, et al. Ginger infusion increases diet-induced thermogenesis in healthy individuals: A randomized crossover trial. FOOD BIOSCIENCE. 2022;50.

344. Soares APD, de Faria NC, Graciano GF, dos Santos ALS, Valenzuela VD, Correia Mitd, et al. Cinnamon Infusion Reduces Satiety and Increases Energy Intake: A Randomized Crossover Trial. Annals of Nutrition and Metabolism. 2022;78(5):265-272.

345. Sohaei S, Amani R, Tarrahi MJ, Ghasemi-Tehrani H. The effects of curcumin supplementation on glycemic status, lipid profile and hs-CRP levels in overweight/obese women with polycystic ovary syndrome: A randomized, double-blind, placebo-controlled clinical trial. Complementary Therapies in Medicine. 2019;47.

346. Son WH, Park HT, Jeon BH, Ha MS. Effects of fermented oyster extract supplementation on free fatty acid and liver enzymes in older women with obesity. Phys Act Nutr. 2024;28(3):58-63.

347. Sotaniemi EA, Haapakoski E, Rautio A. Ginseng therapy in non-insulin-dependent diabetic patients. Effects of psychophysical performance, glucose homeostasis, serum lipids, serum aminoterminalpropeptide concentration, and body weight. Diabetes Care. 1995;18(10):1373-1375.

348. Srinivasan A, Selvarajan S, Kamalanathan S, Kadhiravan T, Prasanna Lakshmi NC, Adithan S. Effect of Curcuma longa on vascular function in native Tamilians with type 2 diabetes mellitus: A randomized, double-blind, parallel arm, placebo-controlled trial. Phytotherapy Research. 2019;33(7):1898-1911.

349. Stavro PM, Woo M, Heim TF, Leiter LA, Vuksan V. North American ginseng exerts a neutral effect on blood pressure in individuals with hypertension. Hypertension. 2005;46(2):406-411.

350. Stavro PM, Woo M, Leiter LA, Heim TF, Sievenpiper JL, Vuksan V. Long-term intake of North American ginseng has no effect on 24-hour blood pressure and renal function. Hypertension. 2006;47(4):791-796.

351. Tabatabaei-Malazy O, Lavari N, Abdollahi M. Natural Products in the Clinical Management of Metabolic Syndrome. Handbook of Experimental Pharmacology. 2025;287:123-157.

352. Tafti MA, Moein M, Babajafari S, Haem E, Zarshenas MM. Efficacy of Herbal Supplementation of Purslane Freeze-dried Juice for Weight Loss: An 8-week Randomized, Double-blind, Placebo-controlled Clinical Trial. Current Traditional Medicine. 2024;10(1):81-90.

353. Taghizadeh M, Mohammad Zadeh A, Asemi Z, Farrokhnezhad AH, Memarzadeh MR, Banikazemi Z, et al. Morus Alba leaf extract affects metabolic profiles, biomarkers inflammation and oxidative stress in patients with type 2 diabetes mellitus: A double-blind clinical trial. Clinical Nutrition ESPEN. 2022;49:68-73.

354. Takahashi M, Mineshita Y, Yamagami J, Wang C, Fujihira K, Tahara Y, et al. Effects of the timing of acute mulberry leaf extract intake on postprandial glucose metabolism in healthy adults: a randomised, placebo-controlled, double-blind study. European Journal of Clinical Nutrition. 2023;77(4):468-473.

355. Takuathung MN, Settakorn K, Aisara J, Dukaew N, Inpan R, Chitphan J, et al. Effects of the Longan pulp and seed extract beverage product on bone turnover markers, lipid profiles, glycemic markers, and safety profiles in the elderly: A randomized, double-blind, placebo-controlled trial. JOURNAL OF FUNCTIONAL FOODS. 2025;129.

356. Tamaddoni A, Nasseri E, Mohammadi E, Qujeq D, Zayeri F, Zand H, et al. A Double-blind Randomized Controlled Trial of Curcumin for Improvement in Glycemic Status, Lipid Profile and Systemic Inflammation in beta-Thalassemia Major. Journal of Herbal Medicine. 2020;21.

357. Tang M, Larson-Meyer DE, Liebman M. Effect of cinnamon and turmeric on urinary oxalate excretion, plasma lipids, and plasma glucose in healthy subjects. American Journal of Clinical Nutrition. 2008;87(5):1262-1267.

358. Tehrani SD, Hosseini A, Shahzamani M, Heidari Z, Askari G, Majeed M, et al. Evaluation of the effectiveness of curcumin and piperine co-supplementation on inflammatory factors, cardiac biomarkers, atrial fibrillation, and clinical outcomes after coronary artery bypass graft surgery. Clinical Nutrition ESPEN. 2024;62:57-65.

359. Thaipitakwong T, Supasyndh O, Rasmi Y, Aramwit P. A randomized controlled study of dose-finding, efficacy, and safety of mulberry leaves on glycemic profiles in obese persons with borderline diabetes. Complementary Therapies in Medicine. 2020;49.

360. Thomas J, Patel A, Das Sivadasan S, Sreevallabhan S, Madhavamenon KI, Mohanan R. Clove bud (Syzygium aromaticum L.) polyphenol helps to mitigate metabolic syndrome by establishing intracellular redox homeostasis and glucose metabolism: A randomized, double-blinded, active-controlled comparative study. JOURNAL OF FUNCTIONAL FOODS. 2022;98.

361. Thondre PS, Butler I, Tammam J, Achebe I, Young E, Lane M, Gallagher A. Understanding the impact of different doses of Reducose mulberry leaf extract on blood glucose and insulin responses after eating a complex meal: results from a double-blind, randomised, crossover trial. Nutrients. 2024;16(11):1670.

362. Thondre PS, Lightowler H, Ahlstrom L, Gallagher A. Mulberry leaf extract improves glycaemic response and insulaemic response to sucrose in healthy subjects: results of a randomized, double blind, placebo-controlled study. Nutrition and Metabolism. 2021;18(1).

363. Thota RN, Acharya SH, Garg ML. Curcumin and/or omega-3 polyunsaturated fatty acids supplementation reduces insulin resistance and blood lipids in individuals with high risk of type 2 diabetes: A randomised controlled trial. Lipids in Health and Disease. 2019;18(1).

364. Thota RN, Dias CB, Abbott KA, Acharya SH, Garg ML. Curcumin alleviates postprandial glycaemic response in healthy subjects: A cross-over, randomized controlled study. Scientific reports. 2018;8(1):13679.

365. Toh DWK, Zhou H, Cazenave-Gassiot A, Choi H, Burla B, Bendt AK, et al. Effects of wolfberry (Lycium barbarum) consumption on the human plasma lipidome and its association with cardiovascular disease risk factors: a randomized controlled trial of middle-aged and older adults. Front Nutr. 2024;11:1258570.

366. Tominaga Y, Mae T, Kitano M, Sakamoto Y, Ikematsu H, Nakagawa K. Licorice flavonoid oil effects body weight loss by reduction of body fat mass in overweight subjects. Journal of Health Science. 2006;52(6):672-683.

367. Tominaga Y, Nakagawa K, Mae T, Kitano M, Yokota S, Arai T, et al. Licorice flavonoid oil reduces total body fat and visceral fat in overweight subjects: A randomized, double-blind, placebo-controlled study. Obesity Research and Clinical Practice. 2009;3(3):169-178.

368. Uchio R, Muroyama K, Okuda-Hanafusa C, Kawasaki K, Yamamoto Y, Murosaki S. Hot water extract of Curcuma longa L. Improves serum inflammatory markers and general health in subjects with overweight or prehypertension/mild hypertension: A randomized, double-blind, placebo-controlled trial. Nutrients. 2019;11(8).

369. Uchio R, Okuda-Hanafusa C, Sakaguchi H, Saji R, Muroyama K, Murosaki S, et al. Curcuma longa extract reduces serum inflammatory markers and postprandial hyperglycemia in healthy but borderline participants with overweight and glycemia in the normal/prediabetes range: a randomized, double-blind, and placebo-controlled trial. Front Nutr. 2024;11:1324196.

370. Ulyanova O, Maleev Y. Features of Nutrition of Patients with Cardiovascular Diseases. E Journal of Cardiovascular Medicine. Conference: 17th International Congress of Update in Cardiology and Cardiovascular Surgery. Virtual.. 2021;9(1).

371. Usharani P, Fatima N, Muralidhar N. Effects of Phyllanthus emblica extract on endothelial dysfunction and biomarkers of oxidative stress in patients with type 2 diabetes mellitus: A randomized, double-blind, controlled study. Diabetes, Metabolic Syndrome and Obesity. 2013;6:275-284.

372. Usharani P, Merugu PL, Nutalapati C. Evaluation of the effects of a standardized aqueous extract of Phyllanthus emblica fruits on endothelial dysfunction, oxidative stress, systemic inflammation and lipid profile in subjects with metabolic syndrome: A randomised, double blind, placebo controlled clinical study. BMC Complementary and Alternative Medicine. 2019;19(1).

373. Usharani P, SudhaRani E, KiranKishore K, Raveendranath P. Evaluation of the effect of a standardized aqueous extract of the fruits of Emblica officinalis on mental stress induced cardiovascular changes in healthy human subjects. International Journal of Pharmaceutical Sciences and Research. 2017;8(10):4138-4146.

374. Vafa M, Mohammadi F, Shidfar F, Sormaghi MS, Heidari I, Golestan B, et al. Effects of cinnamon consumption on glycemic status, lipid profile and body composition in type 2 diabetic patients. International Journal of Preventive Medicine. 2012;3(8):531-536.

375. van den Driessche JJ, Plat J, Plasqui G, Mensink RP. A Single Dose of Goji Berries Does Not Affect Postprandial Energy Expenditure and Substrate Oxidation in Healthy, Overweight Men. J Nutr Metab. 2019;2019:4057143.

376. Veisi P, Rostamkhani H, Niknafs B, Asghari Jafarabadi M, Ghoreishi Z. Effect of Zingiber officinale on Lipid Profile and Some Inflammatory Markers in Diabetic Hemodialysis Patients: A Randomized Double-Blind Placebo-Controlled Clinical Trial. Evidence-based Complementary and Alternative Medicine. 2023;2023.

377. Vuksan V, Sievenpiper JL, Koo VYY, Francis T, Beljan-Zdravkovic U, Xu Z, et al. American ginseng (Panax quinquefolius L) reduces postprandial glycemia in nondiabetic subjects and subjects with type 2 diabetes mellitus. Archives of Internal Medicine. 2000;160(7):1009-1013.

378. Vuksan V, Stavro MP, Sievenpiper JL, Beljan-Zdravkovic U, Leiter LA, Josse RG, et al. Similar postprandial glycemic reductions with escalation of dose and administration time of American ginseng in type 2 diabetes. Diabetes Care. 2000;23(9):1221-1226.

379. Vuksan V, Sung MK, Sievenpiper JL, Stavro PM, Jenkins AL, Di Buono M, et al. Korean red ginseng (Panax ginseng) improves glucose and insulin regulation in well-controlled, type 2 diabetes: Results of a randomized, double-blind, placebo-controlled study of efficacy and safety. Nutrition, Metabolism and Cardiovascular Diseases. 2008;18(1):46-56.

380. Vuksan V, Xu ZZ, Jovanovski E, Jenkins AL, Beljan-Zdravkovic U, Sievenpiper JL, et al. Efficacy and safety of American ginseng (Panax quinquefolius L.) extract on glycemic control and cardiovascular risk factors in individuals with type 2 diabetes: a double-blind, randomized, cross-over clinical trial. European Journal of Nutrition. 2019;58(3):1237-1245.

381. Wachtel-Galor S, Tomlinson B, Benzie IFF. Ganoderma lucidum ('Lingzhi'), a Chinese medicinal mushroom: Biomarker responses in a controlled human supplementation study. British Journal of Nutrition. 2004;91(2):263-269.

382. Wainstein J, Landau Z, Dayan YB, Jakubowicz D, Grothe T, Perrinjaquet-Moccetti T, et al. Purslane extract and glucose homeostasis in adults with type 2 diabetes: A double-blind, placebo-controlled clinical trial of efficacy and safety. Journal of Medicinal Food. 2016;19(2):133-140.

383. Walker AF, Marakis G, Morris AP, Robinson PA. Promising hypotensive effect of hawthorn extract: A randomized double-blind pilot study of mild, essential hypertension. Phytotherapy Research. 2002;16(1):48-54.

384. Walker AF, Marakis G, Simpson E, Hope JL, Robinson PA, Hassanein M, et al. Hypotensive effects of hawthorn for patients with diabetes taking prescription drugs: A randomised controlled trial. British Journal of General Practice. 2006;56(527):437-443.

385. Wang JG, Anderson RA, Graham IiiGM, Chu MC, Sauer MV, Guarnaccia MM, et al. The effect of cinnamon extract on insulin resistance parameters in polycystic ovary syndrome: a pilot study. Fertility and Sterility. 2007;88(1):240-243.

386. Wang R, Li Y, Mu W, Li Z, Sun J, Wang B, et al. Mulberry leaf extract reduces the glycemic indexes of four common dietary carbohydrates. Medicine. 2018;97(34).

387. Wang X, Zhao L, Zhou F. Clinical Evaluation of Ganoderma lucidum Spore Oil for Triglyceride Reduction: A Randomized, Double-Blind, Crossover Study. Nutrients. 2025;17(5).

388. Wang Y, Yu Z, Jiang J, Li Y, Yu S. Mulberry leaf attenuates atherosclerotic lesions in patients with coronary heart disease possibly via 1-Deoxynojirimycin: A placebo-controlled, double-blind clinical trial. Journal of food biochemistry. 2021;45(1):e13573.

389. Wei J, Liu A, Fan Z, Peng X, Lou X, Lu X, et al. Cooking Increased the Postprandial Glycaemic Response but Enhanced the Preload Effect of Air-Dried Jujube. Foods. 2025;14(7).

390. Wickenberg J, Ingemansson SL, Hlebowicz J. Effects of Curcuma longa (turmeric) on postprandial plasma glucose and insulin in healthy subjects. Nutrition Journal. 2010;9(1).

391. Wickenberg J, Lindstedt S, Berntorp K, Nilsson J, Hlebowicz J. Ceylon cinnamon does not affect postprandial plasma glucose or insulin in subjects with impaired glucose tolerance. British Journal of Nutrition. 2012;107(12):1845-1849.

392. Wickenberg J, Lindstedt S, Nilsson J, Hlebowicz J. Cassia cinnamon does not change the insulin sensitivity or the liver enzymes in subjects with impaired glucose tolerance. Nutrition Journal. 2014;13(1).

393. Wongcharoen W, et al. Effects of curcuminoids on frequency of acute myocardial infarction after coronary artery bypass grafting. Am J Cardiol. 2012;110(1):40-44.

394. Woottisin S, Imphat C, Munjit S, Woottisin N. Efficacy and Safety of Black Pepper Supplementation in Adults with Borderline Hypercholesterolemia: A Randomized, Placebo-Controlled Trial. Tropical Journal of Natural Product Research. 2026;10(1):6825-6834.

395. Wu JHY, Hodgson JM, Puddey IB, Belski R, Burke V, Croft KD. Sesame supplementation does not improve cardiovascular disease risk markers in overweight men and women. Nutr Metab Cardiovasc Dis. 2009;19(11):774-780.

396. Yaikwawong M, Kamdee K, Chuengsamarn S. Curcumin Attenuates Liver Steatosis via Antioxidant and Anti-Inflammatory Pathways in Obese Patients with Type 2 Diabetes Mellitus: A Randomized Controlled Trial. International Journal of Molecular Sciences. 2025;26(19):9286. doi:10.3390/ijms26199286.

Additional report: Yaikwawong M, Jansarikit L, Jirawatnotai S, Chuengsamarn S. The Effect of Curcumin on Reducing Atherogenic Risks in Obese Patients with Type 2 Diabetes: A Randomized Controlled Trial. Nutrients. 2024;16(15):2441. doi:10.3390/nu16152441.

Additional report: Yaikwawong M, Jansarikit L, Jirawatnotai S, Chuengsamarn S. Curcumin extract improves beta cell functions in obese patients with type 2 diabetes: a randomized controlled trial. Nutrition Journal. 2024;23:119.

397. Yang YS, Su YF, Yang HW, Lee YH, Chou JI, Ueng KC. Lipid-lowering effects of curcumin in patients with metabolic syndrome: A randomized, double-blind, placebo-controlled trial. Phytotherapy Research. 2014;28(12):1770-1777.

398. Yazdanpanah Z, Ghadiri-Anari A, Mehrjardi AV, Dehghani A, Zardini HZ, Nadjarzadeh A. Effect of Ziziphus jujube Fruit Infusion on Lipid Profiles, Glycaemic Index and Antioxidant Status in Type 2 Diabetic Patients: A Randomized Controlled Clinical Trial. Phytotherapy Research. 2017;31(5):755-762.

399. Ye L, Wang X, Konno T, Gong X, Ding H, Yan H, et al. Fat reducing effects of Nelumbo nucifera leaf extract in overweight patients. Natural Product Research. 2022;36(18):4776-4781.

400. Yekefallah L, Aghakhanbeigi F, Jalalpour A, Namdar P, Mafi MH. Comparing the Effects of Ganoderma lucidum and Kombucha Mushrooms on Glycemic Control in Patients With Type 2 Diabetes: A Randomized Clinical Trial. Clinical Diabetes. 2025;43(5):813-822.

401. Yoon JW, Kang SM, Vassy JL, Shin H, Lee YH, Ahn HY, et al. Efficacy and safety of ginsam, a vinegar extract from Panax ginseng, in type 2 diabetic patients: Results of a double-blind, placebo-controlled study. Journal of Diabetes Investigation. 2012;3(3):309-317.

402. Yousefi-Nodeh H, Farshbaf-Khalili A, Sadeghzadeh Oskouei B, Jafarilar-Aghdam N, Kazemi-Zanjani N, Pourzeinali S. Curcumin and vitamin E improve hot flashes, lipid profile, and fasting blood glucose without any detrimental effect on the liver and renal function in postmenopausal women: A triple-blind placebo-controlled clinical trial. Health care for women international. 2024;45(11):1184-1206.

403. Zahedifar A, Khodashenas M, Bijari B, Zahedifar F. Effects of cinnamon on fasting blood sugar and hemoglobin A1C in patients with type II diabetes mellitus: A randomized clinical trial. [Persian]. Journal of Mazandaran University of Medical Sciences. 2018;27(156):80-88.

404. Zakerkish M, Hemmati A, Sebardan FJ, Maram NS. Effect of Curcumex on Serum Lipid Profile and Fasting Blood Glucose, HbA1c, and Insulin Resistance Levels in Type 2 Diabetic Patients: A Randomized, Double-Blind Clinical Trial. Jundishapur Journal of Natural Pharmaceutical Products. 2023;18(3).

405. Zamany S, Barzegar A, Aghapour B, Ferdosi F. Effects of Dietary Supplementation with Coriander Seed Powder on Serum Lipid Profile, Glycemic Indices, and Oxidative Stress Biomarkers in Patients with Type 2 Diabetes Mellitus: A Randomized, Double-Blind, Placebo-Controlled Trial. Prev Nutr Food Sci. 2025;30(6):529-538.

406. Zanzer YC, Plaza M, Dougkas A, Turner C, Ostman E. Black pepper-based beverage induced appetite-suppressing effects without altering postprandial glycaemia, gut and thyroid hormones or gastrointestinal well-being: a randomized crossover study in healthy subjects. Food & function. 2018;9(5):2774-2786.

407. Zare R, Nadjarzadeh A, Zarshenas MM, Shams M, Heydari M. Efficacy of cinnamon in patients with type II diabetes mellitus: A randomized controlled clinical trial. Clinical Nutrition. 2019;38(2):549-556.

408. Zarezadeh M, Saedisomeolia A, Khorshidi M, Kord Varkane H, Makhdoomi Arzati M, Abdollahi M, et al. Asymmetric dimethylarginine and soluble inter-cellular adhesion molecule-1 serum levels alteration following ginger supplementation in patients with type 2 diabetes: A randomized double-blind, placebo-controlled clinical trial. Journal of Complementary and Integrative Medicine. 2019;16(2).

409. Zelicha H, Yang J, Henning SM, Huang J, Lee RP, Thames G, et al. Effect of cinnamon spice on continuously monitored glycemic response in adults with prediabetes: a 4-week randomized controlled crossover trial. American Journal of Clinical Nutrition. 2024;119(3):649-657.

Additional report: Zelicha H, Yang J, Henning SM, Huang J, Lee RP, Thames G, et al. Effect of Cinnamon Spice on Continuous Glucose Monitoring Response in Obese Individuals with Pre-Diabetes; A Randomized Controlled Crossover Trial. Current Developments in Nutrition. 2023;7(Supplement 1).

410. Zeng W, Chu TTW, Fok BSP, Ho WKK, Chan JCN, Tomlinson B. Effects of Hawthorn Fruit Extract Drink in Chinese Patients With Mild Hypertension and/or Hyperlipidaemia: A Randomized, Double-Blind, Placebo-Controlled, Crossover Study. Dose Response. 2024;22(4).

411. Zhao W, et al. Yam paste in glycemic preloads curbs peak glycemia of rice meals in apparent healthy subjects. Asia Pac J Clin Nutr. 2021;30(3):436-445.

412. Zhou F, Zhang J, Zhao A, Zhang Y, Wang P. Effects of sea buckthorn puree on risk factors of cardiovascular disease in hypercholesterolemia population: a double-blind, randomized, placebo-controlled trial. Anim Biotechnol. 2022;33(5):955-963.

413. Zhou X, Yu JW, Wan Q, Wang W, Yu XY, You JY, et al. Efficacy and safety of Pueraria lobata radix and Pueraria thomsonii radix for patients with mild dyslipidemia: A randomized, double-blind, placebo-controlled trial. JOURNAL OF FUNCTIONAL FOODS. 2022;98.

414. Zick SM, Gillespie B, Aaronson KD. The effect of Crataegus oxycantha Special Extract WS 1442 on clinical progression in patients with mild to moderate symptoms of heart failure. Eur J Heart Fail. 2008;10(6):587-93.

415. Ziegenfuss TN, Hofheins JE, Mendel RW, Landis J, Anderson RA. Effects of a water-soluble cinnamon extract on body composition and features of the metabolic syndrome in pre-diabetic men and women. J Int Soc Sports Nutr. 2006;3(2):45-53.

416. Zilaee M, Soukhtanloo M, Ghayour-Mobarhan M, Shemshian M, Salehi M, Ferns GAA. Effect of saffron on serum leptin levels in patients with metabolic syndrome, a double-blind, randomized and placebo-controlled trial study. Progress in Nutrition. 2018;20(Supplement 1):140-144.

**S4. Representative full-text exclusions and reasons**

This section lists 73 representative examples of the 677 reports excluded at full-text assessment. The complete exclusion counts by reason are shown in Figure 1.

1. Akhondzadeh S, Mostafavi SA, Keshavarz SA, Mohammadi MR, Hosseini S, Eshraghian MR. A placebo controlled randomized clinical trial of Crocus sativus L. (saffron) on depression and food craving among overweight women with mild to moderate depression. Journal of Clinical Pharmacy and Therapeutics. 2020;45(1):134-143. Excluded: Primary outcome not cardiometabolic.

2. Alvarenga L, Cardozo LFMF, Da Cruz BO, Paiva BR, Fouque D, Mafra D. Curcumin supplementation improves oxidative stress and inflammation biomarkers in patients undergoing hemodialysis: a secondary analysis of a randomized controlled trial. International Urology and Nephrology. 2022;54(10):2645-2652. Excluded: Primary outcome not cardiometabolic.

3. Amagase H, Nance DM. A randomized, double-blind, placebo-controlled, clinical study of the general effects of a standardized Lycium barbarum (Goji) Juice, GoChi. J Altern Complement Med. 2008;14(4):403-12. Excluded: Primary outcome not cardiometabolic.

4. Asadi S, Gholami MS, Siassi F, Qorbani M, Khamoshian K, Sotoudeh G. Nano curcumin supplementation reduced the severity of diabetic sensorimotor polyneuropathy in patients with type 2 diabetes mellitus: A randomized double-blind placebo- controlled clinical trial. Complementary Therapies in Medicine. 2019;43:253-260. Excluded: Primary outcome not cardiometabolic.

5. Askari F, Ghaffari F, Alaee N, Naseri M, Tayanloo-Beik A. Effect of Citrus aurantium L. on pain and blood pressure in patients undergoing hand reconstructive surgery. Pain Manag. 2025;15(8):491-500. Excluded: Primary outcome not cardiometabolic.

6. Biswas TK, Chakrabarti S, Pandit S, Jana U, Dey SK. Pilot study evaluating the use of Emblica officinalis standardized fruit extract in cardio-respiratory improvement and antioxidant status of volunteers with smoking history. . 2014;4(4):188-194. Excluded: Multi-ingredient product with a non-catalogue active ingredient.

7. Caron MF, Hotsko AL, Robertson S, Mandybur L, Kluger J, White CM. Electrocardiographic and hemodynamic effects of Panax ginseng. Annals of Pharmacotherapy. 2002;36(5):758-763. Excluded: Primary outcome not cardiometabolic.

8. Chen CL, Tseng JH, Pan TM, Hsiao SH. A Randomized, Double-Blind Clinical Study on Blood Pressure Reduction and Blood Lipid Profile Amelioration on Treatment with Ankascin 568. CHINESE JOURNAL OF PHYSIOLOGY. 2017;60(3):158-165. Excluded: Species or edible plant part not in the catalogue.

9. Cheraghpour M, Imani H, Ommi S, Alavian SM, Karimi-Shahrbabak E, Hedayati M, et al. Hesperidin improves hepatic steatosis, hepatic enzymes, and metabolic and inflammatory parameters in patients with nonalcoholic fatty liver disease: A randomized, placebo-controlled, double-blind clinical trial. Phytotherapy Research. 2019;33(8):2118-2125. Excluded: Substance not on the medicine-food homology catalogue.

10. Constans J, Bennetau-Pelissero C, Martin JF, Rock E, Mazur A, Bedel A, et al. Marked antioxidant effect of orange juice intake and its phytomicronutrients in a preliminary randomized cross-over trial on mild hypercholesterolemic men. Clinical Nutrition. 2015;34(6):1093-1100. Excluded: Species or edible plant part not in the catalogue.

11. de Jaeger C, Kruiskamp S, Voronska E, Lamberti C, Baramki H, Beaudeux JL, et al. A Natural Astragalus-Based Nutritional Supplement Lengthens Telomeres in a Middle-Aged Population: A Randomized, Double-Blind, Placebo-Controlled Study. Nutrients. 2024;16(17). Excluded: Primary outcome not cardiometabolic.

12. Del Bo' C, et al. Eight-week hempseed oil intervention improves the fatty acid composition of erythrocyte phospholipids and the omega-3 index, but does not affect the lipid profile in children and adolescents with primary hyperlipidemia. Food Res Int. 2019;119:469-476. Excluded: Substance not on the medicine-food homology catalogue.

13. Demonty I, Lin Y, Zebregs YEMP, Vermeer MA, Van Der Knaap HCM, Jakel M, et al. The citrus flavonoids hesperidin and naringin do not affect serum cholesterol in moderately hypercholesterolemic men and women. Journal of Nutrition. 2010;140(9):1615-1620. Excluded: Substance not on the medicine-food homology catalogue.

14. Derakhshandeh-Rishehri SM, Heidari-Beni M, Feizi A, Askari GR, Entezari MH. Effect of honey vinegar syrup on blood sugar and lipid profile in healthy subjects. International Journal of Preventive Medicine. 2014;5(12):1608-1615. Excluded: Multi-ingredient product with a non-catalogue active ingredient.

15. Ghaffarzadegan T, Zanzer YC, Östman E, Hållenius F, Essén S, Sandahl M, et al. Postprandial Responses of Serum Bile Acids in Healthy Humans after Ingestion of Turmeric before Medium/High-Fat Breakfasts. Molecular nutrition & food research. 2019;63(21). Excluded: Mechanistic, pharmacokinetic, or safety-only outcome.

16. Ghanbari-Niaki A, Saeidi A, Kolahdouzi S, Aliakbari-Baydokhty M, Ardeshiri S, Abderrahman AB, et al. Effect of Crocus Sativus Linnaeus (saffron) supplementations combined with circuit resistance training on total creatine kinase, lactate dehydrogenase and creatine kinase MB levels in young untrained men. Science and Sports. 2019;34(1):e53-e58. Excluded: Primary outcome not cardiometabolic.

17. Guo M, Wang P, Du J, Fu C, Yang Q, Gao Z, et al. Xinyue Capsule in patients with stable coronary artery disease after percutaneous coronary intervention: a multicenter, randomized, placebo-controlled trial. Pharmacological Research. 2020;158. Excluded: Species or edible plant part not in the catalogue.

18. Gutierrez-Hellin J, Aguilar-Navarro M, Ruiz-Moreno C, Munoz A, Amaro-Gahete FJ, Posada-Ayala M, et al. Effect of p-Synephrine on Fat Oxidation Rate during Exercise of Increasing Intensity in Healthy Active Women. Nutrients. 2022;14(20). Excluded: Substance not on the medicine-food homology catalogue.

19. Haidari F, Heybar H, Jalali MT, Ahmadi Engali K, Helli B, Shirbeigi E. Hesperidin Supplementation Modulates Inflammatory Responses Following Myocardial Infarction. Journal of the American College of Nutrition. 2015;34(3):205-211. Excluded: Substance not on the medicine-food homology catalogue.

20. Haldar S, Chia SC, Lee SH, Lim J, Leow MKS, Chan ECY, et al. Polyphenol-rich curry made with mixed spices and vegetables benefits glucose homeostasis in Chinese males (Polyspice Study): a dose-response randomized controlled crossover trial. European Journal of Nutrition. 2019;58(1):301-313. Excluded: Substance not on the medicine-food homology catalogue.

21. Hendre AS, Patil SR, Sontakke AV, Phatak RS. Ameliorating Effect of Turmeric on Kidney Function in Patients with Type 2 Diabetes Mellitus. International Journal of Pharmaceutical Sciences and Research. 2022;13(10):4019-4024. Excluded: Primary outcome not cardiometabolic.

22. Hernandez-Garcia D, Granado-Serrano AB, Martin-Gari M, Ensenyat A, Naudi A, Serrano JCE. Short-Term Panax Ginseng Extract Supplementation Reduces Fasting Blood Triacylglycerides and Oxygen Consumption during Sub-Maximal Aerobic Exercise in Male Recreational Athletes. Biomolecules. 2024;14(5). Excluded: Multi-ingredient product with a non-catalogue active ingredient.

23. Holscher HD, Doligale JL, Bauer LL, Gourineni V, Pelkman CL, Fahey GC, et al. Gastrointestinal tolerance and utilization of agave inulin by healthy adults. Food & function. 2014;5(6):1142-1149. Excluded: Species or edible plant part not in the catalogue.

24. Homayouni F, Haidari F, Hedayati M, Zakerkish M, Ahmadi K. Hesperidin Supplementation Alleviates Oxidative DNA Damage and Lipid Peroxidation in Type 2 Diabetes: A Randomized Double-Blind Placebo-Controlled Clinical Trial. Phytotherapy Research. 2017;31(10):1539-1545. Excluded: Substance not on the medicine-food homology catalogue.

25. Iemitsu K, Fujie S, Uchida M, Inoue K, Shinohara Y, Iemitsu M. Dioscorea esculenta Intake with Resistance Training Improves Muscle Quantity and Quality in Healthy Middle-Aged and Older Adults: A Randomized Controlled Trial. Nutrients. 2023;15(11). Excluded: Species or edible plant part not in the catalogue.

26. Jimenez-Osorio AS, Garcia-Nino WR, Gonzalez-Reyes S, Alvarez-Mejia AE, Guerra-Leon S, Salazar-Segovia J, et al. The Effect of Dietary Supplementation With Curcumin on Redox Status and Nrf2 Activation in Patients With Nondiabetic or Diabetic Proteinuric Chronic Kidney Disease: A Pilot Study. Journal of renal nutrition : the official journal of the Council on Renal Nutrition of the National Kidney Foundation. 2016;26(4):237-244. Excluded: Primary outcome not cardiometabolic.

27. Jung DH, Lee YJ, Kim CB, Kim JY, Shin SH, Park JK. Effects of ginseng on peripheral blood mitochondrial DNA copy number and hormones in men with metabolic syndrome: A randomized clinical and pilot study. Complementary Therapies in Medicine. 2016;24:40-46. Excluded: Multi-ingredient product with a non-catalogue active ingredient.

28. Jung SJ, Hwang JH, Park SH, Choi EK, Ha KC, Baek HI, et al. A 12-week, randomized, double-blind study to evaluate the efficacy and safety of liver function after using fermented ginseng powder (GBCK25). Food and Nutrition Research. 2020;64. Excluded: Primary outcome not cardiometabolic.

29. Jung SJ, Park EO, Chae SW, Lee SO, Kwon JW, You JH, et al. Effects of Unripe Black Raspberry Extract Supplementation on Male Climacteric Syndrome and Voiding Dysfunction: A Pilot, Randomized, Double-Blind, Placebo-Controlled Trial. Nutrients. 2023;15(15). Excluded: Substance not on the medicine-food homology catalogue.

30. Jung SM, Haddad EH, Kaur A, Sirirat R, Kim AY, Oda K, et al. A non-probiotic fermented soy product reduces total and LDL cholesterol: a randomized controlled crossover trial. Nutrients. 2021;13(2):535. Excluded: Substance not on the medicine-food homology catalogue.

31. Karimi A, Naeini F, Niazkar HR, Tutunchi H, Musazadeh V, Mahmoodpoor A, et al. Nano-curcumin supplementation in critically ill patients with sepsis: a randomized clinical trial investigating the inflammatory biomarkers, oxidative stress indices, endothelial function, clinical outcomes and nutritional status. Food & function. 2022;13(12):6596-6612. Excluded: Primary outcome not cardiometabolic.

32. Khajehdehi P, Zanjaninejad B, Aflaki E, Nazarinia M, Azad F, Malekmakan L, et al. Oral Supplementation of Turmeric Decreases Proteinuria, Hematuria, and Systolic Blood Pressure in Patients Suffering From Relapsing or Refractory Lupus Nephritis: A Randomized and Placebo-controlled Study. Journal of Renal Nutrition. 2012;22(1):50-57. Excluded: Primary outcome not cardiometabolic.

33. Kim SW, Ha KC, Choi EK, Jung SY, Kim MG, Kwon DY, et al. The effectiveness of fermented turmeric powder in subjects with elevated alanine transaminase levels: A randomised controlled study. BMC Complementary and Alternative Medicine. 2013;13. Excluded: Primary outcome not cardiometabolic.

34. Kim SY, Seo SK, Choi YM, Jeon YE, Lim KJ, Cho S, et al. Effects of red ginseng supplementation on menopausal symptoms and cardiovascular risk factors in postmenopausal women: A double-blind randomized controlled trial. Menopause. 2012;19(4):461-466. Excluded: Primary outcome not cardiometabolic.

35. Koonyosying P, Kusirisin W, Kusirisin P, Kasempitakpong B, Sermpanich N, Tinpovong B, et al. Perilla Fruit Oil-Fortified Soybean Milk Intake Alters Levels of Serum Triglycerides and Antioxidant Status, and Influences Phagocytotic Activity among Healthy Subjects: A Randomized Placebo-Controlled Trial. Nutrients. 2022;14(9). Excluded: Multi-ingredient product with a non-catalogue active ingredient.

36. Kulkarni RA, Deshpande AR. Anti-inflammatory and antioxidant effect of ginger in tuberculosis. Journal of Complementary and Integrative Medicine. 2016;13(2):201-206. Excluded: Primary outcome not cardiometabolic.

37. Kume D, Nishiwaki M, Ito M. Effect of Powdered Morus australis Leaves on Arterial Stiffness Response after Sucrose Ingestion in Healthy Young Men: A Pilot Study. Journal of Nutritional Science and Vitaminology. 2025;71(1):63-69. Excluded: Species or edible plant part not in the catalogue.

38. Larmo P, Alin J, Salminen E, Kallio H, Tahvonen R. Effects of sea buckthorn berries on infections and inflammation: A double-blind, randomized, placebo-controlled trial. European Journal of Clinical Nutrition. 2008;62(9):1123-1130. Excluded: Primary outcome not cardiometabolic.

39. Lee MS, Wahlqvist ML, Chou YC, Fang WH, Lee JT, Kuan JC, et al. Turmeric improves post-prandial working memory in pre-diabetes independent of insulin. Asia Pacific Journal of Clinical Nutrition. 2014;23(4):581-591. Excluded: Primary outcome not cardiometabolic.

40. Lee YJ, Ahn Y, Kwon O, Lee MY, Lee CH, Lee S, et al. Dietary Wolfberry Extract Modifies Oxidative Stress by Controlling the Expression of Inflammatory mRNAs in Overweight and Hypercholesterolemic Subjects: A Randomized, Double-Blind, Placebo-Controlled Trial. Journal of Agricultural and Food Chemistry. 2017;65(2):309-316. Excluded: Mechanistic, pharmacokinetic, or safety-only outcome.

41. Lehtonen HM, Suomela JP, Tahvonen R, Yang B, Venojarvi M, Viikari J, et al. Different berries and berry fractions have various but slightly positive effects on the associated variables of metabolic diseases on overweight and obese women. European Journal of Clinical Nutrition. 2011;65(3):394-401. Excluded: Substance not on the medicine-food homology catalogue.

42. Lin YK, Chung YM, Yang HT, Lin YH, Hu WC, Chiang CF. The potential of immature poken (Citrus reticulata) extract in the weight management, lipid and glucose metabolism. Journal of Complementary and Integrative Medicine. 2022;19(2):273-279. Excluded: Species or edible plant part not in the catalogue.

43. Mammen RR, Natinga Mulakal J, Mohanan R, Maliakel B, Illathu Madhavamenon K. Clove bud polyphenols alleviate alterations in inflammation and oxidative stress markers associated with binge drinking: A randomized double-blinded placebo-controlled crossover study. Journal of Medicinal Food. 2018;21(11):1188-1196. Excluded: Primary outcome not cardiometabolic.

44. Matsuoka T, Yamaji A, Kurosawa C, Shinohara M, Takayama I, Nakagomi H, et al. Co-ingestion of traditional Japanese barley mixed rice (Mugi gohan) with yam paste in healthy Japanese adults decreases postprandial glucose and insulin secretion in a randomized crossover trial. Asia Pac J Clin Nutr. 2023;32(1):40-47. Excluded: Substance not on the medicine-food homology catalogue.

45. Moazen-Zadeh E, Abbasi SH, Safi-Aghdam H, Shahmansouri N, Arjmandi-Beglar A, Hajhosseinn Talasaz A, et al. Effects of Saffron on Cognition, Anxiety, and Depression in Patients Undergoing Coronary Artery Bypass Grafting: A Randomized Double-Blind Placebo-Controlled Trial. Journal of Alternative and Complementary Medicine. 2018;24(4):361-368. Excluded: Primary outcome not cardiometabolic.

46. Modaghegh MH, Shahabian M, Esmaeili HA, Rajbai O, Hosseinzadeh H. Safety evaluation of saffron (Crocus sativus) tablets in healthy volunteers. Phytomedicine. 2008;15(12):1032-1037. Excluded: Primary outcome not cardiometabolic.

47. Morand C, Dubray C, Milenkovic D, Lioger D, Martin JF, Scalbert A, et al. Hesperidin contributes to the vascular protective effects of orange juice: A randomized crossover study in healthy volunteers. American Journal of Clinical Nutrition. 2011;93(1):73-80. Excluded: Species or edible plant part not in the catalogue.

48. Mushtaq S, Irshad G, Bilal AZ. Effect of dried ginger (zingiber officinale) on serum proteins in hyperlipidemic patients. Pakistan Journal of Medical and Health Sciences. 2018;12(2):583-586. Excluded: Primary outcome not cardiometabolic.

49. Nadia FS, Wati DA, Isnawati M, Sulchan M, Afifah DN. The effect of processed tempeh gembus to triglycerides levels and insulin resistance status in women with obesity. Food Res. 2020;4(4):1000-1010. Excluded: Substance not on the medicine-food homology catalogue.

50. Ohara T, Muroyama K, Yamamoto Y, Murosaki S. Oral intake of a combination of glucosyl hesperidin and caffeine elicits an anti-obesity effect in healthy, moderately obese subjects: A randomized double-blind placebo-controlled trial. Nutrition Journal. 2016;15(1). Excluded: Substance not on the medicine-food homology catalogue.

51. Osama H, Hamed EO, Mahmoud MA, Abdelrahim MEA. The effect of hesperidin and diosmin individually or in combination on metabolic profile and neuropathy among diabetic patients with metabolic syndrome: a randomized controlled trial. J Diet Suppl. 2023;20(5):749-762. Excluded: Substance not on the medicine-food homology catalogue.

52. Pakfetrat M, Akmali M, Malekmakan L, Dabaghimanesh M, Khorsand M. Role of turmeric in oxidative modulation in end-stage renal disease patients. Hemodialysis International. 2015;19(1):124-131. Excluded: Primary outcome not cardiometabolic.

53. Park H, Lee E, Kim Y, Jung HY, Kim KM, Kwon O. Metabolic Profiling Analysis Reveals the Potential Contribution of Barley Sprouts against Oxidative Stress and Related Liver Cell Damage in Habitual Alcohol Drinkers. Antioxidants. 2021;10(3). Excluded: Species or edible plant part not in the catalogue.

54. Park SI, Lee S, Lee HY, Yim SV, Kim BH. KGR-BG1, a Standardized Korean Black Ginseng Extract, Has No Significant Effects on Head or Face Temperature Compared with Korean Red Ginseng Extract and a Placebo. Journal of Medicinal Food. 2022;25(6):636-644. Excluded: Primary outcome not cardiometabolic.

55. Rheu KM, Lee BJ, Son WH, Kim DS, Park HT, Ha MS, et al. Effect of Fermented Sarco Oyster (Crassostrea gigas) Extract on Muscle Strength Enhancement in Postmenopausal Females: A Randomized, Double-Blind, Placebo-Controlled Trial. International Journal of Environmental Research and Public Health. 2022;19(24). Excluded: Primary outcome not cardiometabolic.

56. Rodrigues HCN, Martins TFP, Santana NCFES, Braga CC, Silva MAC, Cunha LCD, et al. Antioxidant and anti-inflammatory response to curcumin supplementation in hemodialysis patients: A randomized, double-blind, placebo-controlled clinical trial. Clinical Nutrition ESPEN. 2021;44:136-142. Excluded: Primary outcome not cardiometabolic.

57. Rydell A, Hellsten M, Lindow M, Iggman D. Effectiveness of Written Dietary Advice for Improving Blood Lipids in Primary Care Adults-A Pragmatic Randomized Controlled Trial (MYDICLIN). Nutrients. 2022;14(5). Excluded: Substance not on the medicine-food homology catalogue.

58. Sahraian A, Jelodar S, Javid Z, Mowla A, Ahmadzadeh L. Study the effects of saffron on depression and lipid profiles: A double blind comparative study. Asian Journal of Psychiatry. 2016;22:174-176. Excluded: Primary outcome not cardiometabolic.

59. Salden BN, Troost FJ, De Groot E, Stevens YR, Garces-Rimon M, Possemiers S, et al. Randomized clinical trial on the efficacy of hesperidin 2S on validated cardiovascular biomarkers in healthy overweight individuals1,2. American Journal of Clinical Nutrition. 2016;104(6):1523-1533. Excluded: Substance not on the medicine-food homology catalogue.

60. Schar MY, Curtis PJ, Hazim S, Ostertag LM, Kay CD, Potter JF, et al. Orange juice-derived flavanone and phenolic metabolites do not acutely affect cardiovascular risk biomarkers: a randomized, placebo-controlled, crossover trial in men at moderate risk of cardiovascular disease. Am J Clin Nutr. 2015;101(5):931-938. Excluded: Species or edible plant part not in the catalogue.

61. Shahmansouri N, Farokhnia M, Abbasi SH, Kassaian SE, Noorbala Tafti AA, Gougol A, et al. A randomized, double-blind, clinical trial comparing the efficacy and safety of Crocus sativus L. with fluoxetine for improving mild to moderate depression in post percutaneous coronary intervention patients. Journal of Affective Disorders. 2014;155(1):216-222. Excluded: Primary outcome not cardiometabolic.

62. Smith AJ, Giunta B, Shytle RD, Blum JM. Evaluation of a novel supplement to reduce blood glucose through the use of a modified oral glucose tolerance test. American Journal of Translational Research. 2011;3(2):219-225. Excluded: Multi-ingredient product with a non-catalogue active ingredient.

63. Uchio R, Kawasaki K, Okuda-Hanafusa C, Saji R, Muroyama K, Murosaki S, et al. Curcuma longa extract improves serum inflammatory markers and mental health in healthy participants who are overweight: a randomized, double-blind, placebo-controlled trial. Nutrition Journal. 2021;20(1). Excluded: Primary outcome not cardiometabolic.

64. Viudes DR, Mateus AR, Silva CA, do Carmo Franco M. Curcuma longa improves endothelial glycocalyx integrity and redox-inflammatory pathways in type 2 diabetes mellitus: a randomized double-blind placebo-controlled study. European Journal of Nutrition. 2026;65(3). Excluded: Mechanistic, pharmacokinetic, or safety-only outcome.

65. Wang X, Yang Y, Zheng T, Wang C, Qu S, Luo H, et al. Effect of red ginseng beverage on menopausal symptoms in Chinese women: A randomized, double-blind, placebo-controlled clinical trial. Journal of Ginseng Research. 2026;50(2). Excluded: Primary outcome not cardiometabolic.

66. Whitfield P, Parry-Strong A, Walsh E, Weatherall M, Krebs JD. The effect of a cinnamon-, chromium- and magnesium-formulated honey on glycaemic control, weight loss and lipid parameters in type 2 diabetes: an open-label cross-over randomised controlled trial. European Journal of Nutrition. 2016;55(3):1123-1131. Excluded: Multi-ingredient product with a non-catalogue active ingredient.

67. Woo J, Lau E, Ho SC, Cheng F, Chan C, Chan ASY, et al. Comparison of Pueraria lobata with hormone replacement therapy in treating the adverse health consequences of menopause. Menopause. 2003;10(4):352-361. Excluded: Multi-ingredient product with a non-catalogue active ingredient.

68. Xie F, Chen L, Jin S, Qiu F, Kan J, Wang H, et al. Supplementation with Ginseng, Lilii Bulbus, and Poria induces alterations in the serum metabolic profile of healthy adults. Food & function. 2022;13(18):9602-9609. Excluded: Multi-ingredient product with a non-catalogue active ingredient.

69. Xu X, Lu Q, Wu J, Li Y, Sun J. Impact of extended ginsenoside Rb1 on early chronic kidney disease: a randomized, placebo-controlled study. Inflammopharmacology. 2017;25(1):33-40. Excluded: Primary outcome not cardiometabolic.

70. Xu X, Tian W, Duan W, Pan C, Huang M, Wang Q, et al. Quanduzhong capsules for the treatment of grade 1 hypertension patients with low-to-moderate risk: A multicenter, randomized, double-blind, placebo-controlled clinical trial. Front Pharmacol. 2022;13:1014410. Excluded: Species or edible plant part not in the catalogue.

71. Yang QY, Lu S, Sun HR. Clinical effect of Astragalus granule of different dosages on quality of life in patients with chronic heart failure. Chin J Integr Med. 2011;17(2):146-9. Excluded: Primary outcome not cardiometabolic.

72. Yari Z, Cheraghpour M, Alavian SM, Hedayati M, Eini-Zinab H, Hekmatdoost A. The efficacy of flaxseed and hesperidin on non-alcoholic fatty liver disease: an open-labeled randomized controlled trial. European Journal of Clinical Nutrition. 2021;75(1):99-111. Excluded: Substance not on the medicine-food homology catalogue.

73. Yari Z, Cheraghpour M, Hekmatdoost A. Flaxseed and/or hesperidin supplementation in metabolic syndrome: an open-labeled randomized controlled trial. European Journal of Nutrition. 2021;60(1):287-298. Excluded: Substance not on the medicine-food homology catalogue.
